# Visible light photoflow synthesis of a Cu(ii) single-chain polymer nanoparticle catalyst†

**DOI:** 10.1039/d4sc03079f

**Published:** 2024-08-29

**Authors:** Sebastian Gillhuber, Joshua O. Holloway, Kai Mundsinger, Jochen A. Kammerer, Jeffrey R. Harmer, Hendrik Frisch, Christopher Barner-Kowollik, Peter W. Roesky

**Affiliations:** a Institute of Inorganic Chemistry, Karlsruhe Institute of Technology (KIT) Engesserstraße 15 76131 Karlsruhe Germany roesky@kit.edu; b School of Chemistry and Physics, Queensland University of Technology (QUT) 2 George Street 4000 Brisbane QLD Australia christopher.barnerkowollik@qut.edu.au h.frisch@qut.edu.au; c Centre for Materials Science, Queensland University of Technology (QUT) 2 George Street 4000 Brisbane QLD Australia; d Centre for Advanced Imaging, The University of Queensland (UQ) Building 57 Research Road 4072 Brisbane QLD Australia; e Institute of Nanotechnology (INT), Karlsruhe Institute of Technology (KIT) Hermann-von-Helmholtz-Platz 1 76344 Eggenstein-Leopoldshafen Germany christopher.barner-kowollik@kit.edu

## Abstract

We herein pioneer the visible light (*λ*_max_ = 410 nm) mediated flow synthesis of catalytically active single-chain nanoparticles (SCNPs). Our design approach is based on a copolymer of poly(ethylene glycol) methyl ether methacrylate and a photocleavable 2-((((2-nitrobenzyl)oxy)carbonyl)amino)ethyl methacrylate monomer which can liberate amine groups upon visible light irradiation, allowing for single-chain collapse *via* the complexation of Cu(ii) ions. We initially demonstrate the successful applicability of our design approach for the batch photochemical synthesis of Cu(ii) SCNPs and transfer the concept to photoflow conditions, enabling, for the first time, the continuous production of functional SCNPs. Critically, we explore their ability to function as a photocatalyst for the cleavage of carbon–carbon single and double bonds on the examples of xanthene-9-carboxylic acid and oleic acid, demonstrating the advantageous effect SCNPs can provide over analogous small molecule catalysts.

## Abstract 2

Single-chain nanoparticles (SCNPs) are folded linear polymer chains inspired by the active state of natural enzymes. The current study addresses the synthetic scalability issue of SCNP *via* photoflow synthesis of catalytically active SCNPs.

## Graphical abstract

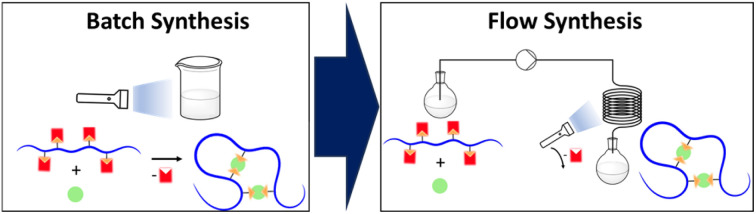

## Introduction

Single-chain nanoparticles (SCNPs) are functional 3D architectures constructed *via* the intramolecular collapse of synthetic macromolecular chains, which mimic the folded state of natural enzymes.^1–5^ In recent years, a plethora of approaches have been explored to achieve this intramolecular chain collapse based on covalent^6–20^ or non-covalent^21–27^ interactions.^2^ The folding units can either be randomly distributed along the polymer chain (repeat unit folding) or be located at predefined positions (selective point folding).^4^

Taking inspiration from naturally occurring metalloenzymes, our groups and others have focused on the synthesis of metal functionalized SCNPs, allowing to synergistically combine the tunable characteristics of polymeric materials with the diverse functionalities of metal complexes.^28–43^ Exploiting the unique opportunities provided by the SCNP environment in catalytic applications has been a major driving force of the field^44–49^ and detailed overviews can be found in the recent literature.^37,50,51^

However, to prevent intermolecular crosslinking throughout SCNP synthesis, the chain folding reactions usually require highly dilute conditions, often significantly below 1 mg mL^−1^,^1^ thus limiting the scalability of SCNP synthesis, and consequently the applicability of SCNPs in general. To overcome the scalability problem, flow synthesis – allowing for the continuous production of SCNPs – is highly desirable. In general, flow chemistry has found a variety of applications in organic and polymer synthesis and offers significant advantages in terms of facile scalability and pathways towards commercial production.^52–58^ In the realm of SCNPs, the groups of Barner-Kowollik and Diesendruck have demonstrated for the first time how transferring a photochemical batch reaction to photoflow conditions can significantly enhance the efficiency of the process, thereby opening a route to access SCNPs on the gram scale.^58^ However, despite the conceivable advantages offered by flow approaches for SCNP synthesis, to the best of our knowledge, no efforts have been undertaken yet to synthesize functional SCNPs under flow conditions.

An ideal system for the flow synthesis of SCNPs allows the mixing of the linear precursor polymer and crosslinker without any immediate initial reaction occurring, thus enabling the simple preparation of one single reaction mixture to be subjected to flow synthesis. During flow synthesis, a suitable trigger must activate either the precursor polymer or the crosslinker, thereby inducing the single-chain collapse. While different triggers are conceivable, including but not limited to thermal, electrochemical or photochemical stimuli, our groups are particularly interested in using light to access highly functional tailor-made macromolecular architectures as light efficiently enables high spatiotemporal control over chemical reactions.^59–62^

The single-chain collapse approach usually used for the synthesis of catalytically active, metal-functionalized SCNPs is based on the coordination of Lewis basic functional groups incorporated in the polymer chain to metal cations. To transfer the approach to flow photochemistry, the Lewis basic groups within the linear precursor polymer need to carry protecting groups^63–70^ to enable mixing with a metal precursor without any immediate reaction occurring. Thus, a copolymer featuring at least one photobasic comonomer constitutes an ideal system for the photoflow synthesis of metal-functionalized single-chain nanoparticles.

Based on these considerations, we herein target a critical gap in the literature by introducing the first synthesis of catalytically active SCNPs under photoflow conditions. Our design approach is based on a poly(ethylene glycol) methyl ether methacrylate polymer backbone, copolymerized with a photocleavable 2-((((2-nitrobenzyl)oxy)carbonyl)amino)ethyl methacrylate monomer, which can liberate amine groups upon visible light irradiation, allowing single-chain collapse by complexation of Cu(ii) ions *via* the liberated amines. Initially, we investigate the photochemical response of the photolabile monomer following our well-established photochemical action plot methodology.^71,72^ Based on the insights gained from the action plot, we show the successful applicability of our concept in batch SCNP synthesis and subsequently demonstrate its transferability to flow conditions using a commercially available photoflow reactor. Critically, we explore the catalytic performance of the resulting Cu(ii) SCNPs for the visible light photocatalyzed cleavage of C–C single and double bonds on the examples of xanthene-9-carboxylic acid and oleic acid, demonstrating the advantageous effect the SCNP environment can provide compared to similar small molecule catalysts.

## Results and discussion

### Monomer photochemical action plot

**Fig. 1:**
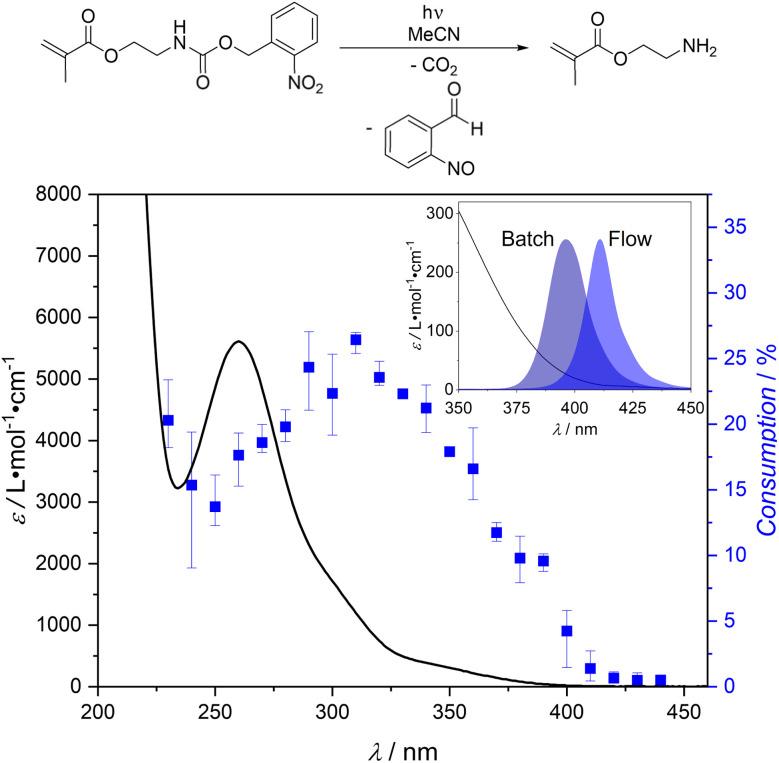
Photochemical action plot of the photocleavage of 2-((((2-nitrobenzyl)oxy)carbonyl)amino)ethyl methacrylate to 2-aminoethyl methacrylate in acetonitrile at a concentration of 0.5 mg mL^−1^, showing the consumption of the starting material upon irradiation with the same number of photons (1.99 × 10^18^ photons) at different wavelengths. Error bars indicate the lowest and highest determined conversion at each wavelength, respectively. Details regarding the photochemical action plot methodology are given in the ESI (Chapter 3).† The insert shows the extinction at high wavelengths, superimposed with the emission spectra of the LEDs employed for the batch and flow synthesis of SCNPs within the current work.

We initially probed the wavelength dependent cleavage response of the literature-known monomer 2-((((2-nitrobenzyl)oxy)carbonyl)amino)ethyl methacrylate, which can liberate Lewis basic amine groups upon light irradiation,^73–75^*via* our photochemical action plot methodology.^71,72^ While the photochemical behaviour of a monomeric system is not necessarily identical to its polymeric counterpart, the investigation of the monomer can still provide valuable insights.^76^ The photochemical action plot obtained for 2-((((2-nitrobenzyl)oxy)carbonyl)amino) ethyl methacrylate in acetonitrile in the wavelength range between 230 nm and 440 nm is shown in Fig. 1 (refer to ESI Chapter 3† for details).

It is evident that the wavelength dependent reactivity pattern is red-shifted by about 50 nm compared to the extinction maximum of 2-((((2-nitrobenzyl)oxy)carbonyl)amino)ethyl methacrylate, an observation previously made for *ortho*-nitrobenzyl photocleavage reactions.^76,77^ Usually, photochemical action plots are employed to determine the wavelength of maximum photoreaction efficiency, which is often strongly disparate from the absorption maximum observed in a UV/vis spectrum.^71,72^ In the current case, we were additionally intrigued by the finding that reactivity was still observed at wavelengths above 400 nm, where the extinction coefficient is significantly below 100 L mol^−1^ cm^−1^ (refer to Fig. 1), indicating that visible light can be employed for the cleavage of our photolabile protecting group.

### SCNP batch synthesis

**Fig. 2:**
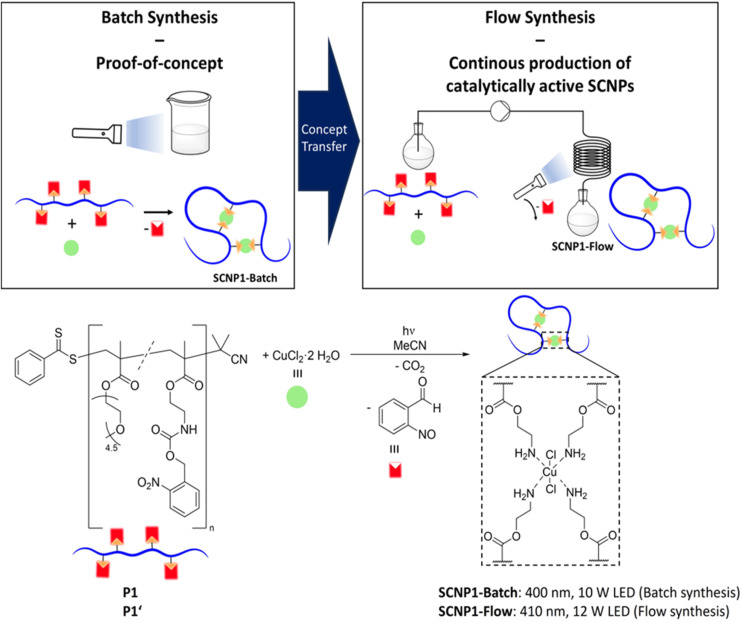
(Top) Schematic illustration of generating functional single-chain nanoparticles (SCNPs) *via* batch and flow synthesis. A linear precursor polymer featuring photobasic amine groups is irradiated in the presence of Cu(ii) ions, forming catalytically active Cu(ii) SCNPs. Transferring the concept from a batch photochemical process to photoflow conditions allows – in principle – for their continuous production. (Bottom) Reaction scheme for the synthesis of Cu(ii) SCNPs. SCNP1-Batch is prepared in a batch photochemical process from the linear precursor polymer P1, SCNP1-Flow from a second batch of the same polymer (P1′) under photoflow conditions. The depiction of the SCNP folding unit is based on a model complex (refer to ESI Fig. S28†).

To incorporate the photolabile monomer into a polymer, it was copolymerized with poly(ethylene glycol) methyl ether methacrylate (average *M*_n_ = 300 g mol^−1^) *via* reversible addition–fragmentation chain-transfer (RAFT) polymerization,^78^ resulting in polymer P1 (refer to Fig. 2). Size exclusion chromatography (SEC) in tetrahydrofuran (THF) gave an indication of the number-averaged molar mass (*M*_n_) of *M*_n_ = 28 200 g mol^−1^ and dispersity (*Đ*) of *Đ* = 1.2. Employing ^1^H nuclear magnetic resonance (NMR) spectroscopy, a content of approximately 14% of incorporated photolabile monomer was determined, in good agreement with the monomer feed ratio (refer to ESI Chapters 4.1 and 4.2†). The targeted polymer is a statistical copolymer; therefore, SCNP folding reactions reported herein follow the repeat unit approach.^4^

To prove that the developed methodology of using a polymer with photobasic functional groups combines access to metal-functionalized SCNPs with temporal control over the folding process, P1 was mixed with copper(ii) chloride dihydrate in acetonitrile at a polymer concentration of 2 mg mL^−1^ and irradiated using a light-emitting diode (LED, centred at 400 nm, refer to Fig. 1 for emission spectrum) in a batch process, resulting in SCNP1-Batch.

**Fig. 3:**
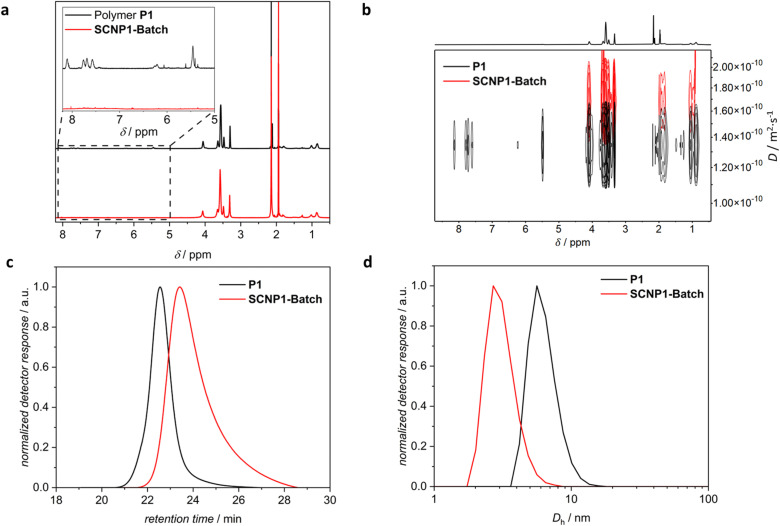
(a) ^1^H NMR spectra (600 MHz, CD_3_CN, 298 K, refer to ESI Chapter 4† for resonance assignments), (b) DOSY NMR spectra (400 MHz, CD_3_CN, 301 K), (c) SEC chromatograms (THF, RI), and (d) number-weighted DLS size distributions (CH_3_CN) of polymer P1 (black) and SCNP1-Batch (red).

^1^H NMR spectroscopy indicated the quantitative deprotection of the linear precursor polymer by disappearance of the resonances associated with the photolabile *ortho*-nitrobenzyl group at *δ* = 8.20–7.45 ppm, 6.35–6.15 ppm and 5.52–5.38 ppm (refer to insert in Fig. 3a). As ^1^H NMR spectroscopy is incapable of differentiating the desired intrachain collapse from undesirable interchain crosslinking, size sensitive techniques were employed to follow the SCNP folding reaction. Comparing the size distributions of P1 and SCNP1-Batch obtained *via* SEC in THF shows that the entire distribution of the latter is shifted towards later retention times, indicating a decrease in the solvodynamic volume, thus validating successful SCNP compaction (refer to Fig. 3c). We note, however, that additional experiments indicated potential enthalpic interactions of the SCNP sample, depending on the SEC columns. To corroborate the observed compaction, dynamic light scattering (DLS) measurements in acetonitrile were conducted, evidencing a decrease in the solvodynamic diameter (*D*_h_), derived from the number-averaged size distributions, from *D*_h_ = 6.4 nm for P1 to *D*_h_ = 3.1 nm for SCNP1-Batch (refer to Fig. 3d), in line with SCNP compaction. We note in passing that DLS data of particles of such small diameters need to be treated with caution due to their low scattering abilities.^5^ Diffusion-ordered ^1^H NMR spectroscopy (DOSY) measurements in CD_3_CN showed an increase in the diffusion coefficient from 1.43 × 10^−10^ m^2^ s^−1^ for P1 to 1.71 × 10^−10^ m^2^ s^−1^ for SCNP1-Batch (refer to Fig. 3b), again in line with the desired SCNP compaction.

Additional control reactions indicate that the simultaneous presence of both CuCl_2_·2H_2_O and light is critical for the successful formation of SCNP1-Batch (refer to ESI Fig. S4†) and that P1 is stable under ambient light conditions and only deprotected under high-intensity LED irradiation (refer to ESI Fig. S2†).

To obtain a more detailed understanding of the structure of the actual SCNP folding unit, a small molecule model complex was synthesized. For that, 2-phenylethylamine was chosen as a small molecule mimic of the ethylamine moieties liberated after cleavage of the *ortho*-nitrobenzyl protecting groups upon irradiation of P1. The mixing of 2-phenylethylamine with CuCl_2_·2H_2_O in acetonitrile resulted in the hexa-coordinated complex [(2-phenylethylamine)_4_CuCl_2_] depicted in ESI Fig. S28.† The central copper ion is coordinated by four amine ligands in a square planar fashion and additionally by two chloride ions, resulting in a Jahn–Teller distorted octahedron, as expected for the d^9^ ion Cu(ii).^79^ We note, however, that the coordination environment within our Cu(ii) folded SCNP may differ from that observed in the model complex due to flexibility constraints within the polymer framework and the presence of additional oxygen atoms. Nonetheless, the model complex unambiguously evidences the capability of ethylamine based ligand systems to bind to Cu(ii) ions under the conditions employed in the synthesis of SCNP1-Batch.

### SCNP flow synthesis

Building on these successful results, we prepared a new batch of polymer (P1′) analogously to P1 for exploration of SCNP synthesis in flow. SEC measurements of P1′ in THF indicated a number-averaged molar mass of *M*_n_ = 30 900 g mol^−1^ and dispersity of *Đ* = 1.2. Using ^1^H NMR spectroscopy, the amount of photolabile functional groups within the copolymer was estimated to be approximately 15% (refer to ESI Chapters 4.1 and 4.2†).

Employing a commercially available photoflow reactor (Vapourtec E-series, refer to ESI Chapter 2.7† for details), a mixture of P1′ and CuCl_2_·2H_2_O in acetonitrile was subjected to irradiation with a 410 nm LED (12 W, refer to Fig. 1 for emission spectrum) under flow conditions at a flow rate of 0.2 mL min^−1^ and a polymer concentration of 2 mg mL^−1^. The tubing volume exposed to light in the photoflow process is 10 mL, resulting in a total irradiation time of 50 minutes, which is similar to the time necessary for the complete deprotection of P1 under batch conditions (refer to ESI Fig. S3†). The complete deprotection of polymer P1′ during the photoflow reaction is evident from ^1^H NMR spectroscopy (refer to ESI Fig. S13†). The resulting SCNP1-Flow was analysed by SEC in THF, showing a shift of the entire molar mass distribution of SCNP1-Flow towards later retention times compared to that of P1′, evidencing successful SCNP compaction (refer to ESI Fig. S18†). SCNP folding was additionally verified by DLS (refer to ESI Fig. S21†), evidencing a decrease in the solvodynamic diameter from *D*_h_ = 6.7 nm for P1′ to *D*_h_ = 4.7 nm for SCNP1-Flow, and DOSY (refer to ESI Fig. S23 and S25†), showing an increase in the diffusion coefficient from 1.47 × 10^−10^ m^2^ s^−1^ for P1′ to 1.56 × 10^−10^ m^2^ s^−1^ for SCNP1-Flow. A comparison of the size data to that of SCNP1-Batch is provided in ESI Chapter 5.10.†

To remove any unreacted CuCl_2_·2H_2_O from the reaction mixture, the solution obtained after the photoflow process was subjected to purification by preparative SEC (refer to ESI Chapter 4.5† for details). The integrity of the polymer during this purification process was verified by SEC measurements in THF (refer to ESI Fig. S18†), and energy dispersive X-ray spectroscopy (EDX) confirmed that Cu is still present in the polymer (refer to ESI Fig. S26†). The actual metal content within SCNP1-Flow was quantified using electron paramagnetic resonance (EPR) spectroscopy (refer to ESI Chapter 5.9† for details). An average Cu(ii) concentration in solutions of SCNP1-Flow of about 0.05 mmol L^−1^ was determined.

To the best of our knowledge, our concept constitutes the first example of a metal functionalized SCNP synthesized under flow conditions. We note that the scalability of the approach presented herein is limited as increasing the polymer concentration or flow rate leads to incomplete deprotection of P1′ and unidentified side reactions (refer to ESI Chapter 4.5†). Nevertheless, the flow approach enables the simple and continuous production of any desired quantity of SCNP1-Flow. Contrary, a batch process would be limited by (i) the difficulty to ensure a homogeneous light distribution through large sample volumes and (ii) the alternative – running several batch reactions in a row – would constitute a time-consuming and inefficient effort.

### Catalysis

**Fig. 4:**
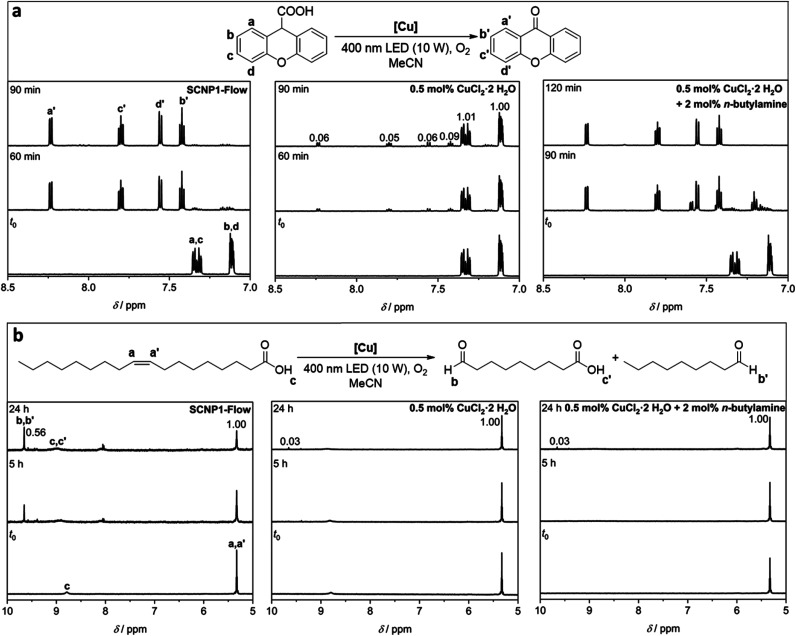
Reaction schemes and ^1^H NMR spectra (600 MHz, CD_3_CN, 298 K) of (a) the photocatalytic decarboxylation and oxygenation of xanthene-9-carboxylic acid and (b) the oxidative cleavage of oleic acid employing SCNP1-Flow (left), CuCl_2_·2H_2_O (centre) or CuCl_2_·2H_2_O in the presence of *n*-butylamine (right) as the catalyst. Resonance labels refer to the respective schemes above the spectra. Numbers on resonances denote integral values.

Having the Cu(ii) functionalized SCNP1-Flow at hand, we employed them for the Cu-catalyzed photocatalytic cleavage of C–C bonds, as recently demonstrated by the groups of Lee and Jiang.^80^ Specifically, we focused on the decarboxylation-oxygenation of xanthene-9-carboxylic acid and the oxidative double bond cleavage of oleic acid (refer to Fig. 4) as we found these reactions to occur without the formation of side products, thus enabling convenient tracking of the reaction progress *via* NMR spectroscopy. The yields obtained in the individual reactions are summarized in ESI Table S12.† At first, the substrates, either xanthene-9-carboxylic acid or oleic acid, respectively, were irradiated with a 400 nm LED (10 W, refer to Fig. 1 for emission spectrum) in the presence of catalytic amounts of SCNP1-Flow (about 0.5 mol% Cu(ii), refer to ESI Chapter 5.9†) under oxygen atmosphere. These catalytic reactions were performed in triplicate (refer to ESI Chapter 6†). Control reactions ensured that the presence of SCNP1-Flow is critical and that neither LED irradiation alone nor the presence of P1′ itself combined with LED irradiation catalyze the product formation. Additional control reactions demonstrate the necessity of light irradiation and prove that purification by preparative SEC efficiently removes unreacted CuCl_2_·2H_2_O after SCNP synthesis (refer to ESI Chapter 6† for all control reactions).

For xanthene-9-carboxylic acid, a quantitative conversion to 9-xanthenone within 90 minutes was observed *via*^1^H NMR spectroscopy (refer to Fig. 4a and ESI Fig. S36–S38†). For the oxidative cleavage of the double bond of oleic acid to nonanal and 9-oxononanoic acid, a conversion close to 40% within 24 hours was observed *via*^1^H NMR spectroscopy (refer to Fig. 4b and ESI Fig. S53–S55†). Increased irradiation times (up to three days) led to a maximum conversion of approximately 50% (refer to ESI Fig. S55†). The same results were obtained when SCNP1-Batch was employed as the catalyst (refer to ESI Fig. S52 and S70†).

It is well established that the specific environment of the active catalyst within SCNPs can offer advantages over analogue small molecule catalysts.^37,50,81–84^ Indeed, conducting the catalytic reactions with 0.5 mol% of CuCl_2_·2H_2_O instead of SCNP1-Flow resulted in a significantly lower rate of the catalytic transformations. For xanthene-9-carboxylic acid, only a conversion of about 10% was observed within 90 minutes (refer to Fig. 4a and ESI Fig. S39–S41†), in contrast to quantitative conversion when SCNP1-Flow is employed. Similarly, for oleic acid a conversion of less than 5% was observed after 24 hours (refer to Fig. 4b and ESI Fig. S56–S58†), compared to about 40% for SCNP1-Flow. To achieve reaction rates similar to those observed for SCNP1-Flow, the CuCl_2_·2H_2_O load had to be increased 20-fold to 10 mol% (refer to ESI Fig. S42–S44 and S59–S61†).

To rationalize the reason for the drastic difference in catalytic performance between SCNP1-Flow and CuCl_2_·2H_2_O, additional experiments were performed. The characteristic differences between these two catalysts are (i) the presence of a polymeric environment and (ii) the coordination of amine groups to the Cu ions within SCNP1-Flow, which are both absent for CuCl_2_·2H_2_O. To investigate whether the presence of the polymer plays an important role, a homopolymer of poly(ethylene glycol) methyl ether methacrylate (poly(PEGMEMA)), mimicking the polymer backbone of SCNP1-Flow, was synthesized. Running the catalytic reactions at a catalyst load of 0.5 mol% CuCl_2_·2H_2_O in the presence of this polymer did not affect the rate of the catalytic reactions (refer to ESI Fig. S50 and S68†).

**Fig. 5:**
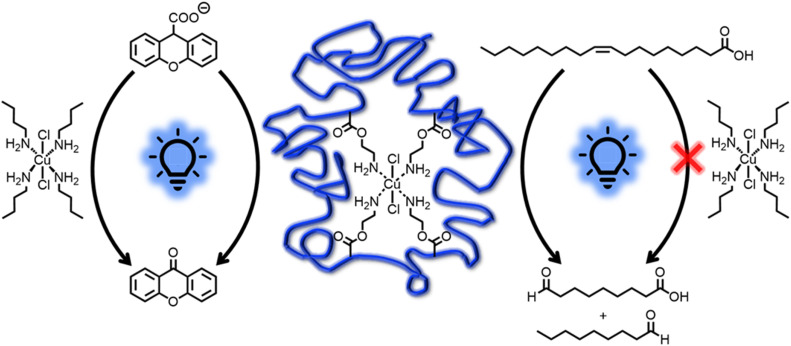
Graphical illustration of the stabilizing effect that the polymeric environment of SCNP1-Flow provides for the catalytically active moiety. While the photocatalytic decarboxylation and oxygenation of xanthene-9-carboxylic acid (left) is catalyzed by SCNP1-Flow as well as tetra(*n*-butylamine) copper(ii) chloride, the latter is inactive in the oxidative cleavage of oleic acid (right).

To mimic the coordination of the Cu ions by primary amine moieties within SCNP1-Flow, the catalytic reactions were conducted again with 0.5 mol% CuCl_2_·2H_2_O in the presence of 2 mol% *n*-butylamine. For xanthene-9-carboxylic acid, this resulted in a drastic increase in the catalytic rate compared to CuCl_2_·2H_2_O itself, and the reaction was completed within 120 minutes (refer to Fig. 4a and ESI Fig. S51†). This clearly shows that the coordination environment of the Cu ions plays a key role and rationalizes the drastic differences in catalytic performance between SCNP1-Flow and CuCl_2_·2H_2_O. In contrast, for oleic acid, no such effect was observed (refer to Fig. 5) and running the reactions with CuCl_2_·2H_2_O in the presence of *n*-butylamine did not increase the rate of the catalytic reaction (refer to Fig. 4b and ESI Fig. S69†). Instead, the presence of *n*-butylamine led to the deprotonation of oleic acid as is evident from the disappearance of the carboxylic acid proton resonance at *δ* = 8.90–8.70 ppm in the ^1^H NMR spectrum of this reaction mixture (refer to Fig. 4b and ESI Fig. S69†). In contrast, when SCNP1-Flow is employed as the catalyst, no deprotonation of oleic acid by the polymer bound amines is observed as is evident from the presence of the carboxylic acid proton resonance at *δ* = 8.90–8.70 ppm in the ^1^H NMR spectra of the corresponding reactions (refer to Fig. 4b and ESI Fig. S53–S55†). It thus seems plausible that the amines remain bonded to the Cu ions, exerting their advantageous effect on the catalytic rate, rationalizing the observed substantial increase in catalytic performance of SCNP1-Flow with respect to CuCl_2_·2H_2_O.

A rigorous rationalization of these findings would require precise knowledge of the p*K*_a_ values of all species present in the reaction solutions. Based on the literature,^85–88^ the situation in acetonitrile is considerably different from that in aqueous solution and strongly depends on delicate equilibria. Further complications may arise from self-assembly or micelle formation upon deprotonation of oleic acid.^89^ These considerations render an in-depth investigation of the observations challenging and beyond the scope of the current study. Nonetheless, our results demonstrate that shielding the catalytic entity within the SCNP environment has a critical and positive effect on catalysis, enabling reactivities unachievable with analogue small molecule catalysts.

## Conclusions

In summary, we pioneer the visible light mediated photoflow synthesis of catalytically active Cu(ii) folded single-chain nanoparticles (SCNPs). Specifically, we investigate the wavelength-resolved reactivity of the photolabile 2-((((2-nitrobenzyl)oxy)carbonyl)amino)ethyl methacrylate monomer and employ it for the synthesis of a poly(ethylene glycol) methyl ether methacrylate copolymer featuring photobasic amine groups. Visible light irradiation of the resulting photolabile polymer in the presence of copper(ii) chloride dihydrate in a batch photochemical process gives access to SCNP1-Batch, constituting the first example of visible-light mediated metal-induced SCNP compaction. Building on these proof-of-concept results, we transfer our chemical approach to a commercially available photoflow reactor, enabling the continuous synthesis of SCNP1-Flow. We demonstrate the activity of the latter in the photocatalytic cleavage of carbon–carbon single and double bonds on the examples of xanthene-9-carboxylic acid and oleic acid, highlighting the advantageous effects the SCNP environment can provide over analogous small molecule catalysts. Our study paves the way for future flow synthetic approaches to the continuous production of functional SCNPs. Critically, our study serves as inspiration for the design of tailor-made catalytic pockets, enabling reactivities unachievable with small molecule catalysts.

## Data availability

Experimental data is available in the ESI.† All synthetic protocols, spectroscopic data, supplementary figures and tables, and detailed crystallographic information can be found in the ESI.† Crystallographic data are available *via* the Cambridge Crystallographic Data Centre (CCDC): 2314460.†

## Author contributions

S. G.: conceptualization, methodology, formal analysis, investigation, writing – original draft, review & editing, visualization, project administration, J. O. H.: conceptualization, writing – review & editing, supervision, project administration, K. M.: writing – review & editing, resources, J. A. K.: investigation (EDX), writing – review & editing, J. R. H.: investigation (EPR), writing – review & editing, H. F.: conceptualization, writing – review & editing, supervision, project administration, C. B.-K.: conceptualization, writing – review & editing, supervision, project administration, funding acquisition, P. W. R.: conceptualization, writing – review & editing, supervision, project administration, funding acquisition.

## Conflicts of interest

There are no conflicts to declare.

## Supplementary Material

SC-OLF-D4SC03079F-s001

SC-OLF-D4SC03079F-s002
