# Supplementary material for: Visible light photoflow synthesis of a Cu(ii) single-chain polymer nanoparticle catalyst
Source: Chem Sci. 2024 Aug 29;15(37):15280–90. doi: 10.1039/d4sc03079f (PMC11376198; doi:10.1039/d4sc03079f)
Supplement: SC-015-D4SC03079F-s001 [file SC-015-D4SC03079F-s001.pdf]

Supplementary Information (SI) for Chemical Science.
This journal is © The Royal Society of Chemistry 2024

Electronic Supporting Information

Visible Light Photoflow Synthesis of a Cu(II) Single-Chain Polymer Nanoparticle Catalyst

Sebastian Gillhuber, Joshua O. Holloway, Kai Mundsinger, Jochen A. Kammerer, Jeffrey R. Harmer, Hendrik Frisch,* Christopher Barner-Kowollik,* and Peter W. Roesky*

Table of Contents

1 Materials and Methods 1

2 Analytical Techniques 2

2.1 Nuclear Magnetic Resonance Spectroscopy 2

2.2 Size Exclusion Chromatography 2

2.3 Dynamic Light Scattering 2

2.4 UV/Vis Spectroscopy 3

2.5 Liquid Chromatography-Mass Spectrometry 3

2.6 Tunable Laser Setup 3

2.7 Photoflow Reactor 4

2.8 Energy Dispersive X-Ray Spectroscopy 4

2.9 Single Crystal X-Ray Diffractometry 5

2.10 Infrared Spectroscopy 5

2.11 Elemental Analysis 5

2.12 Electron Paramagnetic Resonance Spectroscopy 5

3 Action Plot Analysis 6

4 Experimental Details 12

4.1 General Remarks 12

4.2 Synthesis of Polymer P1 and P1' 14

4.3 Synthesis of Poly(PEGMEMA) 16

4.4 Synthesis of SCNP1-Batch 17

4.5 Synthesis of SCNP1-Flow 20

4.6 Synthesis of the Cu(II) Model Complex [(2-phenylethylamine)$_4$CuCl$_2$] 24

5 Analytical Data 25

5.1 Nuclear Magnetic Resonance (NMR) Spectra 25

5.2 Size Exclusion Chromatography (SEC) Data 29

5.3 Dynamic Light Scattering (DLS) Data 31

5.4 Diffusion-Ordered Nuclear Magnetic Resonance (DOSY) Spectra 32

5.5 Energy Dispersive X-Ray Spectroscopy (EDX) of SCNP1-Flow 40

5.6 UV/Vis of 2-((((2-nitrobenzyl)oxy)carbonyl)amino)ethyl methacrylate and LED Emission Spectra 41

5.7 Single-Crystal X-Ray Diffraction (XRD) Data 42

5.8 Infrared Spectroscopy (IR) Data 44

5.9 Electron Paramagnetic Resonance (EPR) Data 45

5.10 Comparison of the Compaction of SCNP1-Batch and SCNP1-Flow 48

6 Catalysis 51
6.1 Decarboxylation-Oxygenation of Xanthene-9-carboxylic acid 51
6.2 Oxidative Double Bond Cleavage of Oleic acid 65
6.3 Summary of Catalytic Performance 80
7 References 81

1 Materials and Methods

Acetonitrile (HPLC grade, Fisher Chemical), acetonitrile-d_3 (99.8 atom% D, Sigma-Aldrich), *n*-butylamine (99 %, Merck), copper(II) chloride dihydrate (99 %, ChemSupply), 2-cyano-2-propyl benzodithioate (97 %, Sigma-Aldrich), diethyl ether (99.5 %, anhydrous, Thermo Fisher Scientific), 1,4-dioxane (99.8 %, anhydrous, Sigma-Aldrich), *n*-pentane (98 %, anhydrous, Thermo Fisher Scientific), oleic acid (90 %, technical grade, Alfa Aesar), 2-phenylethylamine (99 %, Thermo Fisher Scientific), pyrene (99 %, Sigma-Aldrich) and xanthene-9-carboxylic acid (98 %, Combi-Blocks) were used as received. Azobisisobutyronitrile (obtained as 12 wt% solution in acetone, Sigma-Aldrich) was recrystallized from methanol prior to use. Poly(ethylene glycol) methyl ether methacrylate (average M_n 300 g·mol^{-1}, Sigma-Aldrich) was passed over a column of basic alumina (VWR) prior to polymerization. Sephadex LH-20 (Cytiva) was swollen overnight in acetonitrile before use.

2-((((2-Nitrobenzyl)oxy)carbonyl)amino)ethyl methacrylate was prepared according to a literature procedure.[1]

2 Analytical Techniques

2.1 Nuclear Magnetic Resonance Spectroscopy

Nuclear magnetic resonance spectra were recorded on a Bruker Ascend 600 LH or Bruker Avance III 400 MHz spectrometer at 298 K. The temperature was controlled using a Bruker Smart VT unit. All measurements were carried out in deuterated solvents. ^{1}H chemical shifts were referenced internally using the residual solvent resonances and are reported relative to tetramethylsilane. Assignments were determined based on chemical shifts. The multiplicity of polymer resonances is labelled as broad (br).
DOSY experiments were performed at 301 K using a Bruker UltraShield 400 MHz spectrometer. Averaged diffusion coefficients are given in the experimental section. The Bruker pulse sequence *ledbpgp2s* using bipolar gradients with longitudinal eddy current delay and two spoil gradients was used. 32 gradient points were recorded in a linear manner ranging from 2 % to 95 % of the maximum applicable gradient strength. The gradient length (little delta) as well as the diffusion delay (big delta) were optimized prior to each DOSY measurement. The data was processed using TopSpin (Version 3.6.5) and Dynamics Center (Version 2.7.1).

2.2 Size Exclusion Chromatography

Size exclusion chromatography measurements in THF were conducted on a PSS SECurity^2 system consisting of a PSS SECurity Degasser, PSS SECurity TCC6000 Column Oven (35 °C), PSS SDV Column Set (8 x 150 mm 5 µm Precolumn, 8 x 300 mm 5 µm Analytical Columns, 100000 Å, 1000 Å, and 100 Å) and an Agilent 1260 Infinity Isocratic Pump, Agilent 1260 Infinity Standard Autosampler, Agilent 1260 Infinity Diode Array and Multiple Wavelength Detector (A: 254 nm, B: 360 nm), Agilent 1260 Infinity Refractive Index Detector (35 °C). HPLC grade THF, stabilized with BHT, was used as eluent at a flow rate of 1 mL·min^{-1}.
Size exclusion chromatography measurements in DMAc were conducted on a PSS SECurity^2 system consisting of a PSS SECurity Degasser, PSS SECurity TCC6000 Column Oven (60 °C), PSS GRAM Column Set (8 x 150 mm 10 µm Precolumn, 8 x 300 mm 10 µm Analytical Columns, 1000 Å, 1000 Å, and 30 Å) and an Agilent 1260 Infinity Isocratic Pump, Agilent 1260 Infinity Standard Autosampler, Agilent 1260 Infinity Diode Array and Multiple Wavelength Detector (A: 254 nm, B: 360 nm), Agilent 1260 Infinity Refractive Index Detector (35 °C). HPLC grade DMAc containing 0.01 mol·L^{-1} LiBr was used as eluent at a flow rate of 1 mL·min^{-1}.
Narrow disperse linear poly(methyl methacrylate) (M_n: 202 g·mol^{-1} to 2.2x10^6 g·mol^{-1}) standards (PSS ReadyCal) were used as calibrants. All samples were passed through 0.22 µm PTFE membrane filters prior to the measurements. Molecular weight and dispersity analyses were performed with the PSS WinGPC UniChrom software (version 8.33).

2.3 Dynamic Light Scattering

Dynamic light scattering measurements were carried out on a Malvern Zetasizer NanoZS equipped with a 633 nm laser at 298 K. The polymer solutions were prepared by dissolving the samples in acetonitrile

at a concentration of approximately 5 mg·mL^{-1} and passing them through 0.22 µm PTFE membrane filters into 10 mm quartz cells. All measurements were conducted in backscattering mode with an angle of 173° relative to the incident beam. The Malvern Zetasizer software (Version 7.13) was used. Given hydrodynamic diameters (D_h) were averaged over five consecutive measurements.

2.4 UV/Vis Spectroscopy

UV/Vis spectra were recorded on a Shimadzu UV-2700 spectrophotometer equipped with a CPS-100 electronic temperature control cell positioner at 298 K. Samples were dissolved in HPLC grade acetonitrile and measured in Hellma Analytics quartz high precision cells with a path length of 10 mm.

2.5 Liquid Chromatography-Mass Spectrometry

LC-MS measurements were performed on an UltiMate 3000 UHPLC System (Dionex) consisting of a pump (LPG 3400SZ), autosampler (WPS 3000TSL), and a temperature-controlled column compartment (TCC 3000). Separation was performed on a C18 HPLC column (Phenomenex Luna 5 µm, 100 Å, 250 × 2.0 mm) operating at 40 °C. Water (containing 5 mmol·L^{-1} ammonium acetate) and acetonitrile were used as eluents. A gradient of acetonitrile:water, 5:95 to 100:0 (v/v) in 7 min at a flow rate of 0.40 mL·min^{-1} was applied. The flow was split in a 9:1 ratio, where 90 % of the eluent was directed through a DAD UV-detector (VWD 3400, Dionex) and 10 % was infused into the electrospray source. Spectra were recorded on an LTQ Orbitrap Elite mass spectrometer (Thermo Fisher Scientific) equipped with a HESI II probe. The instrument was calibrated in the *m/z* range 74-1822 using premixed calibration solutions (Thermo Scientific). A constant spray voltage of 3.5 kV, a dimensionless sheath gas, and a dimensionless auxiliary gas flow rate of 5 and 2 were applied, respectively. The capillary temperature was set to 300 °C, the S-lens RF level was set to 68, and the aux gas heater temperature was set to 100 °C.

2.6 Tunable Laser Setup

All laser experiments were conducted using the experimental setup shown in **Figure S1**.[2] The light source was an Opotek HE Opolette 355 LD, producing 5 ns pulses with a flattop spatial profile with a 20 Hz repetition rate, tunable from 210 nm to 2400 nm. The output beam was initially passed through a beam expander (-50 mm and 100 mm lens combination) to ensure it is sufficiently large to uniformly irradiate the entire sample volume. The beam then passes through an electronic shutter and is directed upwards using a UV silica right angle prism. Finally, the beam enters the sample, suspended in an aluminium block, from below. The laser energy deposited into the sample was measured above the aluminium block before and after experiments using a Coherent EnergyMax thermopile sensor (J-25MB-LE) to account for any power fluctuations during irradiation. The photon number N_{Ph} reaching the sample in the laser vial at a given wavelength λ_1 can be derived from the set laser pulse energy using the following relation:

$$N_{\mathrm{Ph}} = \frac{T(\lambda_1)}{100}\frac{E_{\mathrm{Pulse}}(\lambda_1)\lambda_1 f_{\mathrm{rep}} t}{hc}$$

where E_{Pulse} is the pulse energy recorded above the aluminum block (at the position of the vial), λ is the wavelength of the incident beam, f_{rep} is the laser repetition rate, t is the total irradiation time, h is Planck's constant, c is the speed of light and $T(\lambda)$ is the wavelength dependent transmission of the laser vial (in %, refer to **Table S1**).
Once an initial measurement is completed and the desired photon number is known, the required energies to obtain the same number of photons in the laser vial at other wavelengths λ_2 in the same total irradiation time can be obtained:

$$E_{\text{Pulse}}(\lambda_2) = E_{\text{Pulse}}(\lambda_1)\frac{\lambda_1}{\lambda_2}\frac{T(\lambda_1)}{T(\lambda_2)}$$

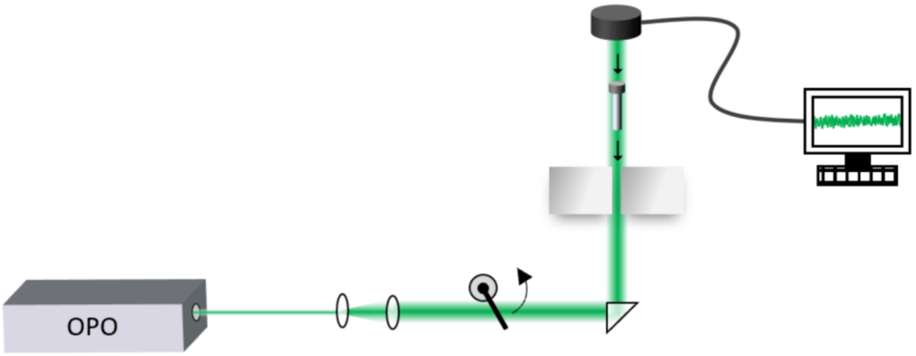

Figure S1 Scheme of the experimental setup for action plot measurements. Light from a monochromatic light source is expanded to cover the entire sample volume, passed through a mechanical shutter and directed onto the sample from below using a prism. The sample is suspended in an opaque aluminum block and the energy delivered to the sample is monitored using an energy meter. Taken from Ref. [2].

2.7 Photoflow Reactor

Photoreactions under flow conditions were performed using a Vapourtec E-series platform with peristaltic pumps fitted with the UV-150 module and the VSD006 cooling module. The module consists of a temperature-controlled irradiation chamber, a transparent fluorinated ethylene polymer (FEP) reactor coil (10 mL, PN: 50-1581) and a LED assembly (390 to 420 nm, peak 410 nm, total power output of 12 W, PN: 50-1444). The temperature is controlled employing pre-cooled nitrogen (heat exchange in the cooling module).

2.8 Energy Dispersive X-Ray Spectroscopy

Energy disperse X-ray spectroscopy (EDX) was carried out in a JEOL JEM-ARM200F ("NeoARM") operating at 200 kV with a spot size of 4 and a 40 µm condenser aperture, resulting in a convergence angle of 24 to 29 mrad and a probe current of 7.48 nA. An EX-24360AHH type EDX detector was used with a total acquisition time of 20 min.
For sample preparation, 5 µL of a solution of **SCNP1-Flow** directly after purification by preparative SEC (refer to Chapter 4.5 for details) were deposited on an ultra-thin carbon film coated Au TEM grid (Electron Microscopy Sciences). Au was chosen to exclude the TEM grid as origin of the detected copper signal. After drying in air, the sample was outgassed at 60 °C under vacuum for several hours.

2.9 Single Crystal X-Ray Diffractometry

Suitable crystals for X-ray analysis were obtained as described in Chapter 4.6. A suitable crystal was covered in mineral oil (Aldrich) and mounted on a glass fibre. The crystal was transferred directly to the cold stream of a STOE StadiVari (100 K) diffractometer. The structure was solved by using the program SHELXS/T[3,4] and Olex2.[5] The remaining non-hydrogen atoms were located from successive difference Fourier map calculations. The refinements were carried out by using full-matrix least-squares techniques on F_0^2 by using the program SHELXL.[3,4] The H-atoms were introduced into the geometrically calculated positions (SHELXL procedures) unless otherwise stated and refined riding on the corresponding parent atoms. The locations of the largest peaks in the final difference Fourier map calculations, as well as the magnitude of the residual electron densities, were of no chemical significance. Summary of the crystal data, data collection and refinement are given in **Table S9**.
Crystallographic data for the structure reported in this paper have been deposited with the Cambridge Crystallographic Data Centre as a supplementary publication no. CCDC 2314460. Copies of the data can be obtained free of charge on application to CCDC, 12 Union Road, Cambridge CB21EZ, UK (fax: (+(44)1223-336-033; email: deposit@ccdc.cam.ac.uk).

2.10 Infrared Spectroscopy

Infrared spectra were recorded in the region 4000-400 cm^{-1} with a resolution of 4 cm^{-1} and 64 scans on a Bruker Alpha II FTIR spectrometer equipped with a room temperature DLaTGS detector, and a diamond attenuated total reflection (ATR) unit. The signals were classified depending on the intensity relative to the most intense peak (vs = very strong (100-75 %), s = strong (75-50 %), m = medium (50-25 %), w = weak (25-0 %)).

2.11 Elemental Analysis

Elemental analyses were carried out with an Elementar Vario MICRO Cube.

2.12 Electron Paramagnetic Resonance Spectroscopy

Continuous-wave (CW) X-band (ca. 9.424 GHz) electron paramagnetic resonance (EPR) spectra were recorded on a Bruker Elexsys E540 spectrometer equipped with an Elexsys Super High Sensitivity Probehead and liquid nitrogen cooling (ER4141 VTM Nitrogen VT Unit). The magnetic field was calibrated with 2,2-diphenyl-1-picrylhydrazyl (g = 2.0036) and measurements were carried out at 115 K using a modulation amplitude of 1.0 mT, a modulation frequency of 100 kHz and a microwave power of 20 mW (10 dB of 200 mW, non-saturating condition). To calculate the EPR intensities, spectra were baseline corrected with a 4th order polynomial and the double integrals calculated numerically. All processing was carried out using MatLab (R2002b).

3 Action Plot Analysis

A stock solution of 2-((((2-nitrobenzyl)oxy)carbonyl)amino)ethyl methacrylate with a concentration of 0.5 mg·mL^{-1} (1.62 mmol·L^{-1}) in acetonitrile was prepared and sparged with argon. For each of the wavelengths given in **Table S1** (230-440 nm in 10 nm increments), 0.4 mL of this stock solution were transferred into a laser vial (0.8 mL, 7 mm x 40 mm, clear glass (for $\lambda \geq 310$ nm) or quartz (for $\lambda \leq 300$ nm)) under inert atmosphere and irradiated for 8 minutes with a monochromatic nanosecond pulsed laser (refer to Chapter 2.6 for details). The energy per pulse was adjusted to keep the total number of photons at each wavelength the same with respect to the total irradiation time (refer to **Table S1** and Chapter 2.6 for details).

Subsequent to irradiation, 0.04 mg of pyrene (0.198 µmol) were added to each vial as internal standard and the resulting solutions subjected to liquid chromatography (refer to Chapter 2.5 for details). Comparing the ratio of the areas (based on the integration of the 254 nm UV detector response) of the peaks referring to the starting material (2-((((2-nitrobenzyl)oxy)carbonyl)amino)ethyl methacrylate) and pyrene with the same ratio for a sample prepared in the same way which has not been irradiated allows for the determination of the amount of consumed starting material upon irradiation (refer to **Table S2** to **S4**). For each wavelength, the experiment was carried out in triplicate and the results are summarized in **Table S2** to **S4**.

Note that the experiments for different wavelengths were conducted on different days. Thus, the preparation of stock solutions and liquid chromatography runs were also conducted on different days. To ensure comparability of the recorded data for all wavelengths, the mean ratio of the peak areas of the starting material and pyrene without irradiation was used as the reference state for zero conversion (refer to **Table S3**). **Table S3** also contains information about the same samples being injected three times on the liquid chromatography column (included in the mean ratio mentioned previously), indicating that statistical errors rather result from sample preparation and pulse energy fluctuations during irradiation than from the liquid chromatography run and subsequent peak integration itself.

Table S1 Photon energies, laser vial transmittance values and energies per pulse for the wavelengths reported in the action plot in the main document. Note that due to the low transmittance values of our clear glass laser vials below 300 nm, quartz laser vials were used instead. Refer to Chapter 2.6 for more details.

Wavelength / nm	Photon energy / 10^{-19} J	Vial Transmittance / %	Energy per pulse / µJ
230	8.64	81.4 (Quartz)	219.4
240	8.28	85.9 (Quartz)	199.4
250	7.95	82.6 (Quartz)	199.1
260	7.64	87.8 (Quartz)	180.0
270	7.36	88.9 (Quartz)	171.2
280	7.09	83.8 (Quartz)	175.2
290	6.85	90.0 (Quartz)	157.5
300	6.62	92.6 (Quartz)	147.9
310	6.41	66.3	200.0
320	6.21	72.7	176.6
330	6.02	77.0	161.7
340	5.84	79.8	151.4
350	5.68	81.7	143.7
360	5.52	83.0	137.6
370	5.37	83.8	132.5
380	5.23	84.4	128.2
390	5.09	84.7	124.3
400	4.97	85.0	120.9
410	4.84	85.1	117.7
420	4.73	85.2	114.8
430	4.62	85.3	112.0
440	4.51	85.4	109.4

Table S2 Relative peak areas of the UV detector responses (254 nm) obtained via integration of the corresponding peaks in the liquid chromatography chromatogram after irradiation of the starting material 2-((((2-nitrobenzyl)oxy)carbonyl)-amino)ethyl methacrylate ($A_{rel,SM}$) and addition of the internal standard pyrene ($A_{rel,IS}$) and their ratio at the wavelengths reported in the action plot in the main document. Each experiment was performed in triplicate.

Wavelength / nm	Relative peak area starting material ($A_{rel,SM}$)	Relative peak area internal standard ($A_{rel,IS}$)	Ratio $A_{rel,SM}/A_{rel,IS}$
230	53.52	46.48	1.15
	55.14	44.86	1.23
	54.81	45.19	1.21
240	54.78	45.22	1.21
	55.31	44.69	1.24
	57.75	42.25	1.37
250	56.87	43.13	1.32
	56.73	43.27	1.31
	55.76	44.24	1.26
260	54.80	45.20	1.21
	55.10	44.90	1.23
	56.01	43.99	1.27
270	54.60	45.40	1.20
	55.21	44.79	1.23
	55.25	44.75	1.23
280	54.25	45.75	1.19
	55.00	45.00	1.22
	54.71	45.29	1.21
290	54.26	45.74	1.19
	53.01	46.99	1.13
	52.30	47.70	1.10
300	54.85	45.15	1.21
	52.88	47.12	1.12
	53.79	46.21	1.16
310	52.32	47.68	1.10
	52.86	47.14	1.12
	52.34	47.66	1.10
320	53.06	46.94	1.13
	53.66	46.34	1.16
	53.65	46.35	1.16
330	53.83	46.17	1.17
	53.93	46.07	1.17
	53.83	46.17	1.17
340	54.17	45.83	1.18
	53.65	46.35	1.16
	54.79	45.21	1.21
350	55.28	44.72	1.24
	55.21	44.79	1.23
	55.20	44.80	1.23

Table S2 continued

360	54.66	45.34	1.21
	56.29	43.71	1.29
	55.82	44.18	1.26
370	56.80	43.20	1.31
	57.04	42.96	1.33
	57.20	42.80	1.34
380	57.09	42.91	1.33
	58.05	41.95	1.38
	57.49	42.51	1.35
390	57.46	42.54	1.35
	57.82	42.18	1.37
	57.54	42.46	1.36
400	58.69	41.31	1.42
	58.60	41.40	1.42
	59.69	40.31	1.48
410	59.38	40.62	1.46
	59.80	40.20	1.49
	59.94	40.06	1.50
420	59.80	40.28	1.48
	59.72	39.93	1.50
	59.92	40.08	1.50
430	59.79	40.21	1.49
	59.99	40.01	1.50
	60.00	40.00	1.50
440	59.95	40.05	1.50
	59.99	40.01	1.50
	59.83	40.17	1.49

Table S3 Relative peak areas of the UV detector responses (254 nm) obtained via integration of the corresponding peaks in the liquid chromatography chromatogram of an unirradiated mixture of the starting material 2-((((2-nitrobenzyl)oxy)-carbonyl)amino)ethyl methacrylate ($A_{rel,SM}$) and internal standard pyrene ($A_{rel,IS}$) and their ratio. Six samples were prepared on different days (Samples 1-6). For three of them (Samples 4-6), liquid chromatography runs were performed three times (Inject 1-3).

Sample		Relative peak area starting material ($A_{rel,SM}$)	Relative peak area internal standard ($A_{rel,IS}$)	Ratio $A_{rel,SM}/A_{rel,IS}$
1		59.92	40.08	1.50
2		60.57	39.43	1.54
3		60.57	39.43	1.54
4	Inject 1	59.80	40.20	1.49
	Inject 2	59.83	40.17	1.49
	Inject 3	59.81	40.19	1.49
5	Inject 1	59.67	40.33	1.48
	Inject 2	59.67	40.33	1.48
	Inject 3	59.66	40.34	1.48
6	Inject 1	60.24	39.76	1.52
	Inject 2	60.27	39.73	1.52
	Inject 3	60.26	39.74	1.52
				Mean: 1.50

Table S4 Average, lowest and highest conversion determined for each wavelength reported in the action plot in the main document derived from the data given in **Table S2** and **Table S3**.

Wavelength / nm	Average conversion / %	Lowest conversion / %	Highest conversion / %
230	20.2	18.1	23.3
240	15.3	9.0	19.3
250	13.6	12.2	16.1
260	17.6	15.2	19.3
270	18.5	17.8	19.9
280	19.7	18.6	21.0
290	24.3	21.0	27.0
300	22.3	19.1	25.3
310	26.4	25.3	26.9
320	23.5	22.9	24.7
330	22.2	22.0	22.4
340	21.2	19.3	22.9
350	17.8	17.7	17.9
360	16.6	14.2	19.7
370	11.7	11.0	12.4
380	9.7	7.8	11.4
390	9.5	8.7	10.0
400	4.2	1.4	5.7
410	1.3	0.4	2.6
420	0.7	0.4	1.1
430	0.4	0.1	1.0
440	0.4	0.1	0.8

4 Experimental Details

4.1 General Remarks

Equivalents referring to polymers given within the synthetic procedures were calculated under neglection of the influence of end groups on the molecular weight. Note that the poly(ethylene glycol) methyl ether methacrylate (PEGMEMA) monomer itself does not have a defined molar mass, thus the average M_n value of 300 g·mol^{-1} was used for the calculations.
Based on this, the amount of 2-((((2-nitrobenzyl)oxy)carbonyl)amino)ethyl methacrylate (ONBMA) moieties in polymers **P1** and **P1'** can be estimated according to:

$$\frac{N_{\text{ONBMA}}}{N_{\text{PEGMEMA}} + N_{\text{ONBMA}}} = \frac{1}{1 + \frac{2}{3} * \frac{Int(\text{PEGMEMA} - \text{OCH}_3)}{Int(\text{ONBMA} - \text{Benzyl})}}$$

resulting in

P1: $\frac{\text{N}_{\text{ONBMMA}}}{\text{N}_{\text{PEGMEMA}}+\text{N}_{\text{ONBMMA}}} = 0.14 \equiv 14\% \frac{N_{\text{ONBMA}}}{N_{\text{PEGMEMA}}+N_{\text{ONBMA}}} = 0.14 \equiv 14\ \%$

and

P1': $\frac{\text{N}_{\text{ONBMMA}}}{\text{N}_{\text{PEGMEMA}}+\text{N}_{\text{ONBMMA}}} = 0.15 \equiv 15\% \frac{N_{\text{ONBMA}}}{N_{\text{PEGMEMA}}+N_{\text{ONBMA}}} = 0.15 \equiv 15\ \%$

with

N_{ONBMA}	Number of 2-((((2-nitrobenzyl)oxy)carbonyl)amino)ethyl methacrylate moieties incorporated in the polymer chain
N_{PEGMEMA}	Number of poly(ethylene glycol) methyl ether methacrylate moieties incorporated in the polymer chain
Int(PEGMEMA-OCH_3)	Value of the integral obtained from integration of the resonance at δ = 3.34-3.24 ppm (referring to the methoxy group of poly(ethylene glycol) methyl ether methacrylate) in the ^{1}H NMR spectrum of **P1**/**P1'**
Int(ONBMA-Benzyl)	Value of the integral obtained from integration of the resonance at δ = 5.52-5.38 ppm (referring to the benzylic protons of the *ortho*-nitrobenzyl functionality) in the ^{1}H NMR spectrum of **P1**/**P1'**

The amount of reactive functional groups within **P1** and **P1'** can thus be estimated as

$$n_{\mathrm{ONBMA}} = \frac{\frac{N_{\mathrm{ONBMA}}}{N_{\mathrm{PEGMEMA}} + N_{\mathrm{ONBMA}}} m_{\mathrm{Polymer}}}{(1 - \frac{N_{\mathrm{ONBMA}}}{N_{\mathrm{PEGMEMA}} + N_{\mathrm{ONBMA}}}) M_{\mathrm{PEGMEMA}} + \frac{N_{\mathrm{ONBMA}}}{N_{\mathrm{PEGMEMA}} + N_{\mathrm{ONBMA}}} M_{\mathrm{ONBMA}}}$$

with

n_{ONBMA}	Amount of photocleavable 2-((((2-nitrobenzyl)oxy)carbonyl)amino)ethyl methacrylate moieties within **P1**/**P1'** in mole
m_{Polymer}	Weighed mass of polymer **P1**/**P1'**
M_{PEGMEMA}	Molar mass of poly(ethylene glycol) methyl ether methacrylate (used herein: M_n = 300 g·mol^{-1})
M_{ONBMA}	Molar mass of 2-((((2-nitrobenzyl)oxy)carbonyl)amino)ethyl methacrylate

4.2 Synthesis of Polymer P1 and P1'

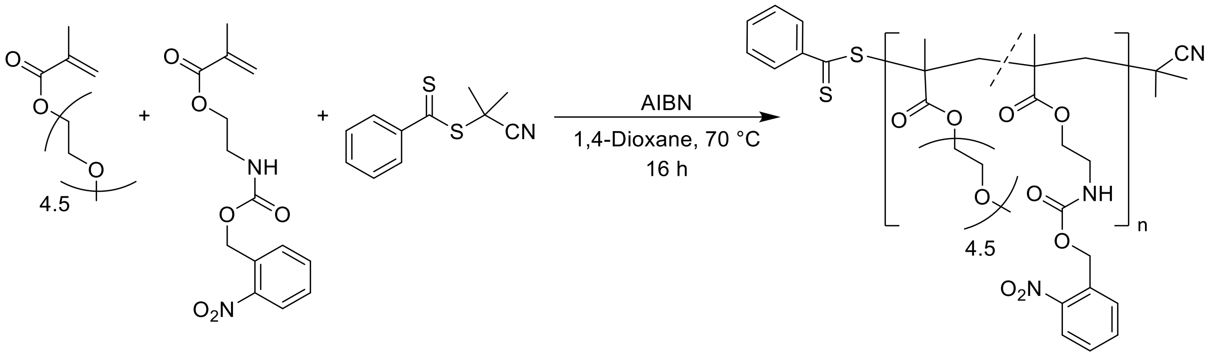

P1/P1'

10.0 mg of the RAFT agent 2-cyano-2-propyl benzodithioate (45.2 µmol, 1.00 eq.) and 1.48 mg azobisisobutyronitrile (AIBN, 9.04 µmol, 0.20 eq.) were dissolved in 5.5 mL of 1,4-dioxane and 2.75 mL of poly(ethylene glycol) methyl ether methacrylate (PEGMEMA, M_n = 300 g·mol^{-1}, 2.89 g, 9.63 mmol, 214 eq.) and 419 mg 2-((((2-nitrobenzyl)oxy)carbonyl)amino)ethyl methacrylate (ONBMA, 1.36 mmol, 30.1 eq.) were added. The solution was degassed by three consecutive freeze-pump-thaw cycles and subsequently heated to 70 °C for 16 h. The reaction was stopped by cooling the flask to room temperature and opening it to air. The polymer was precipitated three times in a mixture of diethyl ether and *n*-pentane (1:1 v/v). The precipitate was isolated by centrifugation and dried under reduced pressure to give **P1** as a pink oil (2.16 g).

A second batch of the polymer (**P1'**) was prepared in the same way.

^{1}H NMR (600 MHz, CD_3CN, 298 K): δ / ppm = 8.20-7.45 (br, ONBMA-*Aryl-H*), 6.35-6.15 (br, ONBMA-N*H*), 5.52-5.38 (br, ONBMA-Benzyl-CH_2), 4.20-3.90 (br, PEGMEMA-COOCH_2), 3.75-3.35 (br, PEGMEMA-OCH_2CH_2), 3.34-3.24 (br, PEGMEMA-OCH_3), 2.05-0.70 (br, aliphatic backbone).

SEC (THF, RI, PMMA cal.): **P1**: M_n = 28,200 g·mol^{-1}, M_w = 34,600 g·mol^{-1}, M_p = 33,500 g·mol^{-1}, $Đ$ = 1.2.
P1': M_n = 30,900 g·mol^{-1}, M_w = 36,500 g·mol^{-1}, M_p = 34,500 g·mol^{-1}, $Đ$ = 1.2.

DLS (CH_3CN): D_h = 6.42 nm (**P1**), 6.66 nm (**P1'**).

DOSY (400 MHz, CD_3CN, 301 K): D = 1.43·10^{-10} m^2·s^{-1} (**P1**), D = 1.47·10^{-10} m^2·s^{-1} (**P1'**).

The stability of the polymer under ambient laboratory conditions was verified by dissolving **P1** in CD_3CN, leaving the NMR tube exposed to ambient light in our laboratories and measuring ^1H NMR spectra immediately after dissolving **P1** in CD_3CN and after 5 hours and 1 day, respectively (refer to **Figure S2**).

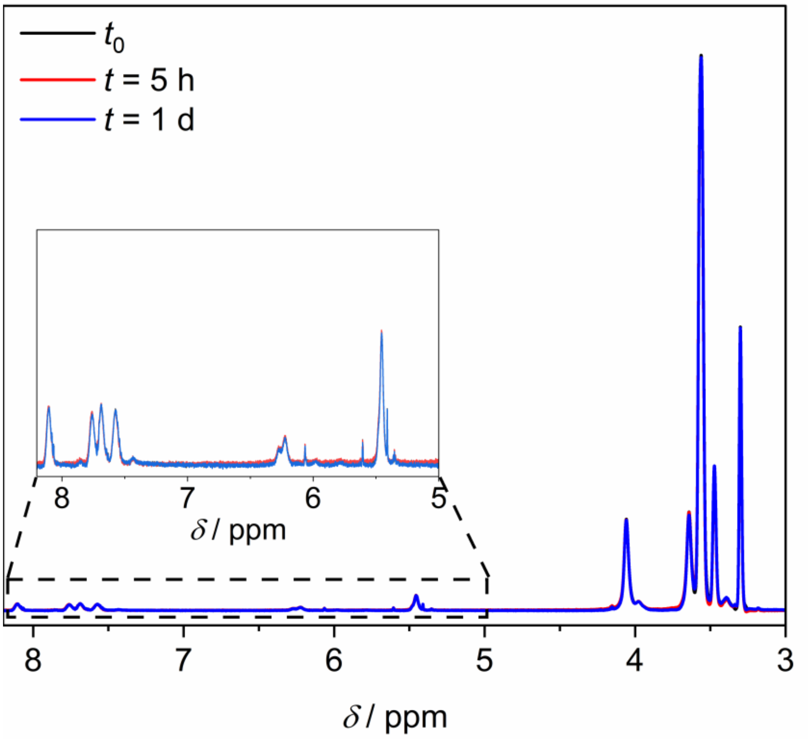

Figure S2 ^1H NMR spectrum (600 MHz, CD_3CN, 298 K) of **P1** immediately after dissolving it in CD_3CN (t_0, black) and after 5 hours (red) and 1 day (blue), respectively, of ambient light exposure.

4.3 Synthesis of Poly(PEGMEMA)

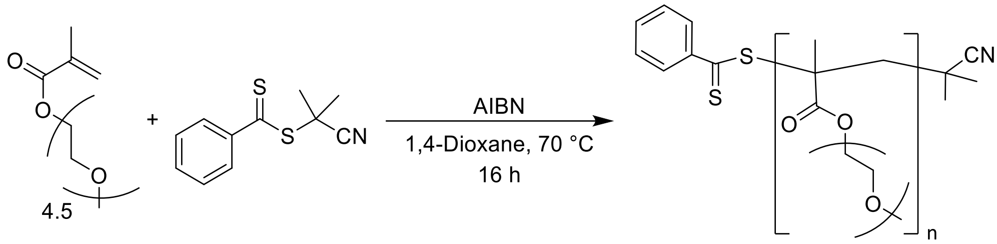

10.0 mg of the RAFT agent 2-cyano-2-propyl benzodithioate (45.2 µmol, 1.00 eq.) and 1.48 mg azobisisobutyronitrile (AIBN, 9.04 µmol, 0.20 eq.) were dissolved in 5.5 mL of 1,4-dioxane and 2.75 mL of poly(ethylene glycol) methyl ether methacrylate (PEGMEMA, M_n = 300 g·mol^{-1}, 2.89 g, 9.63 mmol, 214 eq.) were added. The solution was degassed by three consecutive freeze-pump-thaw cycles and subsequently heated to 70 °C for 16 h. The reaction was stopped by cooling the flask to room temperature and opening it to air. The polymer was precipitated three times in a mixture of diethyl ether and n-pentane (1:1 v/v). The precipitate was isolated by centrifugation and dried under reduced pressure to give Poly(PEGMEMA) as a pink oil (2.15 g).

^{1}H NMR (600 MHz, CD_3CN, 298 K): δ / ppm = 4.14-4.00 (br, COOCH_2), 3.74-3.42 (br, OCH_2CH_2), 3.34-3.24 (br, PEGMEMA-OCH_3), 2.04-0.70 (br, aliphatic backbone).

SEC (THF, RI, PMMA cal.): M_n = 35,000 g·mol^{-1}, M_w = 39,100 g·mol^{-1}, M_p = 36,900 g·mol^{-1}, $Đ$ = 1.1.

4.4 Synthesis of SCNP1-Batch

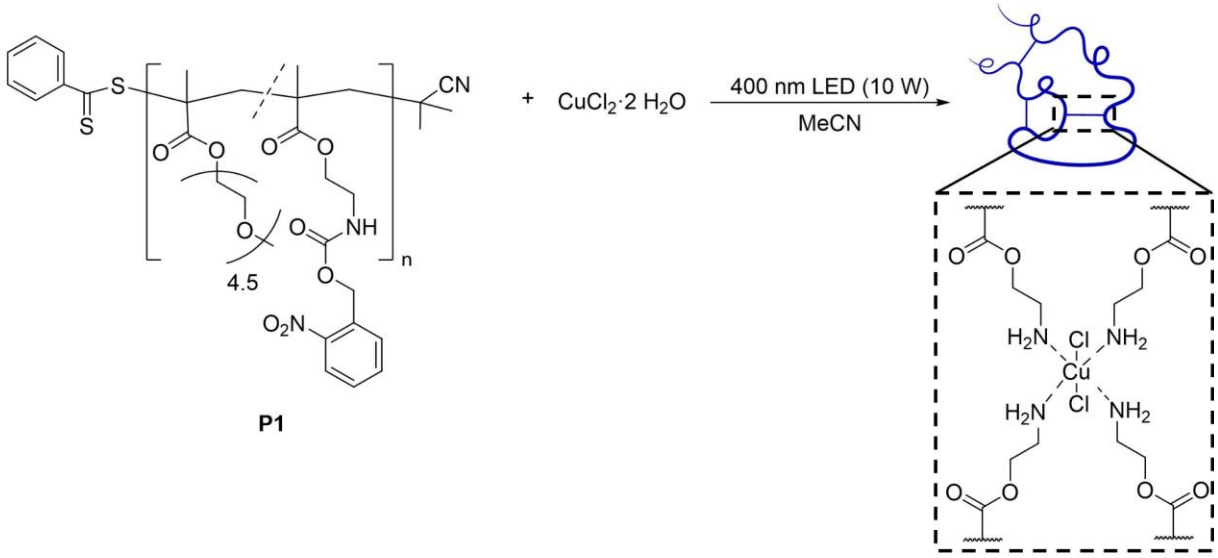

10.0 mg of **P1** (4.65 µmol, 1.00 eq. photoreactive unit) and 0.79 mg of $CuCl_2 \cdot 2\ H_2O$ (4.65 µmol, 1.00 eq.) were dissolved in 5 mL of acetonitrile. The resulting yellow solution was sparged with argon and subsequently irradiated using a 400 nm LED (10 W, refer to **Figure S27** for the emission spectrum). After completion of the reaction (refer to **Figure S3** for kinetic data), the solvent was evaporated under reduced pressure to give **SCNP1-Batch** in quantitative yield.[a]
Note that this workup does not remove unreacted $CuCl_2 \cdot 2\ H_2O$.

^{1}H NMR (600 MHz, CD_3CN, 298 K): δ / ppm = 4.20-3.90 (br, PEGMEMA-COOCH_2), 3.75-3.40 (br, PEGMEMA-OCH_2CH_2), 3.38-3.20 (br, PEGMEMA-OCH_3), 2.00-0.65 (br, aliphatic backbone).

SEC (THF, RI, PMMA cal.): M_n = 9,700 g·mol^{-1}, M_w = 15,200 g·mol^{-1}, M_p = 18,700 g·mol^{-1}, $Đ$ = 1.6.

DLS (CH_3CN): D_h = 3.05 nm.

DOSY (400 MHz, CD_3CN, 301 K): $D = 1.71 \cdot 10^{-10}$ m^2·s^{-1}.

[a] The depiction of the folding unit in the scheme above is based on the model complex depicted in **Figure S28**. The actual coordination environment of the Cu ions within the folding unit is unknown.

Kinetics data (refer to **Figure S3**) for the reaction were obtained in the following way:

10.0 mg of **P1** (4.65 µmol, 1.00 eq. photoreactive unit) and 0.79 mg of $CuCl_2 \cdot 2\ H_2O$ (4.65 µmol, 1.00 eq.) were dissolved in 5 mL of acetonitrile. The resulting yellow solution was sparged with argon and subsequently irradiated using a 400 nm LED (10 W, refer to **Figure S27** for the emission spectrum). 0.1 mL of the reaction mixture were taken out 0, 2, 5, 10, 20, 30, 40 and 50 minutes after starting LED irradiation and added to 0.3 mL of CD_3CN for NMR analysis.

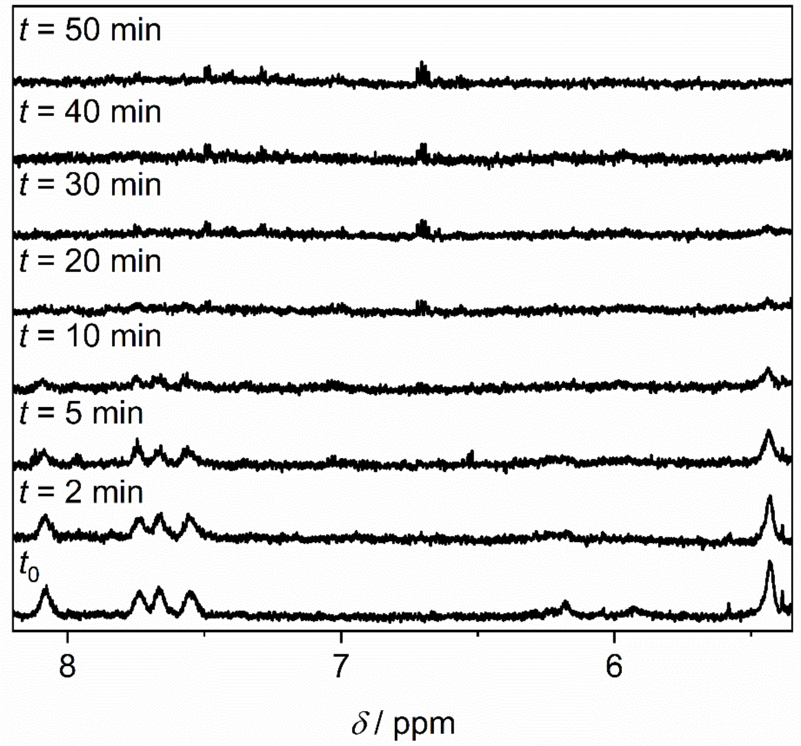

Figure S3 ^1H NMR spectra (600 MHz, CD_3CN, 298 K) of the reaction mixture of **P1** and $CuCl_2 \cdot 2\ H_2O$ at different times after starting the irradiation with a 400 nm LED (10 W, refer to **Figure S27** for the emission spectrum).

To ensure that both, the presence of $CuCl_2$·2 H_2O and light, are crucial for the formation of **SCNP1-Batch**, the following control reactions were conducted (refer to **Figure S4**):

- **P1** + $CuCl_2$·2 H_2O, no irradiation:
10.0 mg of **P1** (4.65 µmol, 1.00 eq. photoreactive unit) and 0.79 mg of $CuCl_2$·2 H_2O (4.65 µmol, 1.00 eq.) were dissolved in 5 mL of acetonitrile. The resulting yellow solution was sparged with argon and subsequently stirred for 50 minutes at room temperature.

- **P1**, irradiation, no $CuCl_2$·2 H_2O:
10.0 mg of **P1** (4.65 µmol, 1.00 eq. photoreactive unit) were dissolved in 5 mL of acetonitrile. The resulting yellow solution was sparged with argon and subsequently irradiated using a 400 nm LED (10 W, refer to **Figure S27** for the emission spectrum) for 50 minutes.

- **P1**, $CuCl_2$·2 H_2O added after irradiation:
10.0 mg of **P1** (4.65 µmol, 1.00 eq. photoreactive unit) were dissolved in 5 mL of acetonitrile. The resulting yellow solution was sparged with argon and subsequently irradiated using a 400 nm LED (10 W, refer to **Figure S27** for the emission spectrum) for 50 minutes. Subsequently, 0.79 mg of $CuCl_2$·2 H_2O (4.65 µmol, 1.00 eq.) were added.

In all cases, the solvent was evaporated under reduced pressure and the resulting residue redissolved in THF for SEC analysis.

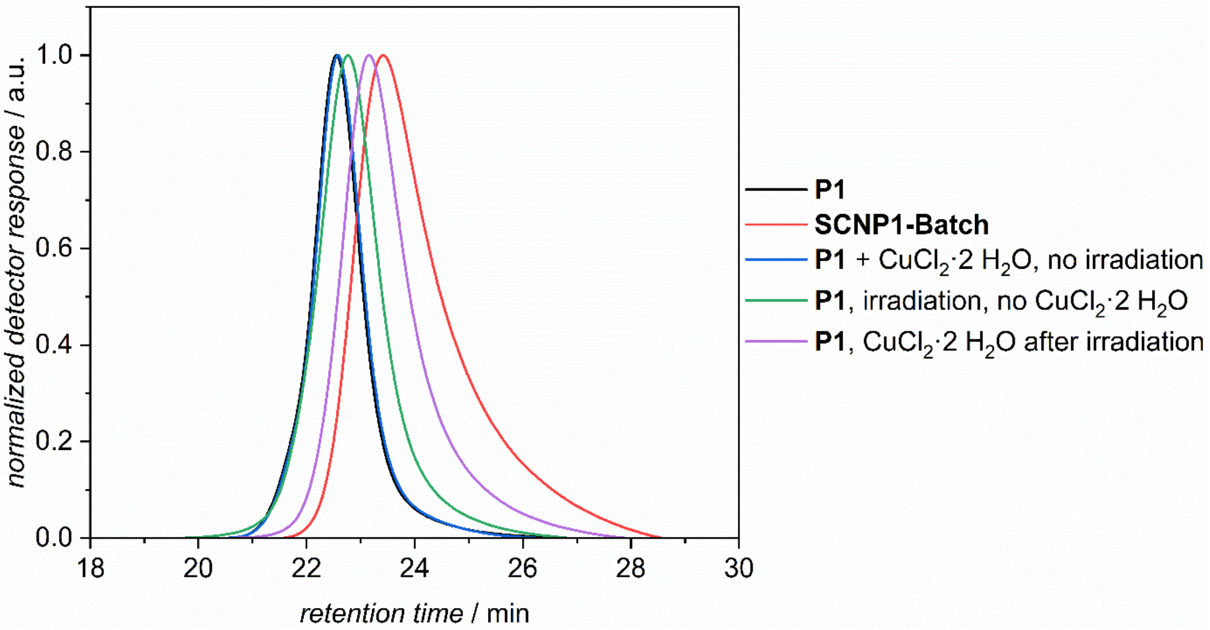

Figure S4 SEC chromatograms (THF, RI) of **P1**, **SCNP1-Batch** and the products of different control reactions.

4.5 Synthesis of SCNP1-Flow

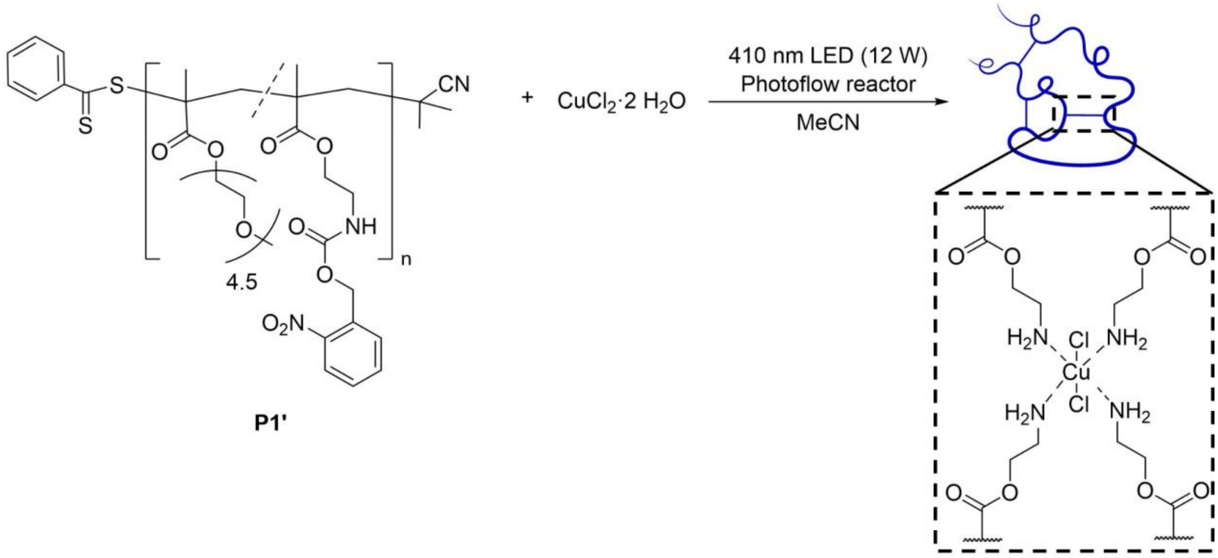

10.0 mg of **P1'** (4.98 µmol, 1.00 eq. photoreactive unit) and 0.84 mg of $CuCl_2·2\ H_2O$ (4.98 µmol, 1.00 eq.) were dissolved in 5 mL of acetonitrile. The resulting yellow solution was sparged with argon and subsequently irradiated using a 410 nm LED (12 W, refer to **Figure S27** for the emission spectrum) under photoflow conditions (flowrate 0.2 mL·min^{-1}, refer to Chapter 2.7 for details regarding the photoflow reactor). The obtained yellow solution was directly loaded onto a preparative SEC column (Sephadex LH-20) and eluted with acetonitrile, resulting in a pale-yellow solution of **SCNP1-Flow**.[a]

^{1}H NMR (600 MHz, CD_3CN, 298 K): δ / ppm = 4.20-3.90 (br, PEGMEMA-COOCH_2), 3.80-3.40 (br, PEGMEMA-OCH_2CH_2), 3.38-3.22 (br, PEGMEMA-OCH_3), 2.00-0.65 (br, aliphatic backbone).

SEC (THF, RI, PMMA cal.): M_n = 16,000 g·mol^{-1}, M_w = 22,200 g·mol^{-1}, M_p = 24,600 g·mol^{-1}, $Đ$ = 1.4.

DLS (CH_3CN): D_h = 4.65 nm.

DOSY (400 MHz, CD_3CN, 301 K): $D = 1.56·10^{-10}$ m^2·s^{-1}.

[a] The depiction of the folding unit in the scheme above is based on the model complex depicted in **Figure S28**. The actual coordination environment of the Cu ions within the folding unit is unknown.

Following the same general procedure as described above, the following experiments were conducted to investigate the influence of flowrate and polymer concentration:

- 10.0 mg of **P1'** (4.98 µmol, 1.00 eq. photoreactive unit) and 0.84 mg of $CuCl_2 \cdot 2\ H_2O$ (4.98 µmol, 1.00 eq.) were dissolved in 5 mL of acetonitrile and the solution sparged with argon. 1 mL aliquots were taken out of this solution and irradiated under photoflow conditions at flowrates of 0.2, 0.3, 0.4, 0.5 and 1 mL·min^{-1}, respectively (refer to **Figure S5** and **S6**).

- 1 mL of acetonitrile solutions with the following amounts of **P1'** and $CuCl_2 \cdot 2\ H_2O$ were prepared from a stock solution and irradiated under photoflow conditions at a flowrate of 0.2 mL·min^{-1} (refer to **Figure S7** and **S8**):

  - 2.0 mg of **P1'** (0.996 µmol, 1.00 eq. photoreactive unit) and 0.17 mg of $CuCl_2 \cdot 2\ H_2O$ (0.996 µmol, 1.00 eq.)
  - 5.0 mg of **P1'** (2.49 µmol, 1.00 eq. photoreactive unit) and 0.42 mg of $CuCl_2 \cdot 2\ H_2O$ (2.49 µmol, 1.00 eq.)
  - 10.0 mg of **P1'** (4.98 µmol, 1.00 eq. photoreactive unit) and 0.84 mg of $CuCl_2 \cdot 2\ H_2O$ (4.98 µmol, 1.00 eq.)
  -20.0 mg of **P1'** (9.96 µmol, 1.00 eq. photoreactive unit) and 1.69 mg of $CuCl_2 \cdot 2\ H_2O$ (9.96 µmol, 1.00 eq.)
  - 50.0 mg of **P1'** (24.9 µmol, 1.00 eq. photoreactive unit) and 4.24 mg of $CuCl_2 \cdot 2\ H_2O$ (24.9 µmol, 1.00 eq.)

For NMR analysis, 0.1 mL of each solution obtained after irradiation were added to 0.3 mL of CD_3CN. For SEC analysis, 0.1 mL of each solution obtained after irradiation were mixed with 0.2 mL of THF and directly injected onto the SEC column.

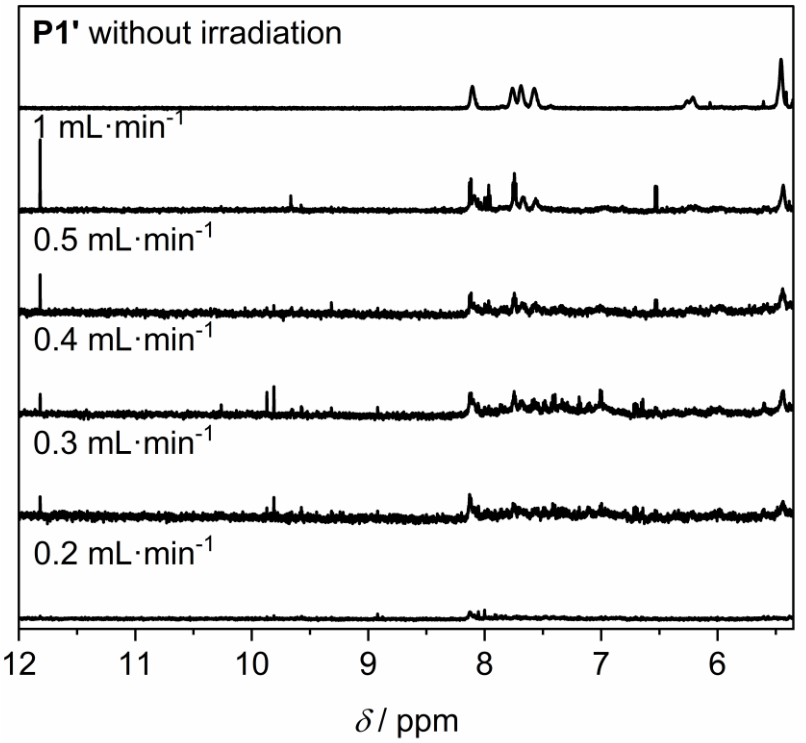

Figure S5 ^{1}H NMR spectra (600 MHz, CD_3CN, 298 K) of the reaction mixtures of **P1’** and $CuCl_2{\cdot}2\ H_2O$ after irradiation with a 410 nm LED (12 W, refer to **Figure S27** for the emission spectrum) under photoflow conditions at different flowrates.

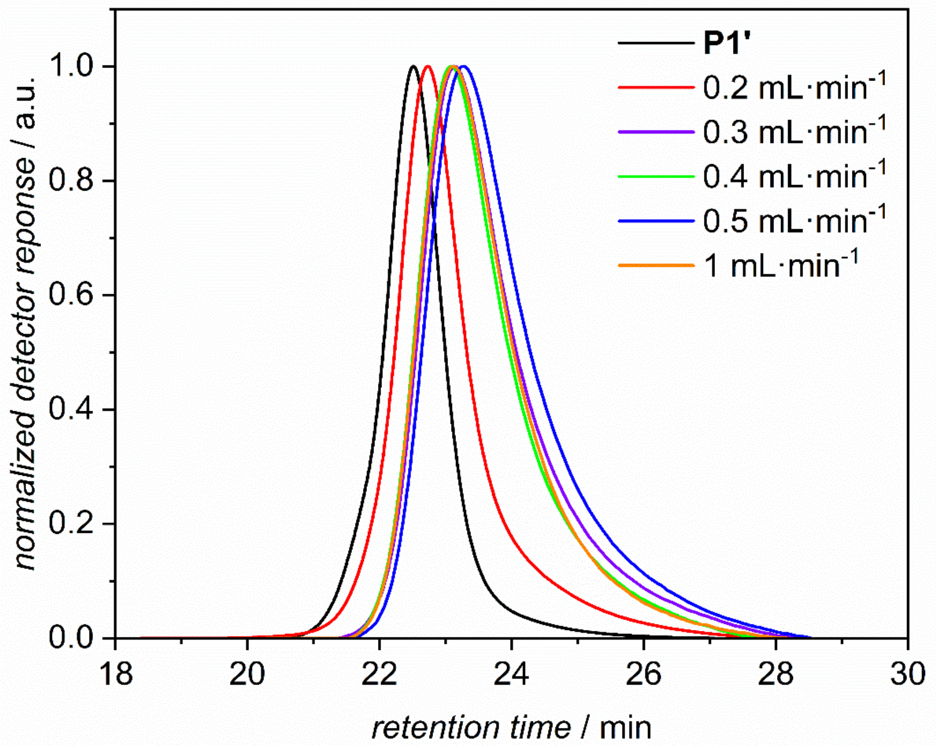

Figure S6 SEC chromatograms (THF, RI) of the reaction mixtures of **P1’** and $CuCl_2{\cdot}2\ H_2O$ after irradiation with a 410 nm LED (12 W, refer to **Figure S27** for the emission spectrum) under photoflow conditions at different flowrates.

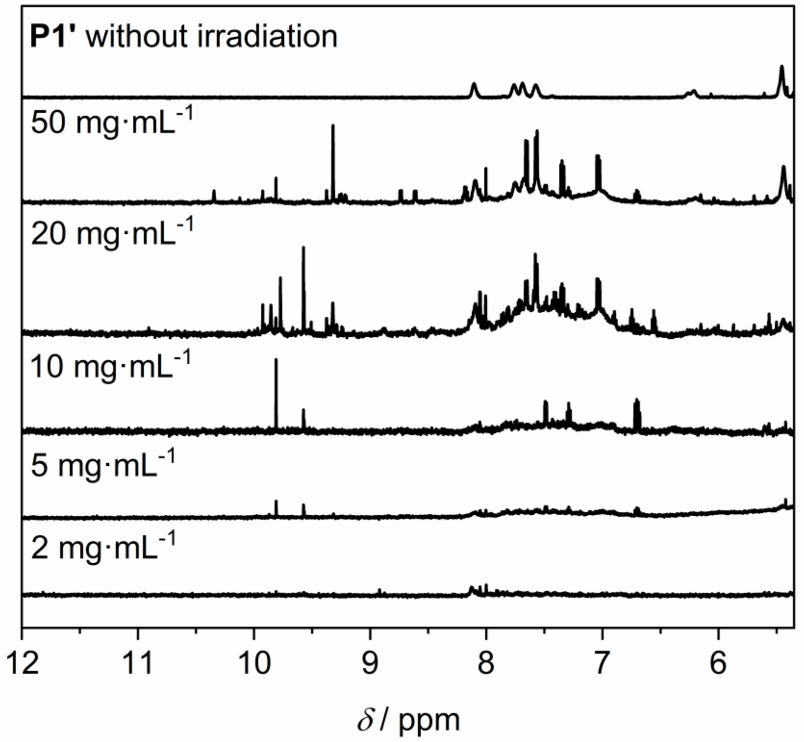

Figure S7 ^{1}H NMR spectra (600 MHz, CD_3CN, 298 K) of the reaction mixtures of **P1’** and $CuCl_2{\cdot}2\ H_2O$ after irradiation with a 410 nm LED (12 W, refer to **Figure S27** for the emission spectrum) under photoflow conditions at different concentrations. Numbers refer to the concentration of **P1’**.

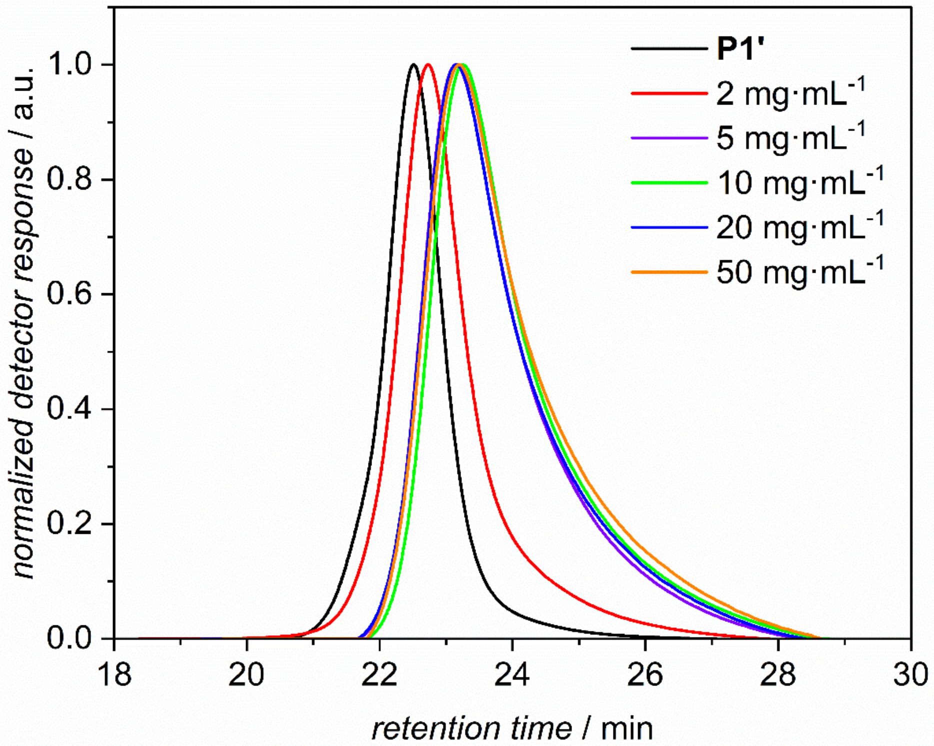

Figure S8 SEC chromatograms (THF, RI) of the reaction mixtures of **P1’** and $CuCl_2{\cdot}2\ H_2O$ after irradiation with a 410 nm LED (12 W, refer to **Figure S27** for the emission spectrum) under photoflow conditions at different concentrations. Numbers refer to the concentration of **P1’**.

4.6 Synthesis of the Cu(II) Model Complex [(2-phenylethylamine)$_4$CuCl$_2$]

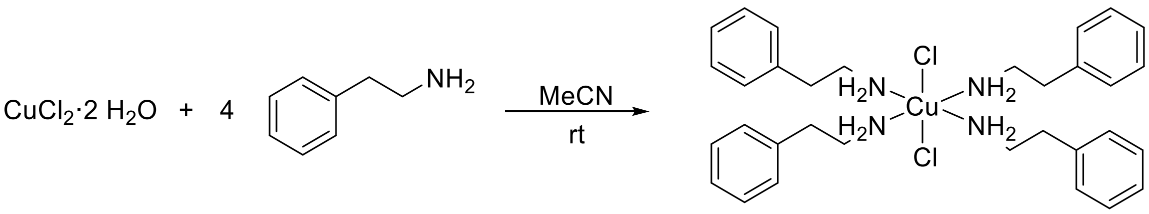

0.500 g of $CuCl_2$·2 H_2O (2.93 mmol, 1.00 eq.) were dissolved in 10 mL of acetonitrile. To the resulting yellow solution, 0.737 mL of 2-phenylethylamine (0.711 g, 5.87 mmol, 2.00 eq.) were added, leading to an immediate color change to dark blue. The solution was subsequently concentrated to approximately 6 mL. After standing at ambient temperature overnight, pale blue needles suitable for single-crystal X-ray diffraction were obtained.

^{1}H NMR (400 MHz, CD_3CN, 298 K): Compound is paramagnetic. Refer to **Figure S14**.

FT-IR (ATR): $\tilde{\nu}$ / cm^{-1} = 3336 (w), 3277 (w), 3207 (w), 3187 (w), 3116 (m), 3020 (w), 3000 (w), 2944 (w), 2913 (w), 2879 (w), 2851 (w), 1600 (m), 1496 (m), 1450 (s), 1445 (m), 1360 (m), 1247 (w), 1224 (w), 1151 (m), 1123 (m), 1078 (m), 1052 (s), 1035 (s), 999 (m), 939 (m), 920 (m), 753 (vs), 699 (vs), 671 (s), 634 (m), 578 (m), 516 (w), 490 (m).

Elemental analysis: [%] calculated for [$C_{36}H_{50}Cl_2CuN_6$] (701.28 g·mol^{-1}): C 62.07, H 7.16, N 9.05; found: C 61.30, H 6.59, N 9.72.

XRD: Refer to **Figure S28** and **Table S9**

5 Analytical Data

5.1 Nuclear Magnetic Resonance (NMR) Spectra

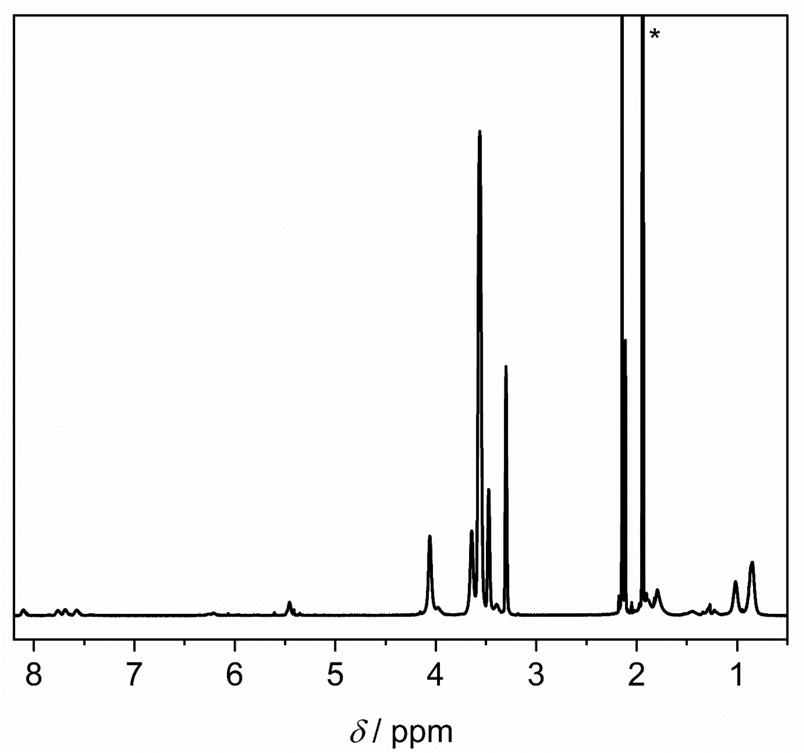

Figure S9 ^{1}H NMR spectrum (600 MHz, CD_3CN, 298 K) of polymer **P1**. * Residual solvent resonance.

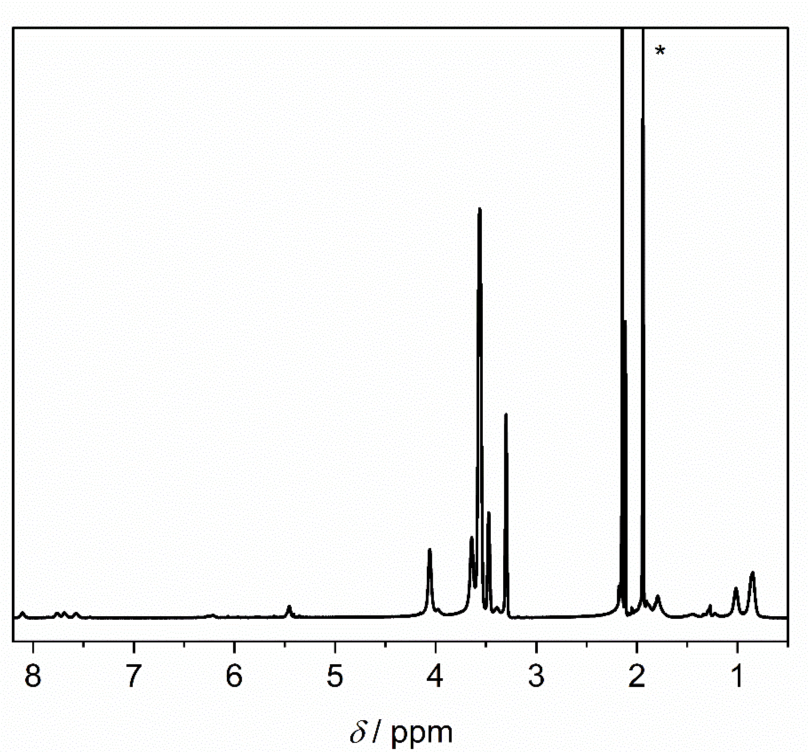

Figure S10 ^{1}H NMR spectrum (600 MHz, CD_3CN, 298 K) of polymer **P1'**. * Residual solvent resonance.

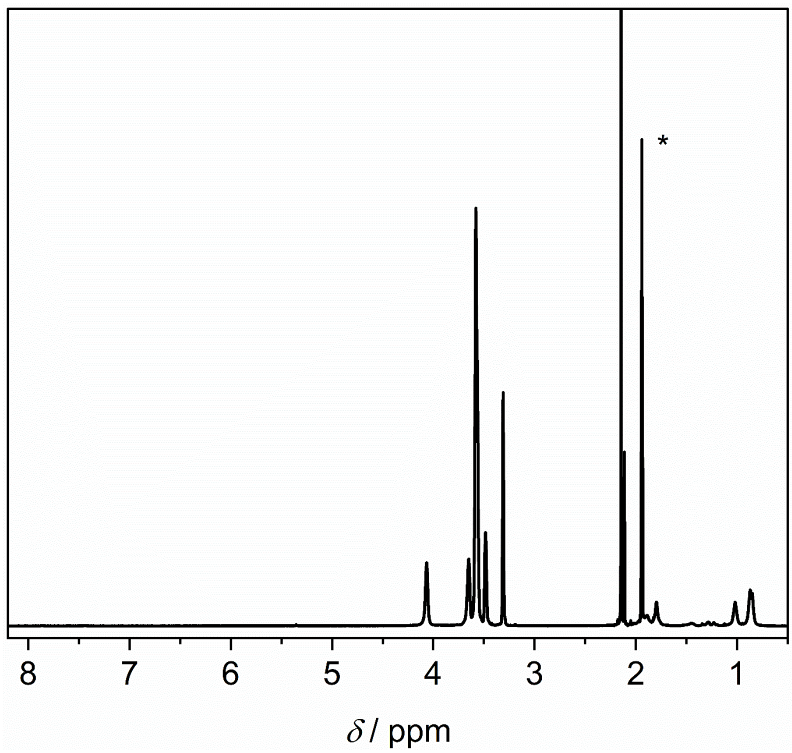

Figure S11 ^{1}H NMR spectrum (600 MHz, CD_3CN, 298 K) of Poly(PEGMEMA). * Residual solvent resonance.

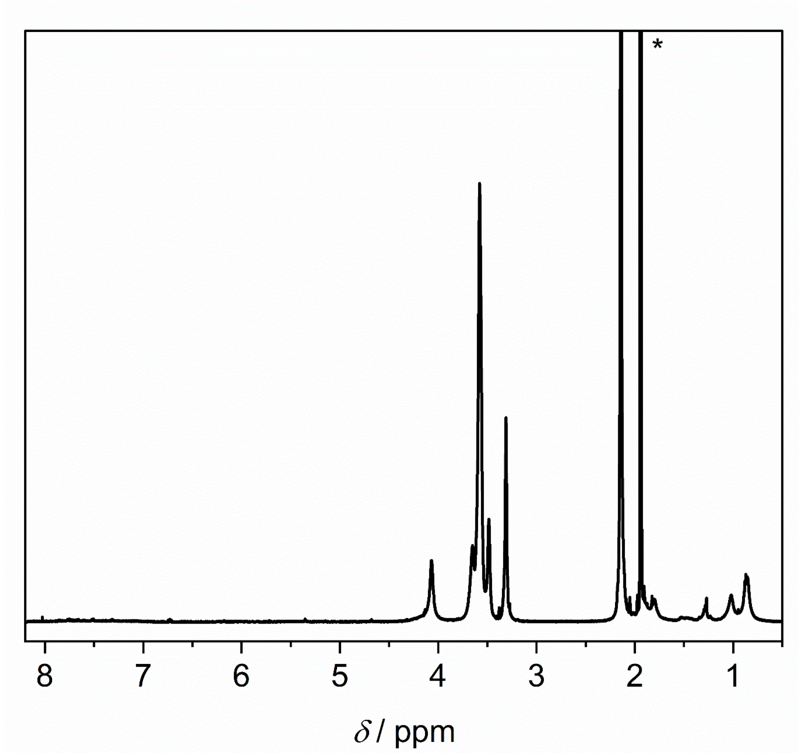

Figure S12 ^{1}H NMR spectrum (600 MHz, CD_3CN, 298 K) of **SCNP1-Batch**. * Residual solvent resonance.

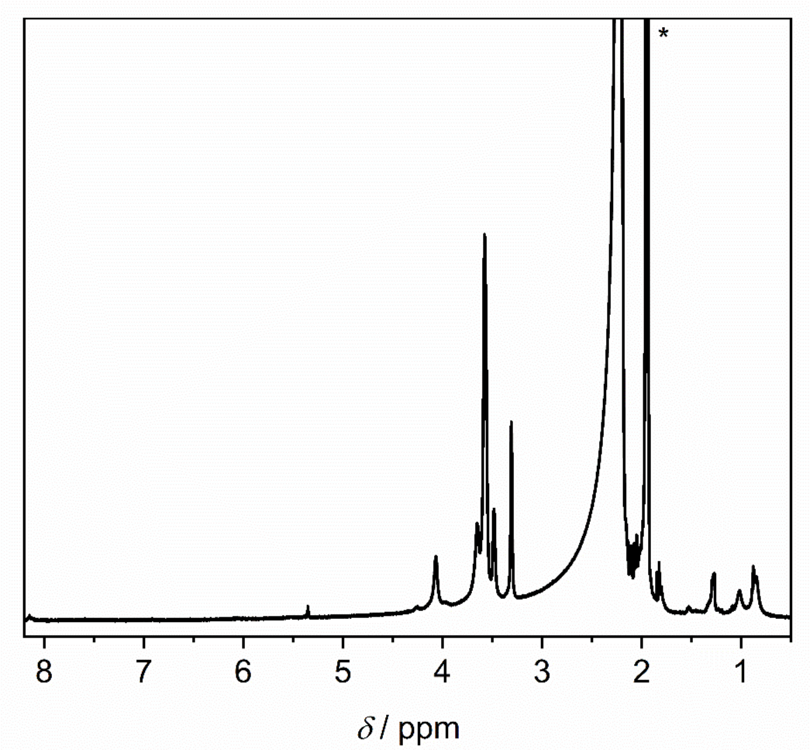

Figure S13 ^{1}H NMR spectrum (600 MHz, CH_3CN/CD_3CN, 298 K) of **SCNP1-Flow**. * Residual solvent resonance.

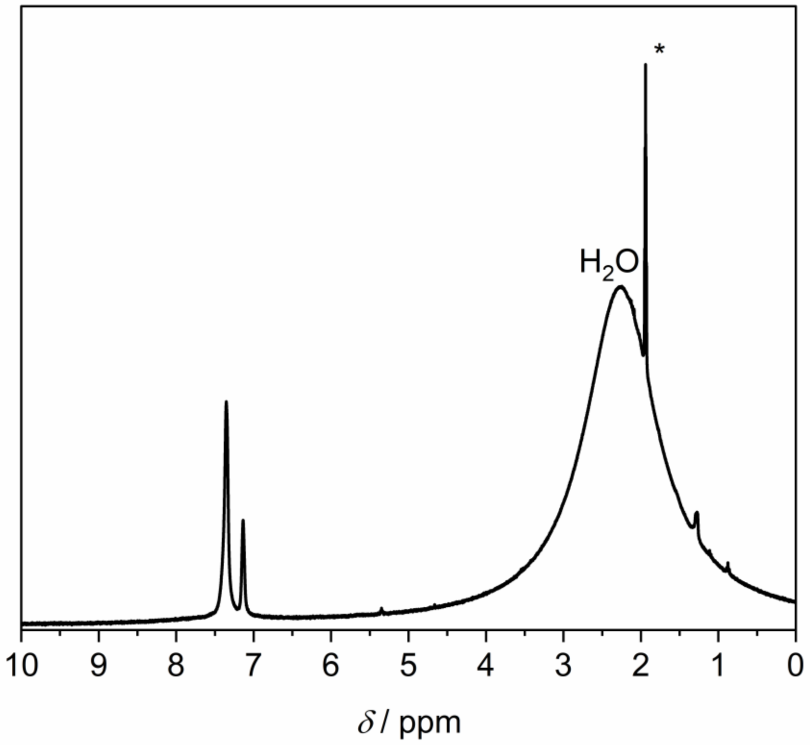

Figure S14 ^{1}H NMR spectrum (400 MHz, CD_3CN, 298 K) of the copper model complex [(2-phenylethylamine)$_4$CuCl$_2$] (refer to Chapter 4.6). * Residual solvent resonance.

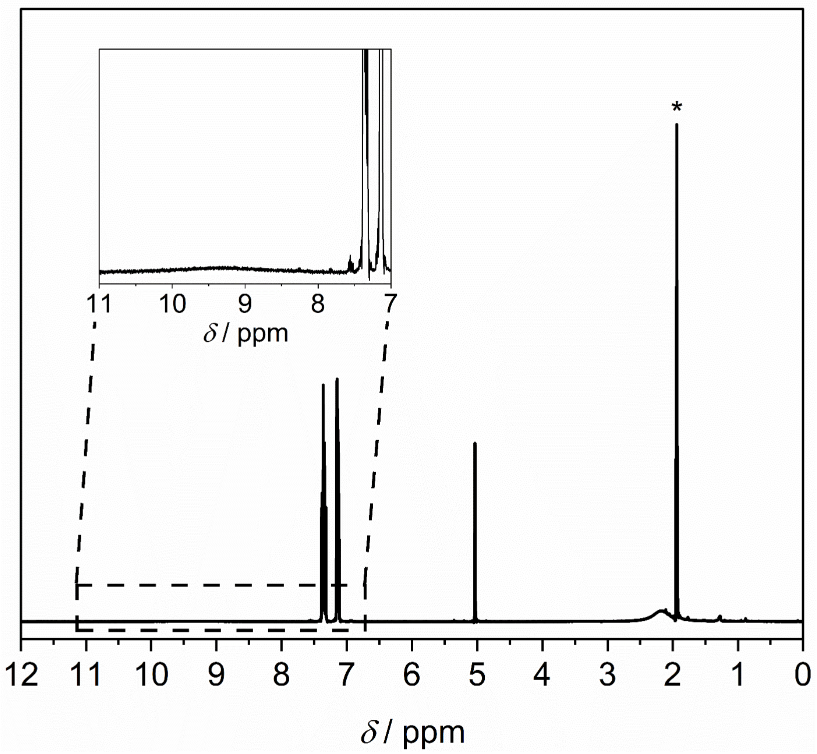

Figure S15 ^{1}H NMR spectrum (400 MHz, CD_3CN, 298 K) of xanthene-9-carboxylic acid. * Residual solvent resonance.

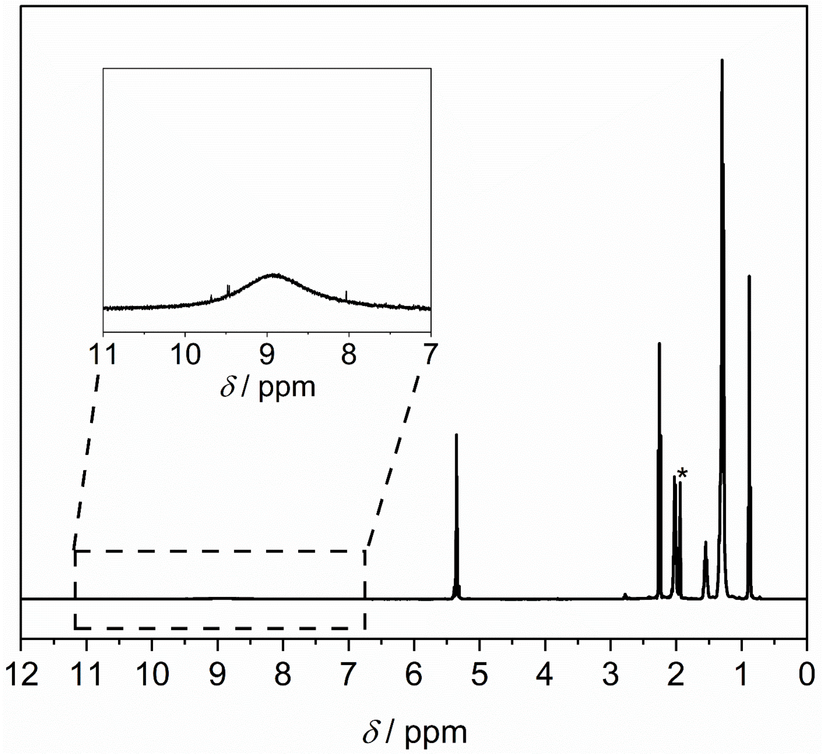

Figure S16 ^{1}H NMR spectrum (400 MHz, CD_3CN, 298 K) of oleic acid. * Residual solvent resonance.

5.2 Size Exclusion Chromatography (SEC) Data

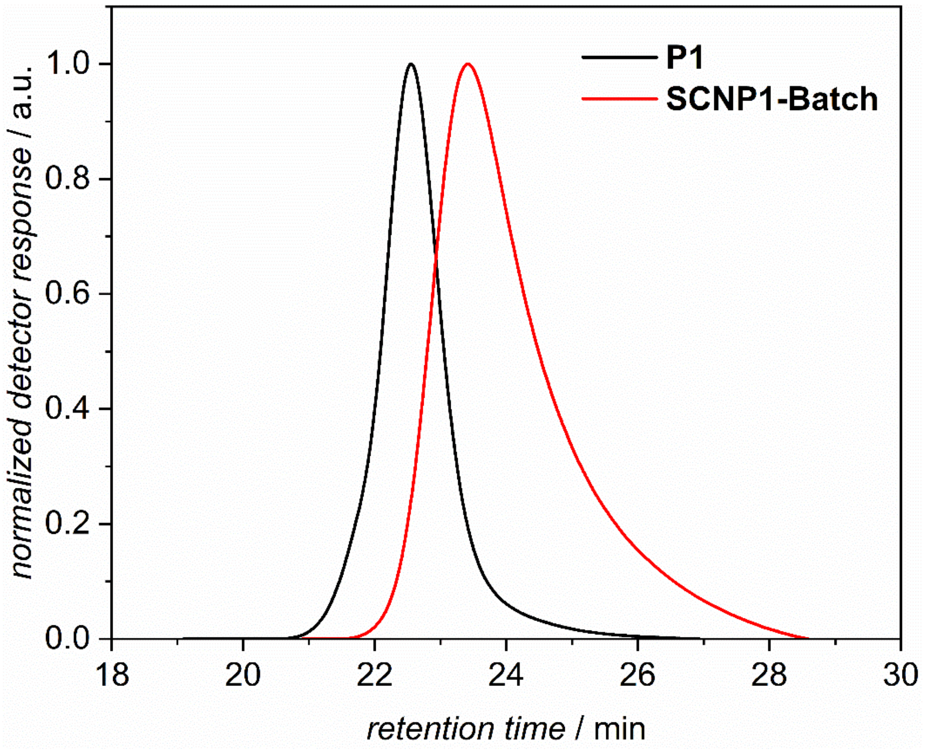

Figure S17 SEC chromatograms (THF, RI) of polymer **P1** (black) and **SCNP1-Batch** (red).

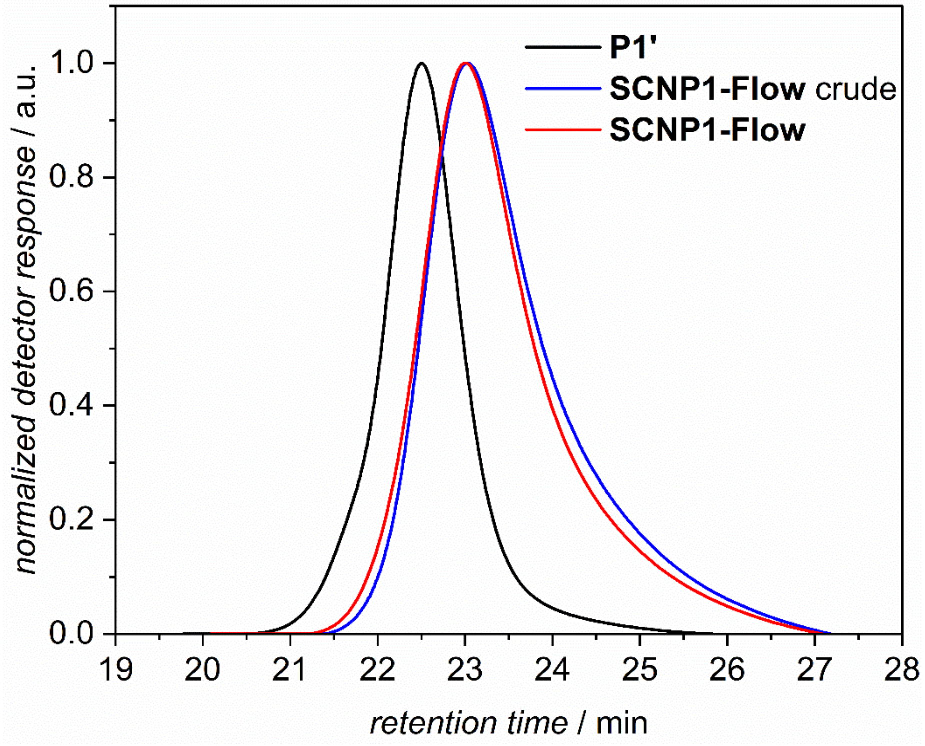

Figure S18 SEC chromatograms (THF, RI) of polymer **P1'** (black) and **SCNP1-Flow** before (crude, blue) and after (red) purification by preparative SEC.

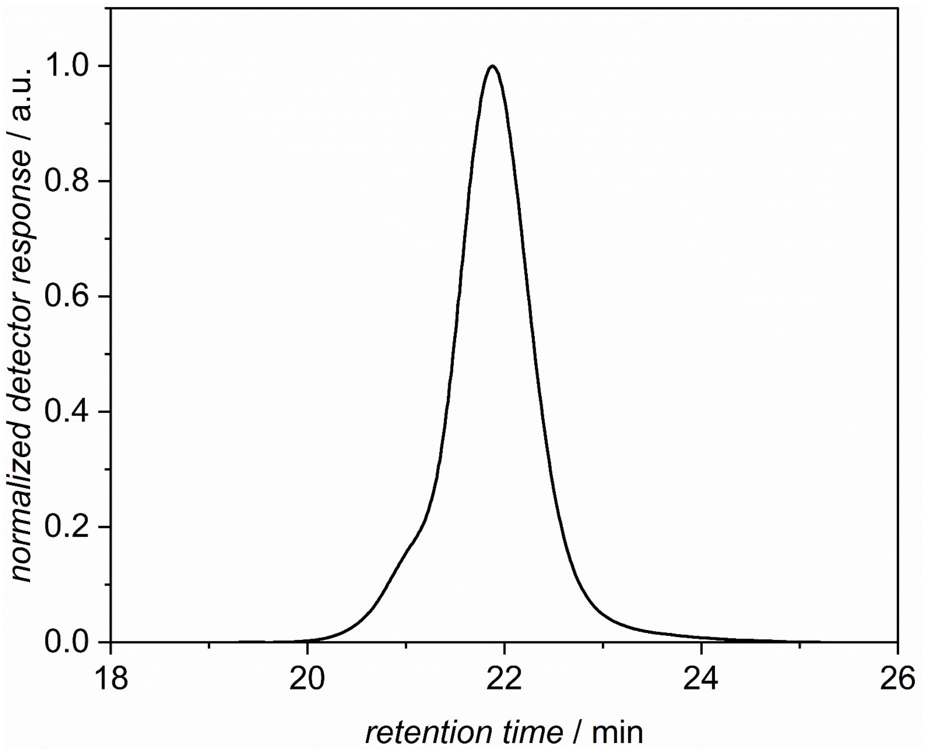

Figure S19 SEC chromatogram (THF, RI) of Poly(PEGMEMA).

5.3 Dynamic Light Scattering (DLS) Data

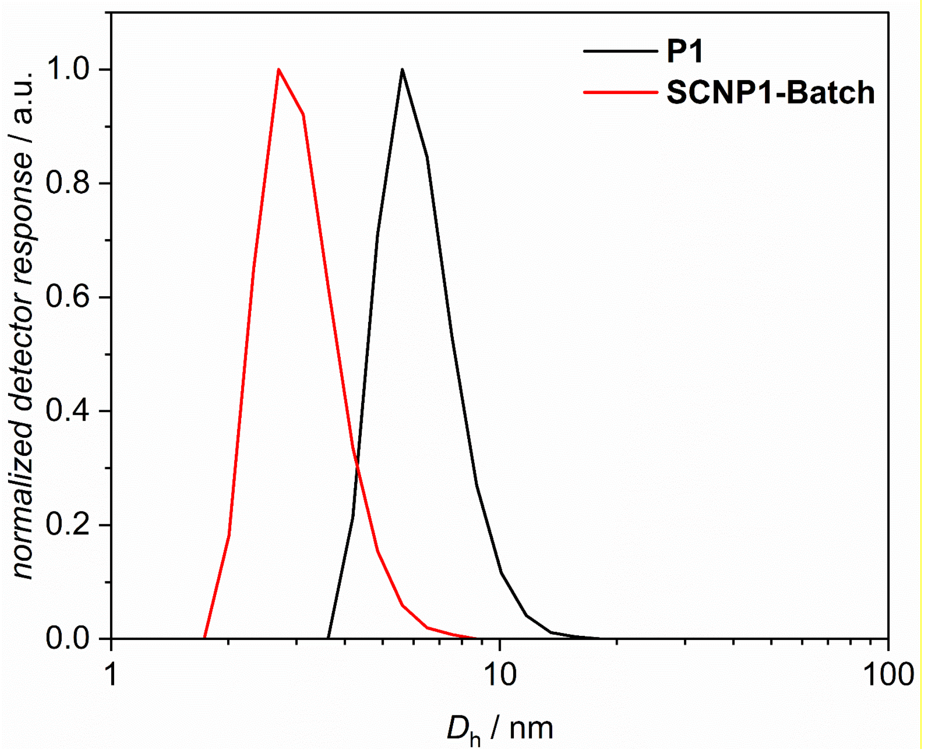

Figure S20 Number-averaged size distributions of **P1** (black) and **SCNP1-Batch** (red) obtained by DLS measurements in acetonitrile at 298 K.

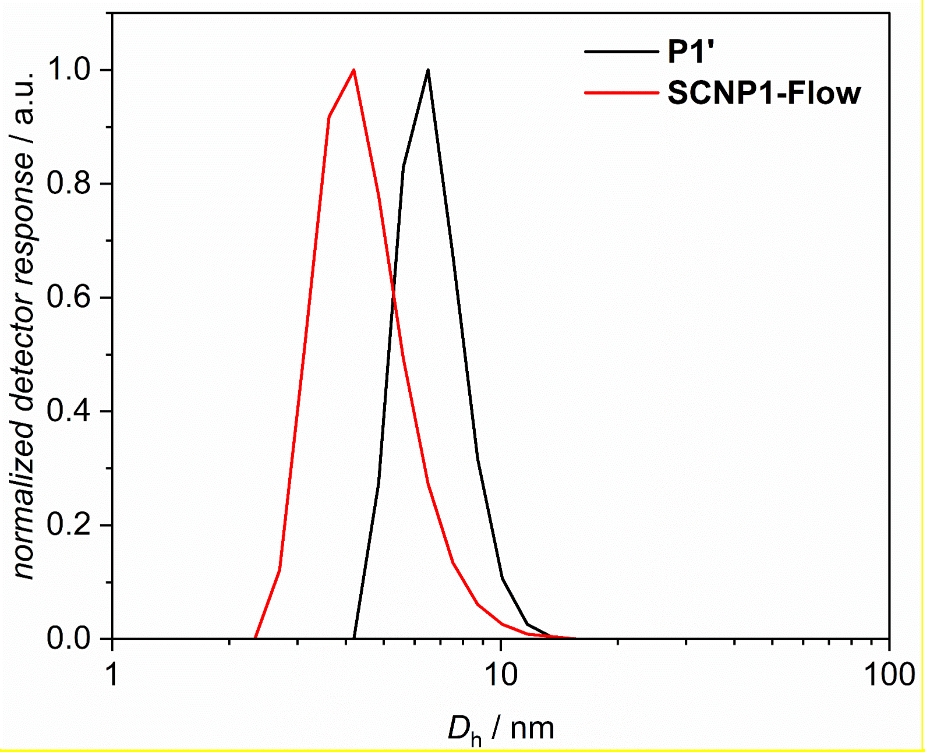

Figure S21 Number-averaged size distributions of **P1’** (black) and **SCNP1-Flow** (red) obtained by DLS measurements in acetonitrile at 298 K.

5.4 Diffusion-Ordered Nuclear Magnetic Resonance (DOSY) Spectra

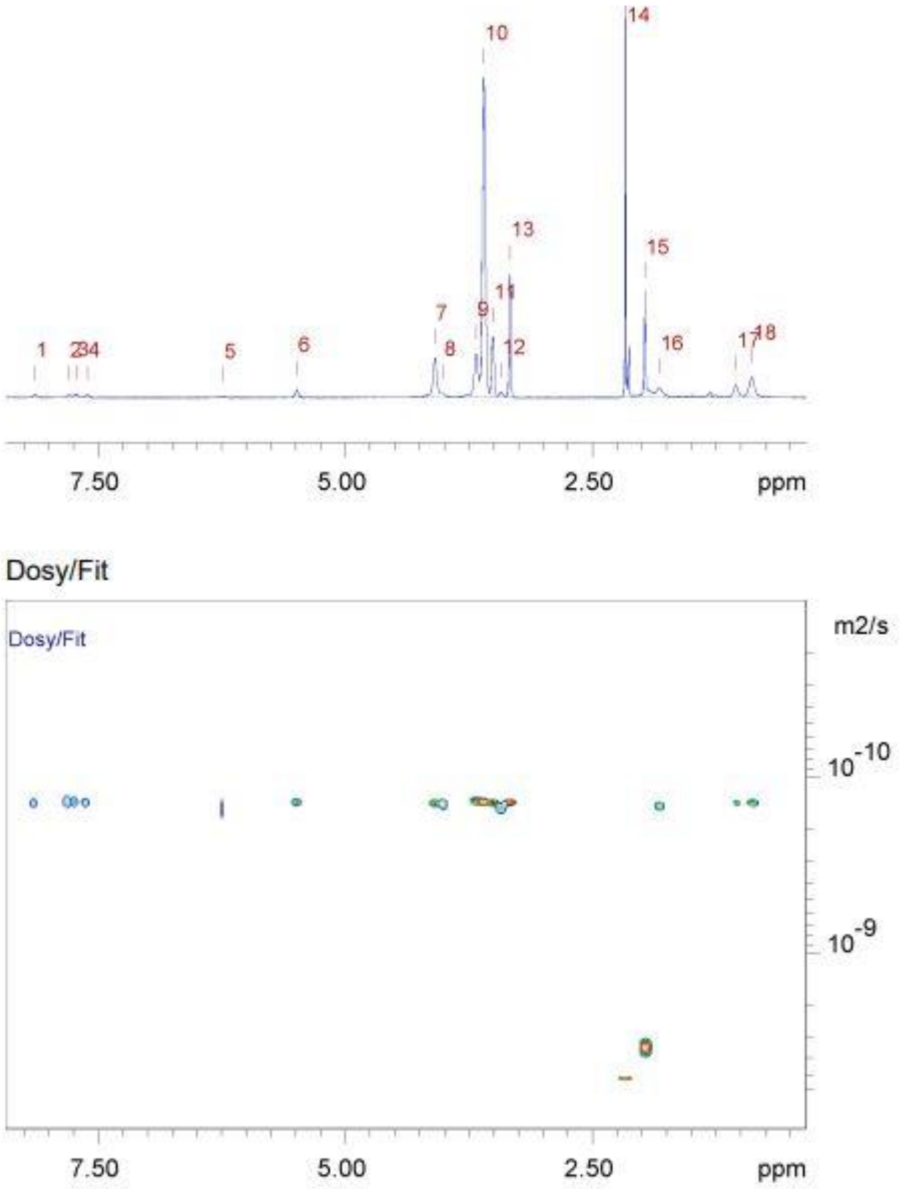

Figure S22 DOSY NMR spectrum (400 MHz, CD_3CN, 301 K) of polymer **P1**.

Table S5 Details of parameters and derived values of DOSY NMR measurements of polymer **P1**.

Fitted function:	f (x) = Io * exp (-D * x^2 * gamma^2 * littleDelta^2 (bigDelta-littleDelta/3)* 10^4
used gamma:	26752 rad/(s*Gauss)
used little delta:	0.0040000 s
used big delta:	0.099900 s
used gradient strength:	variable
Random error estimation of data:	RMS per spectrum (or trace/plane)
Systematic error estimation of data:	worst case per peak scenario
Fit parameter Error estimation method:	from fit using arbitrary y uncertainties
Confidence level:	95%
Used peaks:	
Used integrals:	peak intensities
Used Gradient strength:	all values (including replicates) used

Peak name	F2 [ppm]	Io	error	D [m2/s]	error	fitInfo
1	8.135	2.59e+06	5.344e+04	1.42e-10	6.787e-12	Done
2	7.789	2.71e+06	6.997e+04	1.39e-10	8.321e-12	Done
3	7.721	2.96e+06	6.216e+04	1.39e-10	6.749e-12	Done
4	7.607	2.42e+06	3.950e+04	1.40e-10	5.289e-12	Done
5	6.233	1.12e+06	7.180e+04	1.53e-10	2.266e-11	Done
6	5.485	7.27e+06	7.018e+04	1.41e-10	3.135e-12	Done
7	4.090	3.55e+07	2.050e+05	1.41e-10	1.884e-12	Done
8	4.008	3.61e+06	6.989e+04	1.44e-10	6.419e-12	Done
9	3.674	3.90e+07	2.734e+05	1.38e-10	2.240e-12	Done
10	3.596	2.88e+08	1.616e+06	1.39e-10	1.809e-12	Done
11	3.505	5.54e+07	2.902e+05	1.41e-10	1.706e-12	Done
12	3.421	5.06e+06	8.616e+04	1.52e-10	5.979e-12	Done
13	3.331	1.10e+08	5.943e+05	1.40e-10	1.752e-12	Done
14	2.161	3.75e+08	6.088e+05	5.15e-09	1.838e-11	Done
15	1.967	9.81e+07	2.093e+06	3.44e-09	1.630e-10	Done
16	1.823	8.77e+06	1.190e+05	1.47e-10	4.612e-12	Done
17	1.046	1.11e+07	1.020e+05	1.42e-10	3.033e-12	Done
18	0.885	1.81e+07	1.400e+05	1.42e-10	2.544e-12	Done

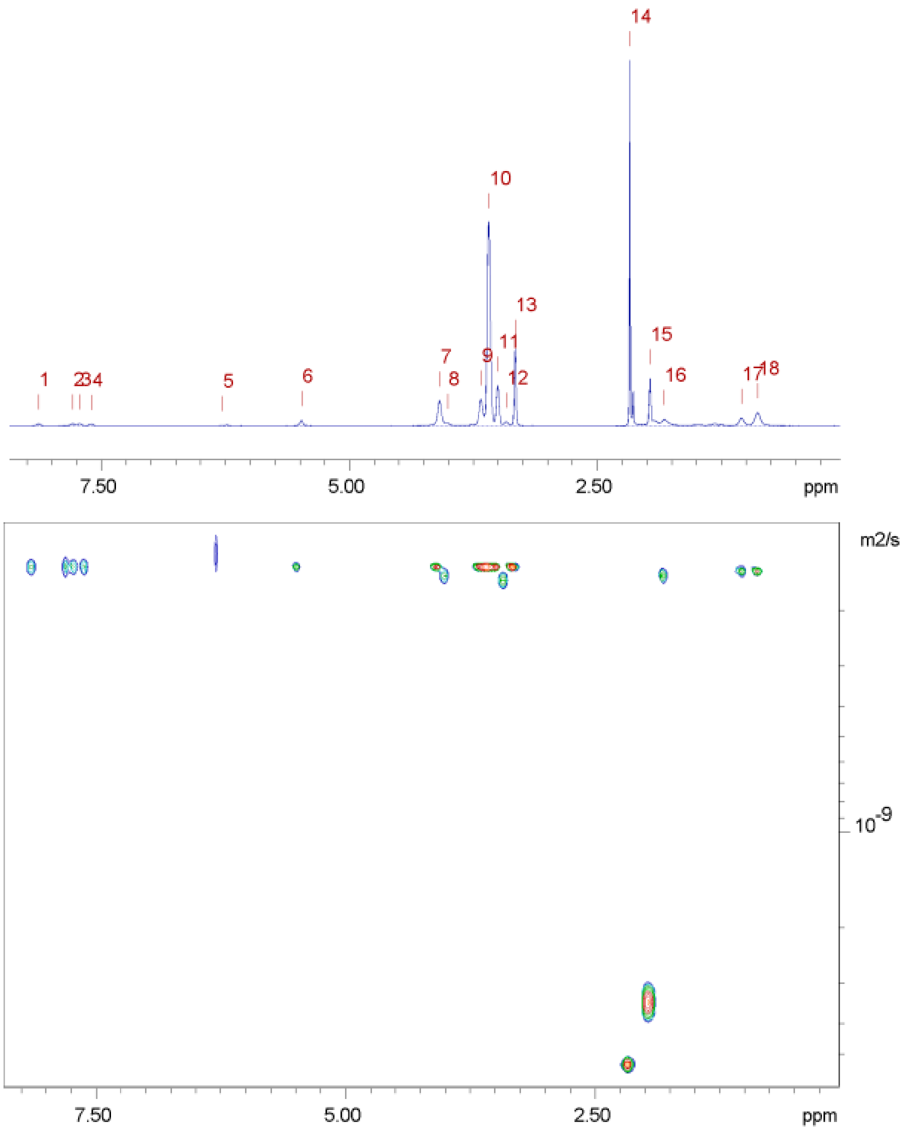

Figure S23 DOSY NMR spectrum (400 MHz, CD_3CN, 301 K) of polymer **P1’**.

Table S6 Details of parameters and derived values of DOSY NMR measurements of polymer **P1'**.

Fitted function:	f (x) = Io * exp (-D * x^2 * gamma^2 * littleDelta^2 (bigDelta-littleDelta/3)* 10^4
used gamma:	26752 rad/(s*Gauss)
used little delta:	0.0040000 s
used big delta:	0.099900 s
used gradient strength:	variable
Random error estimation of data:	RMS per spectrum (or trace/plane)
Systematic error estimation of data:	worst case per peak scenario
Fit parameter Error estimation method:	from fit using arbitrary y uncertainties
Confidence level:	95%
Used peaks:	
Used integrals:	peak intensities
Used Gradient strength:	all values (including replicates) used

Peak name	F2 [ppm]	Io	error	D [m2/s]	error	fitInfo
1	8.133	1.66e+06	3.197e+04	1.46e-10	6.455e-12	Done
2	7.789	1.74e+06	4.371e+04	1.47e-10	8.484e-12	Done
3	7.719	1.94e+06	3.286e+04	1.45e-10	5.665e-12	Done
4	7.605	1.60e+06	3.275e+04	1.48e-10	6.966e-12	Done
5	6.282	5.36e+05	3.832e+04	1.31e-10	2.175e-11	Done
6	5.484	4.76e+06	4.063e+04	1.46e-10	2.877e-12	Done
7	4.087	2.23e+07	9.912e+04	1.46e-10	1.498e-12	Done
8	4.007	2.35e+06	3.505e+04	1.54e-10	5.277e-12	Done
9	3.672	2.41e+07	9.202e+04	1.45e-10	1.274e-12	Done
10	3.592	1.82e+08	7.320e+05	1.46e-10	1.351e-12	Done
11	3.502	3.56e+07	1.348e+05	1.47e-10	1.284e-12	Done
12	3.420	3.46e+06	5.402e+04	1.60e-10	5.725e-12	Done
13	3.330	6.92e+07	2.320e+05	1.45e-10	1.121e-12	Done
14	2.175	6.76e+07	6.819e+05	5.44e-09	1.210e-10	Done
15	1.967	4.38e+07	1.249e+06	3.38e-09	2.149e-10	Done
16	1.825	5.58e+06	7.350e+04	1.54e-10	4.671e-12	Done
17	1.043	7.01e+06	6.170e+04	1.49e-10	3.015e-12	Done
18	0.884	1.16e+07	7.638e+04	1.49e-10	2.259e-12	Done

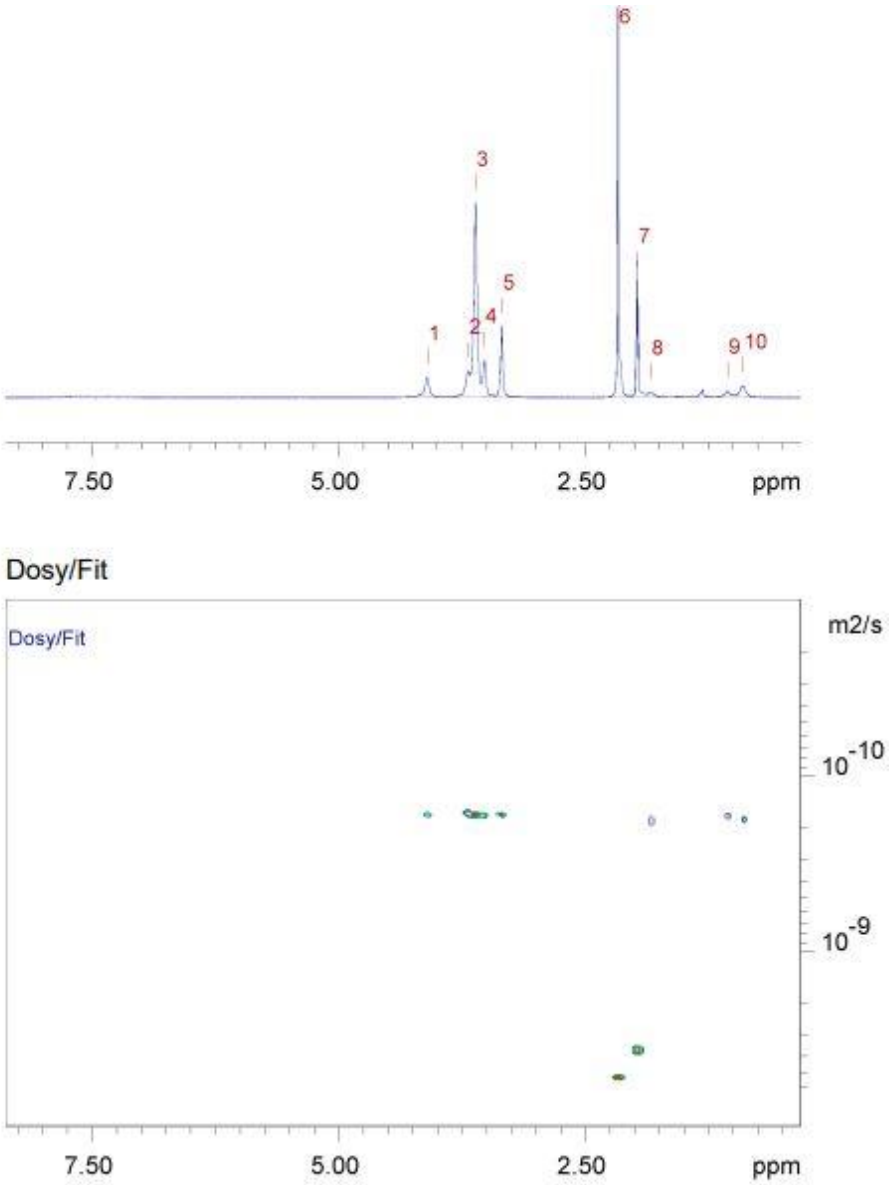

Figure S24 DOSY NMR spectrum (400 MHz, CD_3CN, 301 K) of **SCNP1-Batch**.

Table S7 Details of parameters and derived values of DOSY NMR measurements of **SCNP1-Batch**.

Fitted function:	f (x) = Io * exp (-D * x^2 * gamma^2 * littleDelta^2 (bigDelta-littleDelta/3)* 10^4
used gamma:	26752 rad/(s*Gauss)
used little delta:	0.0036000 s
used big delta:	0.099900 s
used gradient strength:	variable
Random error estimation of data:	RMS per spectrum (or trace/plane)
Systematic error estimation of data:	worst case per peak scenario
Fit parameter Error estimation method:	from fit using arbitrary y uncertainties
Confidence level:	95%
Used peaks:	
Used integrals:	peak intensities
Used Gradient strength:	all values (including replicates) used

Peak name	F2 [ppm]	Io	error	D [m2/s]	error	fitInfo
1	4.098	1.43e+07	8.125e+04	1.68e-10	2.214e-12	Done
2	3.685	1.79e+07	1.779e+05	1.65e-10	3.796e-12	Done
3	3.610	1.37e+08	5.852e+05	1.67e-10	1.645e-12	Done
4	3.517	2.63e+07	1.591e+05	1.69e-10	2.363e-12	Done
5	3.342	5.10e+07	1.994e+05	1.67e-10	1.510e-12	Done
6	2.163	2.91e+08	5.854e+05	5.22e-09	2.321e-11	Done
7	1.968	9.98e+07	1.117e+06	3.65e-09	9.085e-11	Done
8	1.833	3.59e+06	1.035e+05	1.83e-10	1.217e-11	Done
9	1.053	4.46e+06	6.497e+04	1.71e-10	5.753e-12	Done
10	0.894	8.13e+06	1.057e+05	1.79e-10	5.384e-12	Done

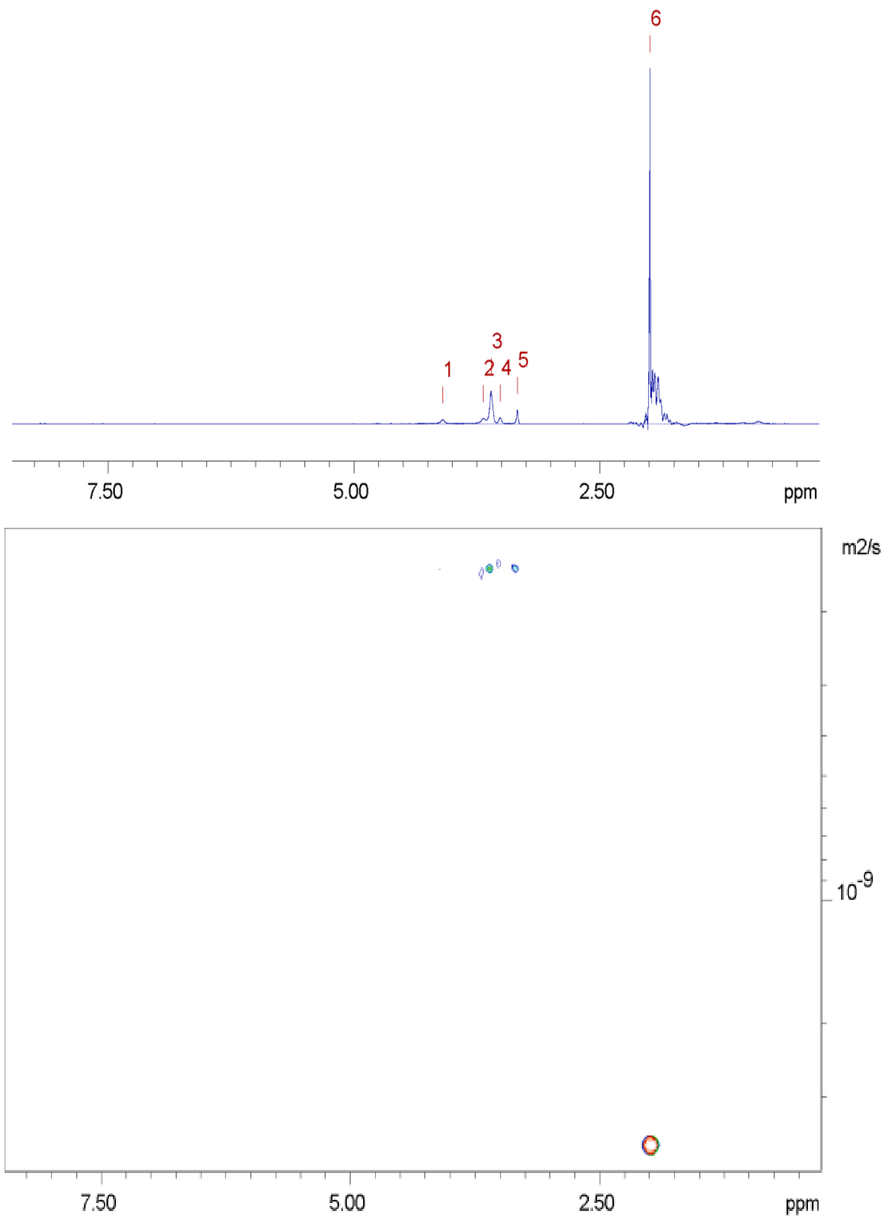

Figure S25 DOSY NMR spectrum (400 MHz, CH_3CN/CD_3CN, 301 K) of **SCNP1-Flow**.

Table S8 Details of parameters and derived values of DOSY NMR measurements of **SCNP1-Flow**.

Fitted function:	f (x) = Io * exp (-D * x^2 * gamma^2 * littleDelta^2 (bigDelta-littleDelta/3)* 10^4
used gamma:	26752 rad/(s*Gauss)
used little delta:	0.0036000 s
used big delta:	0.099900 s
used gradient strength:	variable
Random error estimation of data:	RMS per spectrum (or trace/plane)
Systematic error estimation of data:	worst case per peak scenario
Fit parameter Error estimation method:	from fit using arbitrary y uncertainties
Confidence level:	95%
Used peaks:	
Used integrals:	peak intensities
Used Gradient strength:	all values (including replicates) used

Peak name	F2 [ppm]	Io	error	D [m2/s]	error	fitInfo
1	4.100	1.86e+04	267.7	1.56e-10	5.228e-12	Done
2	3.688	2.50e+04	455.9	1.60e-10	6.728e-12	Done
3	3.608	1.54e+05	788.2	1.55e-10	1.838e-12	Done
4	3.515	2.94e+04	343.5	1.54e-10	4.180e-12	Done
5	3.339	6.51e+04	372.1	1.55e-10	2.058e-12	Done
6	1.991	4.83e+08	2.159e+06	3.89e-09	3.879e-11	Done

5.5 Energy Dispersive X-Ray Spectroscopy (EDX) of SCNP1-Flow

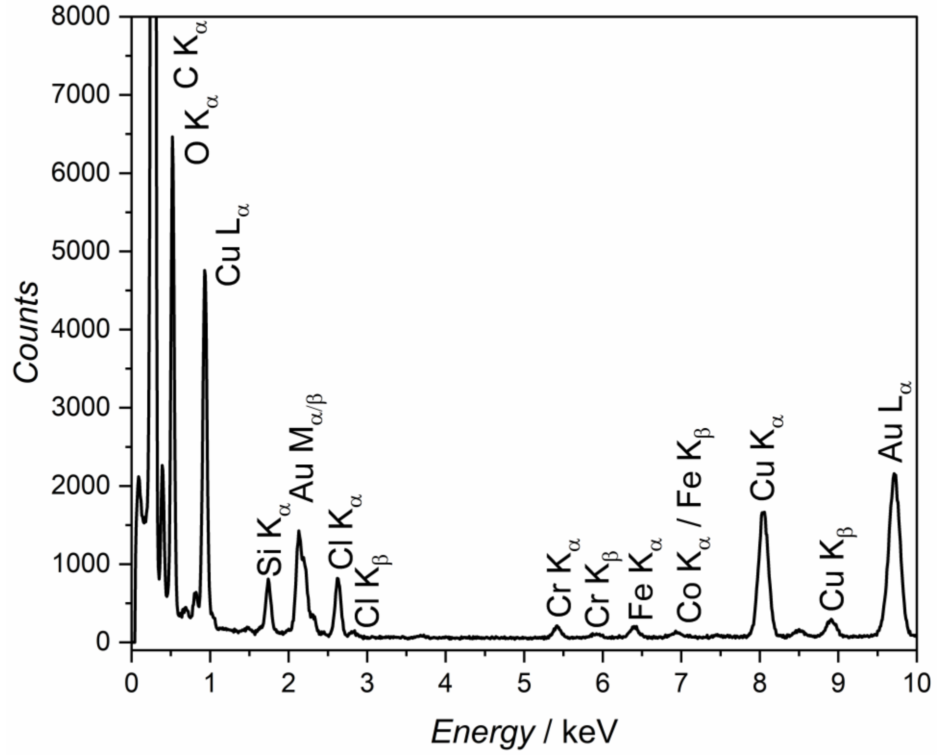

Figure S26 EDX spectrum of **SCNP1-Flow**. Most intense peaks are labelled with the symbols of the elements they are characteristic for. Additional peaks not corresponding to the polymer sample result from Si (detector), Cr, Fe, Co (TEM background) and Au (TEM grid).

5.6 UV/Vis of 2-((((2-nitrobenzyl)oxy)carbonyl)amino)ethyl methacrylate and LED Emission Spectra

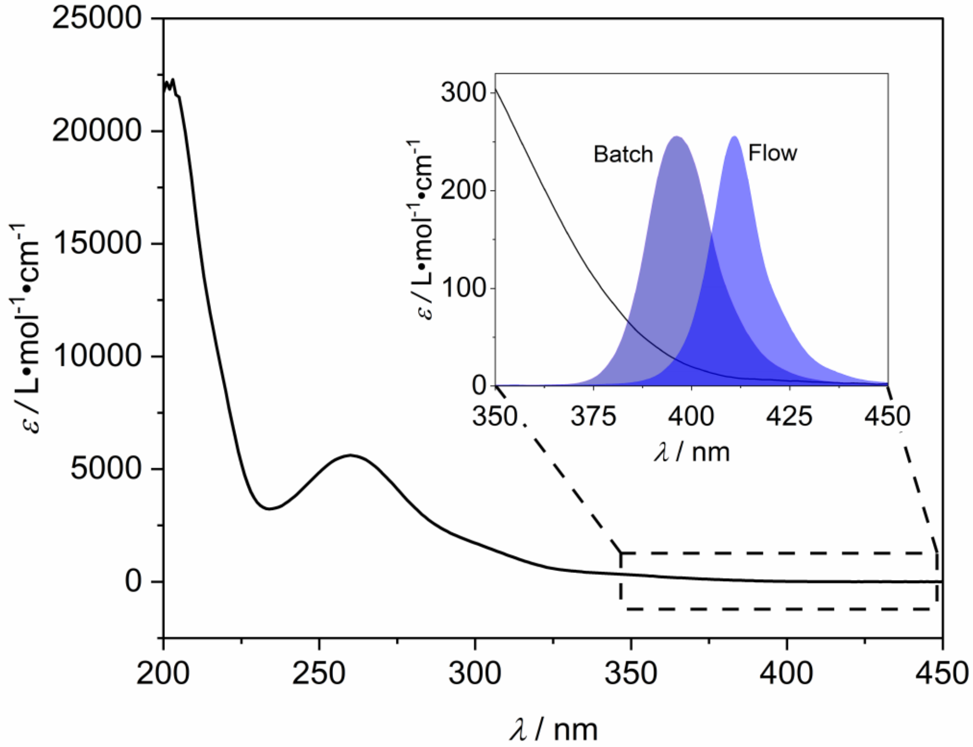

Figure S27 UV/Vis spectrum of 2-((((2-nitrobenzyl)oxy)carbonyl)amino)ethyl methacrylate in acetonitrile at 298 K and emission spectra of the LEDs employed for the batch (400 nm, 10 W, labelled with "Batch") and flow (410 nm, 12 W, labelled with "Flow") synthesis of single-chain nanoparticles described in this work. LED emission spectra were recorded on an Ocean Optics Miniature Spectrometer FLAME-T-UV-VIS.

5.7 Single-Crystal X-Ray Diffraction (XRD) Data

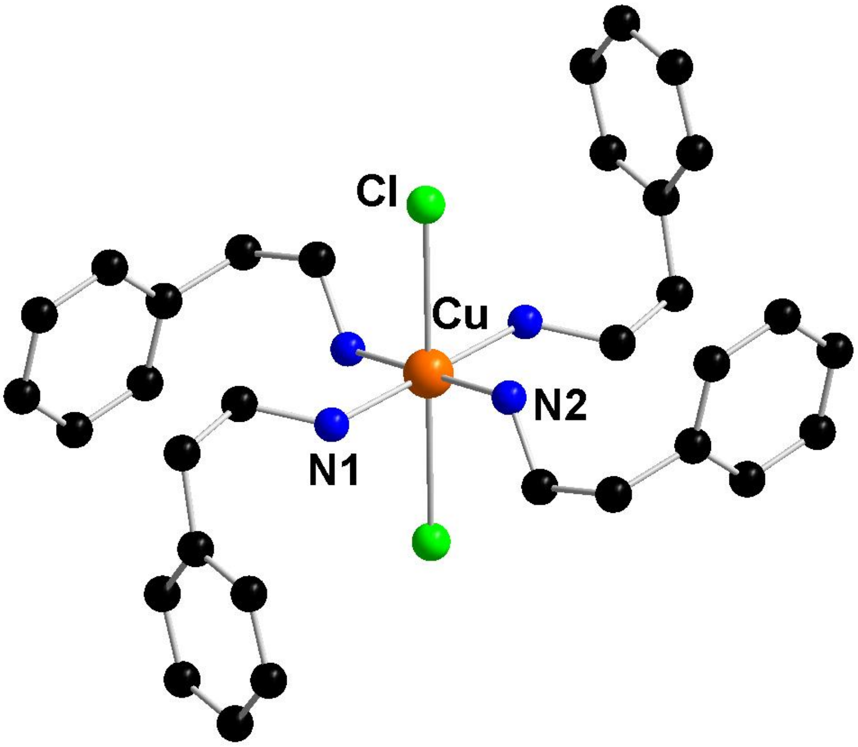

Figure S28 Solid-state molecular structure of [(2-phenylethylamine)$_4$CuCl$_2$]. Selected bond distances [Å] and angles [°]: Cu-N1 2.033(2), Cu-N2 2.044(2), Cu-Cl 2.8080(7), N1-Cu-N2 87.01(9), N1-Cu-Cl 98.04(7), N2-Cu-Cl 83.46(7). Hydrogen atoms and non-coordinating solvent molecules are omitted for clarity.

Table S9 Summary of crystal data and structure refinement of [(2-phenylethylamine)$_4$CuCl$_2$].

Identification code	SG114
Empirical formula	$C_{36}H_{50}Cl_2CuN_6$
Formula weight	701.26
Temperature/K	100
Crystal system	monoclinic
Space group	$P2_1/n$
a/Å	14.4097(15)
b/Å	5.8858(7)
c/Å	21.876(3)
α/°	90
β/°	92.905(9)
γ/°	90
Volume/Å^3	1853.0(4)
Z	2
ρ_{calc}/g·cm^{-3}	1.257
μ/mm^{-1}	0.766
F(000)	742.0
Crystal size/mm^3	0.231 × 0.106 × 0.043
Radiation	Mo-K_α (λ = 0.71073)
2Θ range for data collection/°	3.728 to 60.468
Index ranges	$-19 \le h \le 16$, $-8 \le k \le 7$, $-27 \le l \le 29$
Reflections collected	11145
Independent reflections	4550 [R_{int} = 0.0477, R_{sigma} = 0.0718]
Data/restraints/parameters	4550/0/206
Goodness-of-fit on F^2	1.032
Final R indexes [I>=2σ (I)]	R_1 = 0.0511, wR_2 = 0.1100
Final R indexes [all data]	R_1 = 0.0976, wR_2 = 0.1297
Largest diff. peak/hole / e Å^{-3}	0.53/-0.91

5.8 Infrared Spectroscopy (IR) Data

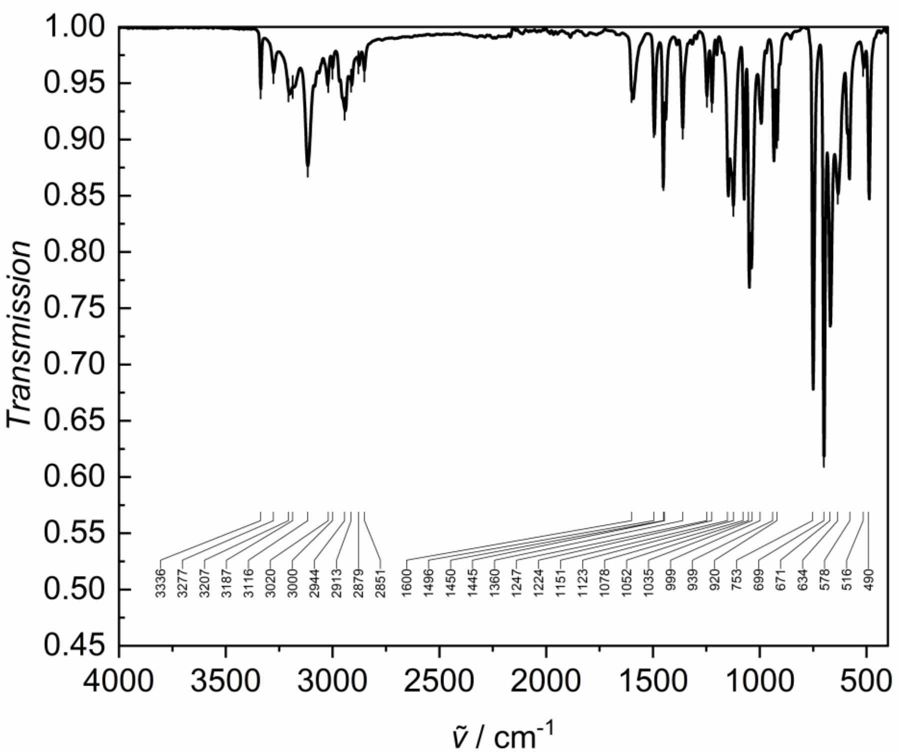

Figure S29 FT-IR (ATR) spectrum of the copper model complex [(2-phenylethylamine)$_4$CuCl$_2$] (refer to Chapter 4.6).

5.9 Electron Paramagnetic Resonance (EPR) Data

The paramagnetic nature of the d^9 Cu(II) ions present in **SCNP1-Flow** allows for the quantification of the amount of Cu(II) incorporated into the SCNPs via EPR spectroscopy. Therefore, a calibration curve based on varying concentrations of $CuCl_2$·2 H_2O in acetonitrile as reference was recorded (refer to **Figure S30**). The concentrations and resulting double integrals of the EPR signal intensity are listed in **Table S10**. Following the general synthetic procedure (refer to Chapter 4.5), three batches of **SCNP1-Flow** were prepared and their EPR spectra recorded under conditions and instrument settings identical to the calibration samples. The determined double integrals of the EPR signal intensities as well as the derived Cu(II) concentrations are collated in **Table S10**.

Figure S31 depicts the superimposition of all spectra. **Figure S32** shows the stacked spectra of the highest and lowest concentration calibration samples, respectively, as well as the spectra of all three **SCNP1-Flow** samples. All depicted spectra are baseline corrected with the spectrum of an empty tube. We note that the baseline signal is about one third of the intensity of the lowest concentration calibration sample, leading to slight distortions of the spectra.

Table S10 Summary of EPR results and derived data for the $CuCl_2$·2 H_2O calibration samples as well as the three samples of **SCNP1-Flow**. The given Cu(II) concentrations for **SCNP1-Flow** were derived from the linear fit curve of the calibration data shown in **Figure S30** based on the measured double integrals of the EPR signal intensity.

	Double integral of EPR signal intensity / a.u	Cu(II) concentration / mmol·L^{-1}	Respective $CuCl_2$·2 H_2O concentration / mg·mL^{-1}
$CuCl_2$·2 H_2O – Calibration 1	231.74	0.0587	0.01
$CuCl_2$·2 H_2O – Calibration 2	629.86	0.117	0.02
$CuCl_2$·2 H_2O – Calibration 3	2636.55	0.293	0.05
$CuCl_2$·2 H_2O – Calibration 4	4951.72	0.587	0.1
**SCNP1-Flow** – Sample 1	101.98	0.0431	0.0074
**SCNP1-Flow** – Sample 2	236.94	0.0579	0.0099
**SCNP1-Flow** – Sample 3	197.97	0.0537	0.0091

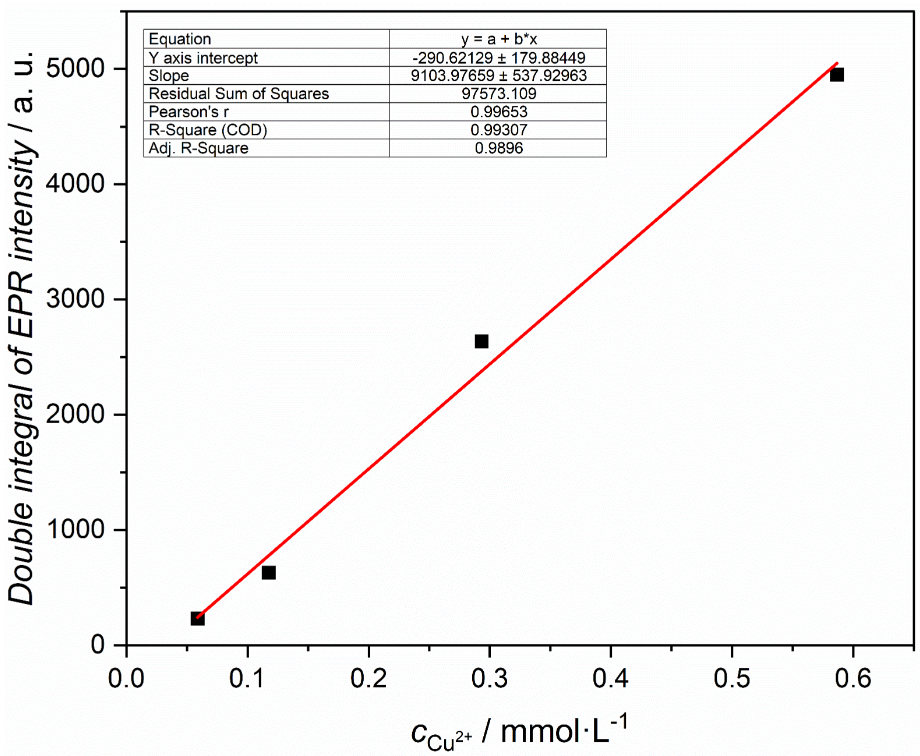

Figure S30 Calibration curve (red) and data points (black) showing the double integral of the EPR signal intensity (X-band CW EPR, CH_3CN, 115 K) in dependence on the Cu(II) concentration. $CuCl_2 \cdot 2\ H_2O$ was used as calibration standard. Red line represents linear fit on data points obtained with OriginPro 2023.

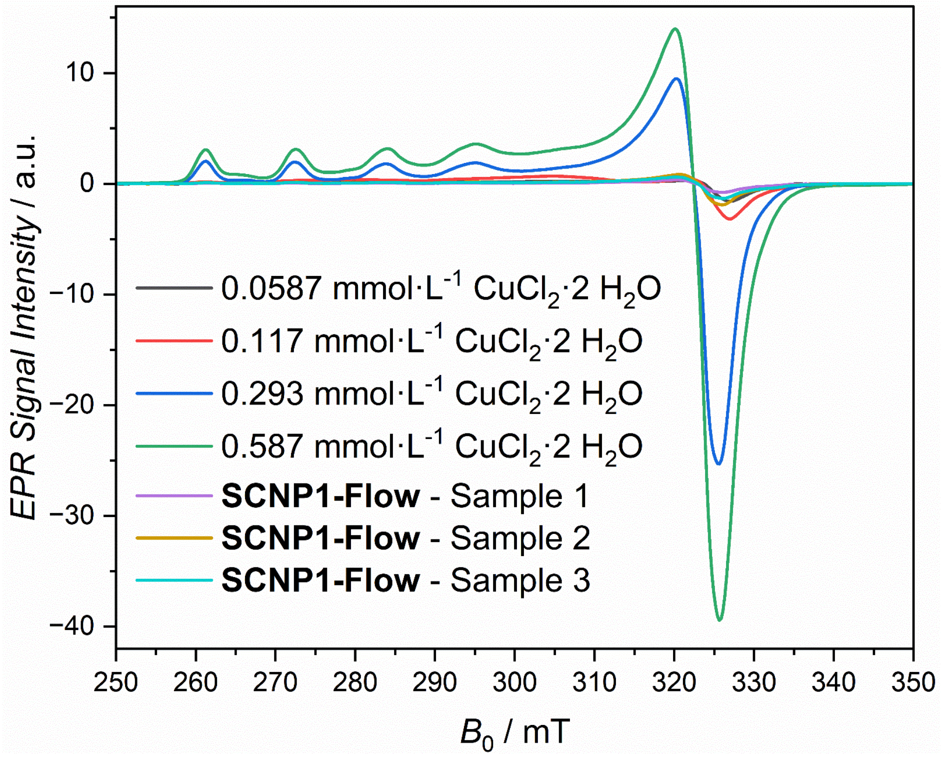

Figure S31 Superimposed X-band CW EPR spectra (CH_3CN, 115 K) of different concentration $CuCl_2 \cdot 2\ H_2O$ samples as well as three different batches of **SCNP1-Flow**.

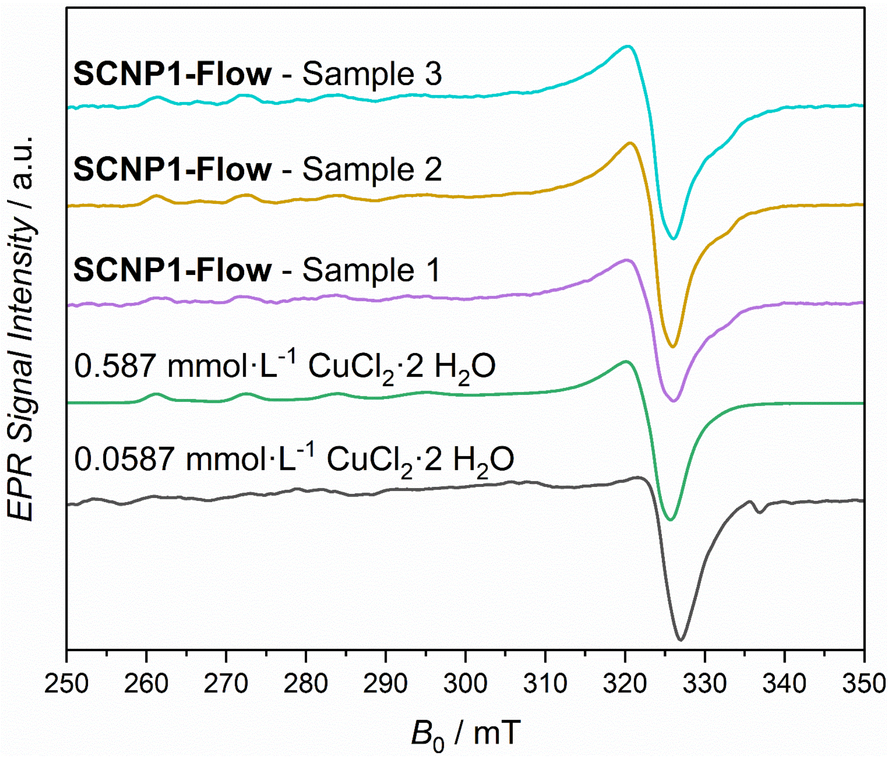

Figure S32 Stacked X-band CW EPR spectra (CH_3CN, 115 K) of the lowest and highest concentration $CuCl_2$·2 H_2O calibration samples as well as the three samples of **SCNP1-Flow** showing their similar shape. Spectra were scaled to have similar intensities.

5.10 Comparison of the Compaction of SCNP1-Batch and SCNP1-Flow

To compare the compaction achieved in the folding reaction via the batch and flow process, the synthesis of **SCNP1-Batch** and **SCNP1-Flow** was conducted from the same batch of precursor polymer (**P1'**) and the crude reaction mixture was purified by preparative SEC (Sephadex LH-20) in acetonitrile. The results of the size analysis by SEC, DLS and DOSY are summarized in **Table S11**.

According to SEC in *N,N*-dimethylacetamide (DMAc), the apparent peak molar mass (M_p) decreases from 36,200 g·mol^{-1} for **P1'** to 29,000 g·mol^{-1} for **SCNP1-Batch** and 31,900 g·mol^{-1} for **SCNP1-Flow**. DLS in acetonitrile shows a decrease in the number-averaged solvodynamic diameter from 6.66 nm for **P1'** to 2.40 nm for **SCNP1-Batch** and 4.65 nm for **SCNP1-Flow**, respectively. DOSY measurements in (deuterated) acetonitrile indicate an increase in the average diffusion coefficient from 1.47·10^{-10} m^2·s^{-1} for **P1'** to 1.78·10^{-10} m^2·s^{-1} for **SCNP1-Batch** and 1.56·10^{-10} m^2·s^{-1} for **SCNP1-Flow**.

In conclusion, the data clearly evidences a decrease in the size of the precursor polymer upon SCNP compaction. It is suggested by the data that the observed compaction is more pronounced for **SCNP1-Batch** than for **SCNP1-Flow**. It is possible that this is a result of uncontrolled side reactions occurring in the batch process which do not seem to occur in the flow process under the optimized conditions employed for the synthesis of **SCNP1-Flow**. This hypothesis is supported by the screening of flow rate and polymer concentration for the flow synthesis in which increasing the flow rate or concentration leads to more pronounced side reactions, accompanied by a larger apparent compaction (refer to SI **Figure S6** and **S8**). However, a detailed understanding of this observation would require in-depth knowledge of the processes occurring within the macromolecules on a molecular level which, at the present stage, is not achievable using state-of-the-art analytical methods.

Additionally, we refrain from undertaking any detailed quantitative discussion of the data for the following reasons: (*i*) Our THF SEC columns have been replaced since the data contained in the manuscript and SI has been recorded. The SCNP samples show pronounced enthalpic interactions with our current THF SEC columns and the same cannot be excluded for the old columns used during data acquisition for the current work. Further, the integrity of the Cu coordination under the harsh conditions of our DMAC SEC (60°C, LiBr) is highly questionable. (*ii*) The size range of our SCNPs (<5 nm) is at the lower detection limit of DLS, due to the weak scattering abilities of small particles, and should thus inherently be treated with caution. (*iii*) The pulse sequence used for the DOSY experiments (*ledbpgp2s*) does not compensate for effects of convection which are expected to be non-negligible under the measurement conditions. Thus, the size information derived from DOSY measurements is also to be treated with caution.

In summary, the data comparing the relative sizes of folded and unfolded polymers to follow the SCNP compaction process is to be considered qualitative in nature. Critically, making quantitative comparisons between the batch and flow SCNPs would not be scientifically sound when taking into account that the errors in the measured sizes upon repeated synthesis and measurements are of a similar magnitude as the differences between the sizes of **SCNP1-Batch** and **SCNP1-Flow**. We highlight that we have discussed the limitations of the methods commonly employed to follow SCNP compaction in a published article.[6]

Table S11 Summary of size analysis of **SCNP1-Batch** and **SCNP1-Flow**, both prepared from the same precursor polymer **P1'**.

	**P1'**	**SCNP1-Batch**	**SCNP1-Flow**
M_p / g·mol^{-1} (SEC, DMAc, RI, PMMA cal.)[a]	36,200	29,000	31,900
D_h / nm (DLS, CH_3CN)	6.66	2.40	4.65
D / m^2·s^{-1} (DOSY, CH_3CN/CD_3CN)[b]	$1.47 \cdot 10^{-10}$	$1.78 \cdot 10^{-10}$	$1.56 \cdot 10^{-10}$

[a] The column set of our THF SEC setup was replaced since the THF SEC data shown in the manuscript and SI was acquired. The new column set shows pronounced enthalpic interactions with the SCNP samples. Therefore, SEC data provided here was acquired in DMAc.

[b] **SCNP1-Batch** and **SCNP1-Flow** are obtained as solutions in acetonitrile after synthesis. Deuterated acetonitrile was added for DOSY analysis.

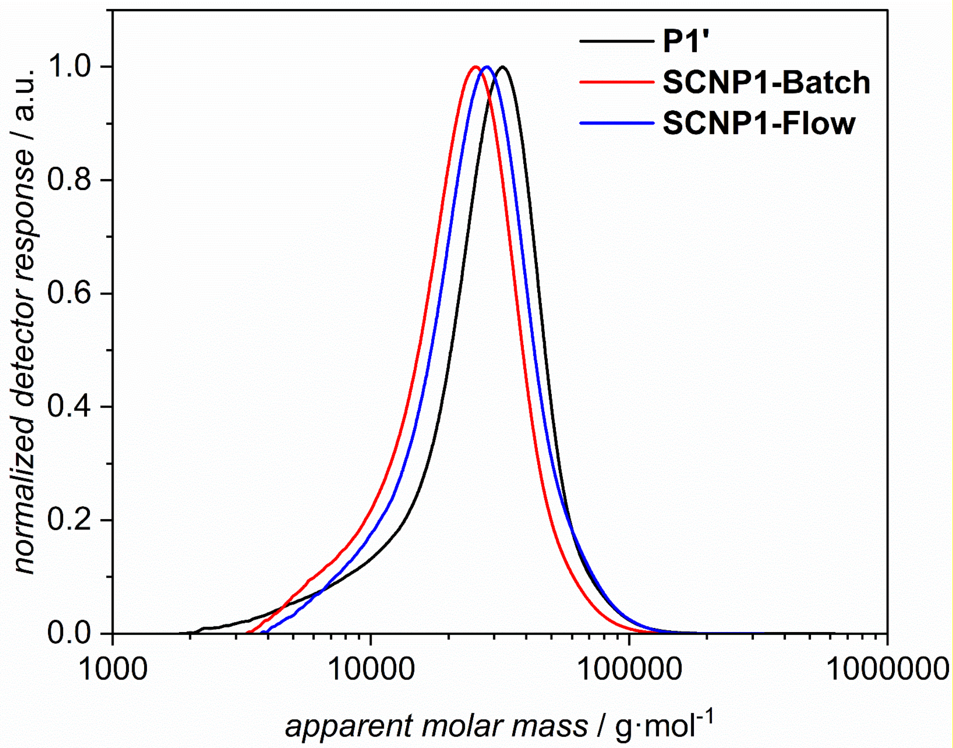

Figure S33 Number-averaged apparent molar mass distributions of polymer **P1'** (black), **SCNP1-Batch** (red) and **SCNP1-Flow** (blue) obtained by SEC (DMAc, RI, PMMA cal.).

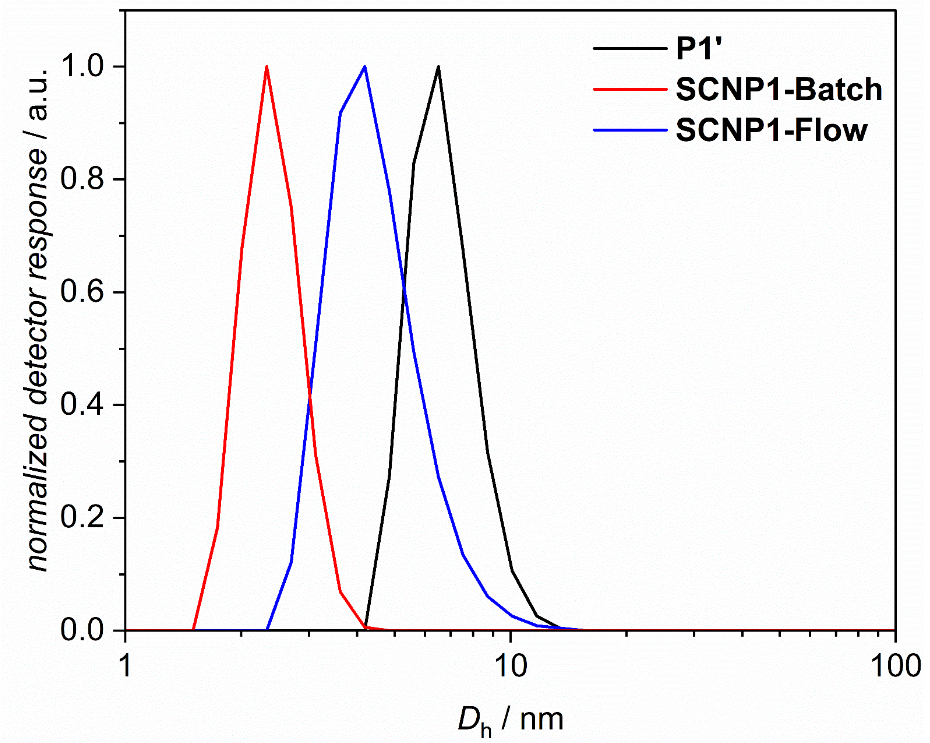

Figure S34 Number-averaged size distributions of **P1’** (black), **SCNP1-Batch** (red) and **SCNP1-Flow** (blue) obtained by DLS measurements in acetonitrile at 298 K.

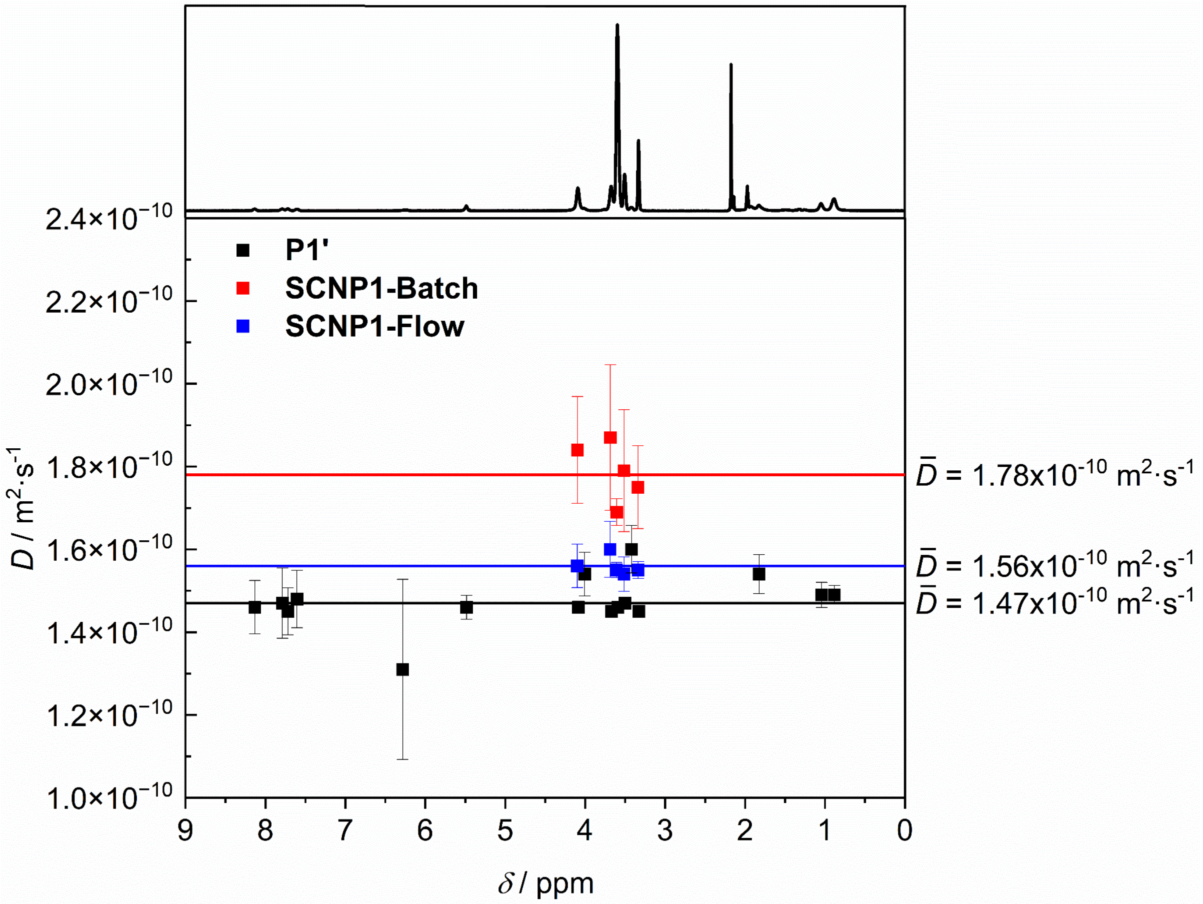

Figure S35 Superimposed DOSY NMR spectra (400 MHz, CH_3CN/CD_3CN, 301 K) of **P1’** (black), **SCNP1-Batch** (red) and **SCNP1-Flow** (blue).

6 Catalysis

6.1 Decarboxylation-Oxygenation of Xanthene-9-carboxylic acid

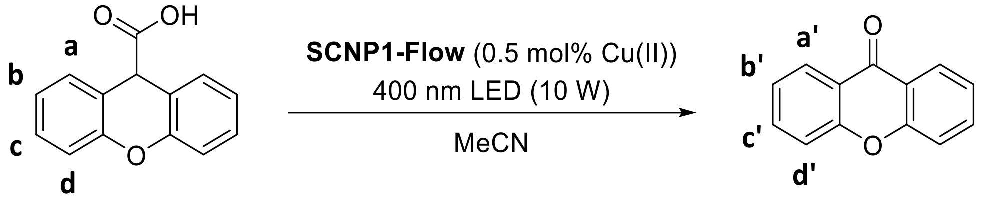

Scheme S1 Reaction scheme for the Cu(II) photocatalyzed decarboxylation-oxygenation of xanthene-9-carboxylic acid. Labels refer to **Figure S36**.

For reactions employing **SCNP1-Flow** as the catalyst, 10.0 mg of polymer **P1'** (4.98 µmol, 0.10 eq. photoreactive unit) and 0.84 mg $CuCl_2 \cdot 2\ H_2O$ (4.98 µmol, 0.10 eq.) were dissolved in 5 mL of acetonitrile. The resulting yellow solution was sparged with argon and subsequently irradiated using a 410 nm LED (12 W, refer to **Figure S27** for the emission spectrum) under photoflow conditions (flowrate 0.2 mL·min^{-1}, refer to Chapter 2.7 for details regarding the photoflow reactor). The obtained yellow solution was directly loaded onto a preparative SEC column (Sephadex LH-20) and eluted with acetonitrile, resulting in 5 mL of a pale-yellow solution of **SCNP1-Flow** (Cu(II) concentration about 0.05 mmol·L^{-1}, refer to Chapter 5.9). The solution was transferred into a 20 mL crimp vial and 11.3 mg xanthene-9-carboxylic acid (49.8 µmol, 1.00 eq.) were added. After sparging with oxygen, an oxygen-filled balloon was fitted on top of the vial and the solution was irradiated using a 400 nm LED (10 W, refer to **Figure S27** for the emission spectrum). 0.1 mL of the reaction solution were taken out before irradiation (t_0) and after 30, 60, 90 and 120 minutes, respectively, and 0.3 mL of CD_3CN added for NMR analysis. The reaction was performed in triplicate (refer to **Figure S36** to **S38**).

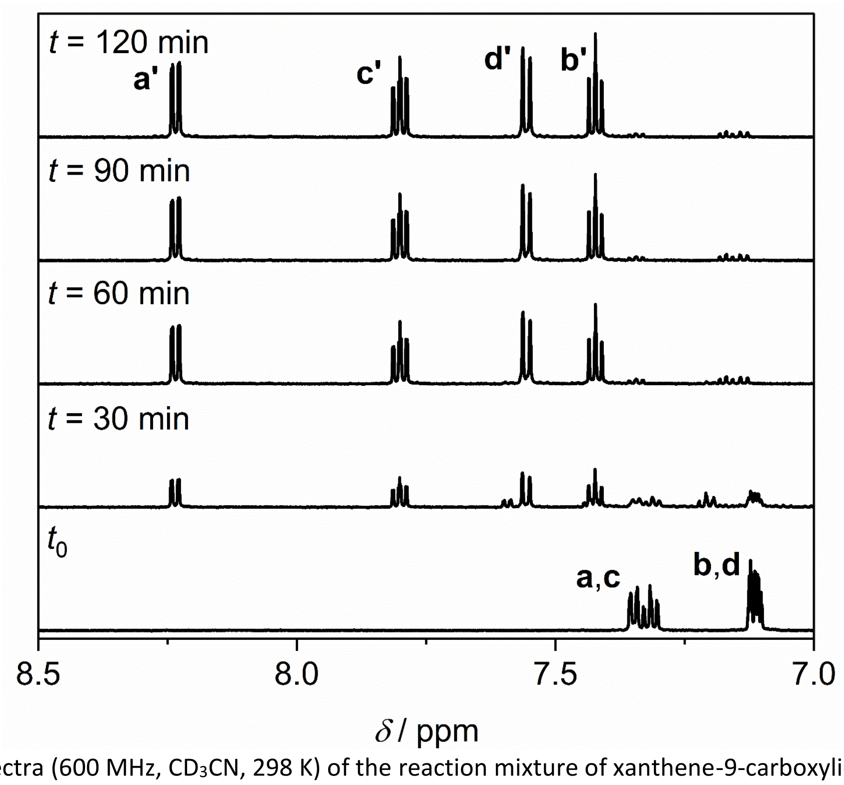

Figure S36 ^1H NMR spectra (600 MHz, CD_3CN, 298 K) of the reaction mixture of xanthene-9-carboxylic acid and **SCNP1-Flow** before (t_0) and at different times after starting the irradiation with a 400 nm LED (10 W, refer to **Figure S27** for the emission spectrum) under oxygen atmosphere. Labels refer to **Scheme S1**. Experiment 1.

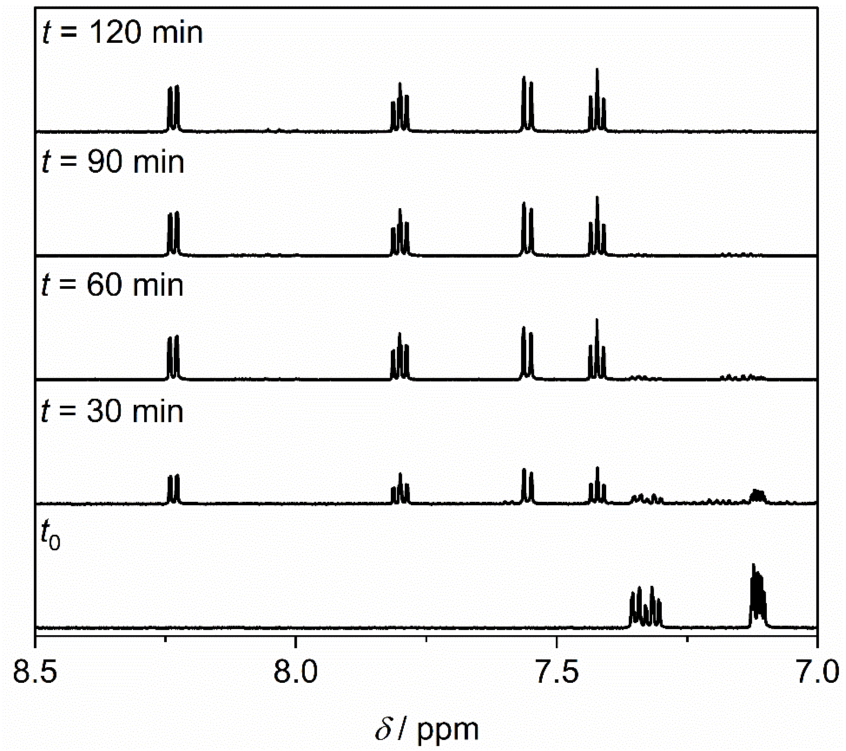

Figure S37 ^{1}H NMR spectra (600 MHz, CD_3CN, 298 K) of the reaction mixture of xanthene-9-carboxylic acid and **SCNP1-Flow** before (t_0) and at different times after starting the irradiation with a 400 nm LED (10 W, refer to **Figure S27** for the emission spectrum) under oxygen atmosphere. Experiment 2.

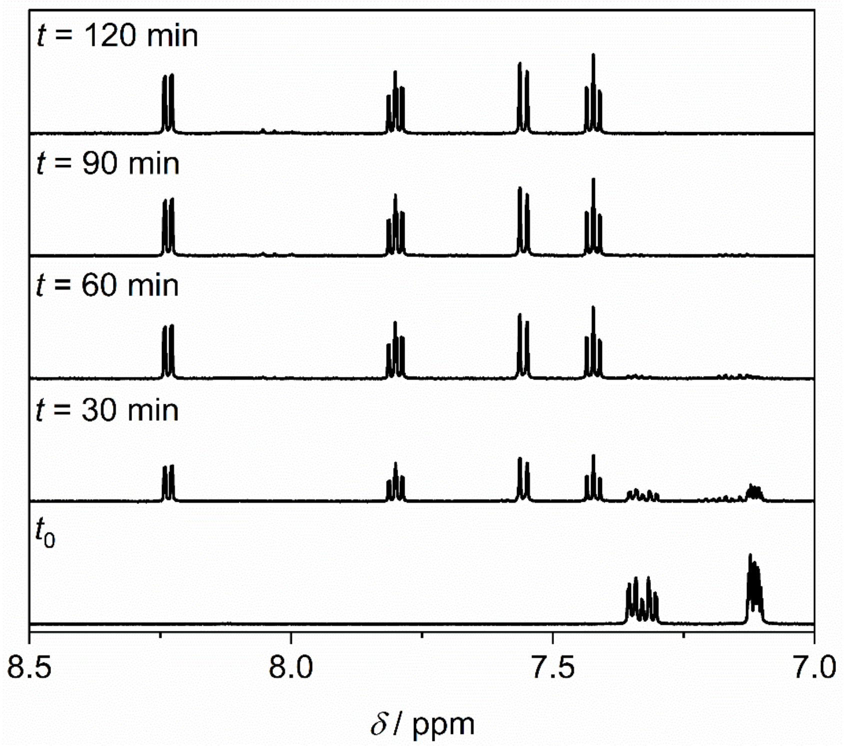

Figure S38 ^{1}H NMR spectra (600 MHz, CD_3CN, 298 K) of the reaction mixture of xanthene-9-carboxylic acid and **SCNP1-Flow** before (t_0) and at different times after starting the irradiation with a 400 nm LED (10 W, refer to **Figure S27** for the emission spectrum) under oxygen atmosphere. Experiment 3.

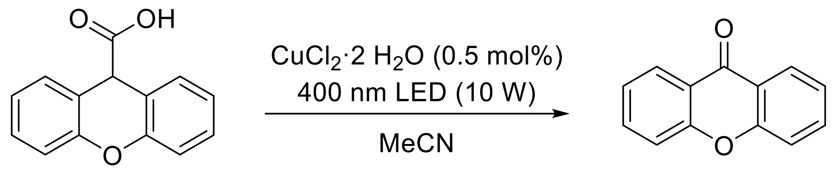

For reactions employing 0.5 mol% of $CuCl_2 \cdot 2\,H_2O$ as the catalyst, 11.3 mg xanthene-9-carboxylic acid (49.8 µmol, 1.00 eq.) were dissolved in 5 mL of a 0.01 mg·mL^{-1} $CuCl_2 \cdot 2\,H_2O$ stock solution (0.293 µmol, 0.00588 eq.) in acetonitrile in a 10 mL crimp vial. The resulting yellow solution was sparged with oxygen and an oxygen-filled balloon was fitted on top of the vial. The solution was irradiated using a 400 nm LED (10 W, refer to **Figure S27** for the emission spectrum). 0.1 mL of the reaction solution were taken out before irradiation (t_0) and after 30, 60 and 90 minutes, respectively, and 0.3 mL of CD_3CN added for NMR analysis. The reaction was performed in triplicate (refer to **Figure S39** to **S41**).

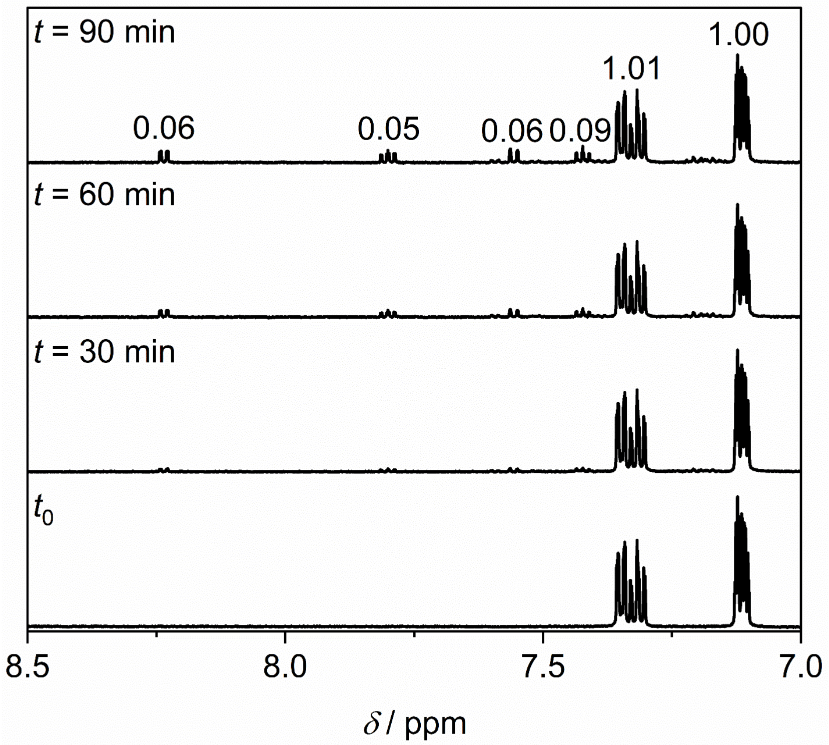

Figure S39 ^{1}H NMR spectra (600 MHz, CD_3CN, 298 K) of the reaction mixture of xanthene-9-carboxylic acid and 0.5 mol% $CuCl_2 \cdot 2\,H_2O$ before (t_0) and at different times after starting the irradiation with a 400 nm LED (10 W, refer to **Figure S27** for the emission spectrum) under oxygen atmosphere. Numbers on peaks denote integral values. Experiment 1.

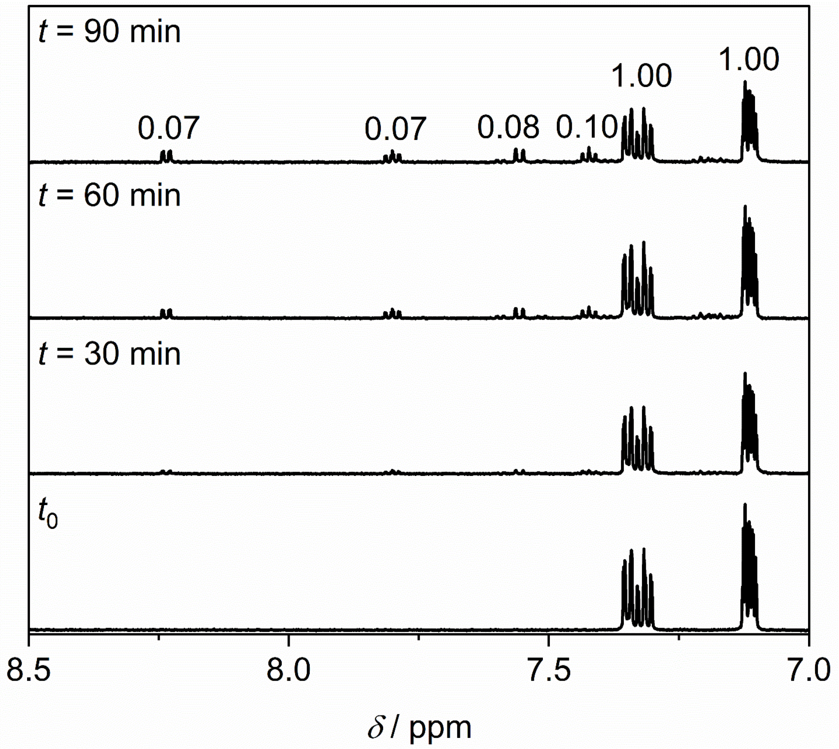

Figure S40 ^{1}H NMR spectra (600 MHz, CD_3CN, 298 K) of the reaction mixture of xanthene-9-carboxylic acid and 0.5 mol% $CuCl_2$·2 H_2O before (t_0) and at different times after starting the irradiation with a 400 nm LED (10 W, refer to **Figure S27** for the emission spectrum) under oxygen atmosphere. Numbers on peaks denote integral values. Experiment 2.

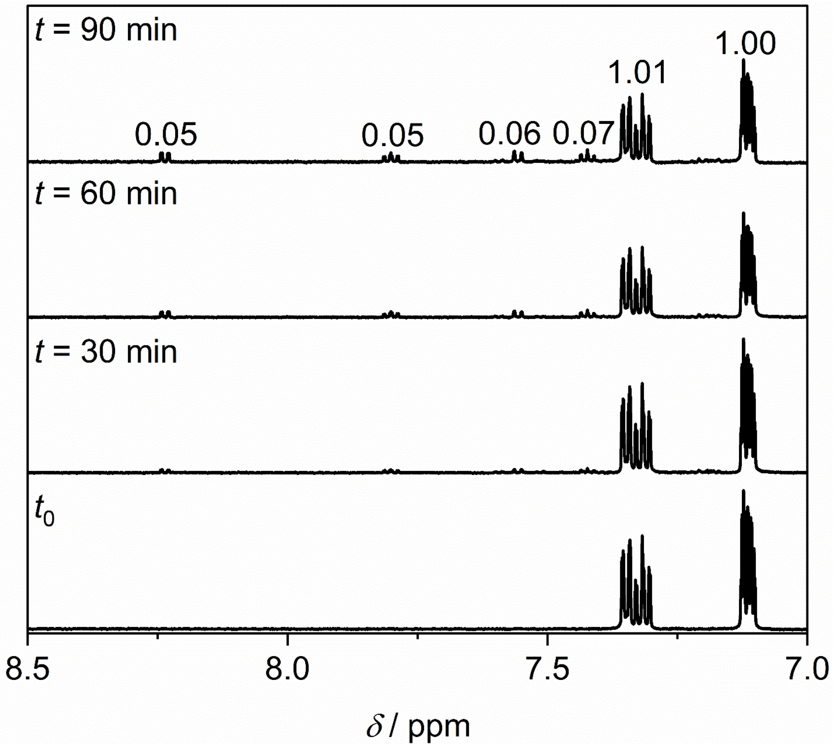

Figure S41 ^{1}H NMR spectra (600 MHz, CD_3CN, 298 K) of the reaction mixture of xanthene-9-carboxylic acid and 0.5 mol% $CuCl_2$·2 H_2O before (t_0) and at different times after starting the irradiation with a 400 nm LED (10 W, refer to **Figure S27** for the emission spectrum) under oxygen atmosphere. Numbers on peaks denote integral values. Experiment 3.

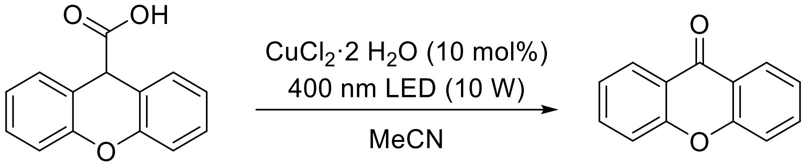

For reactions employing 10 mol% of $CuCl_2 \cdot 2\ H_2O$ as the catalyst, 11.3 mg xanthene-9-carboxylic acid (49.8 µmol, 1.00 eq.) and 0.84 mg $CuCl_2 \cdot 2\ H_2O$ (4.98 µmol, 0.10 eq.) were dissolved in 5 mL of acetonitrile in a 20 mL crimp vial. The resulting yellow solution was sparged with oxygen and an oxygen-filled balloon was fitted on top of the vial. The solution was irradiated using a 400 nm LED (10 W, refer to **Figure S27** for the emission spectrum). 0.1 mL of the reaction solution were taken out before irradiation (t_0) and after 30, 60 and 90 minutes, respectively, and 0.3 mL of CD_3CN added for NMR analysis. The reaction was performed in triplicate (refer to **Figure S42** to **S44**).

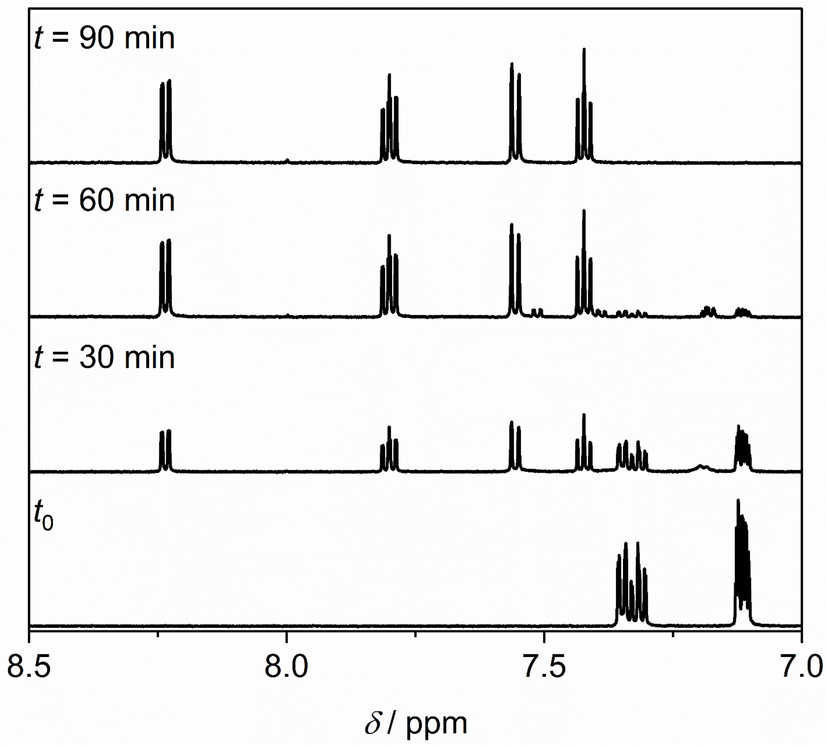

Figure S42 ^1H NMR spectra (600 MHz, CD_3CN, 298 K) of the reaction mixture of xanthene-9-carboxylic acid and 10 mol% $CuCl_2 \cdot 2\ H_2O$ before (t_0) and at different times after starting the irradiation with a 400 nm LED (10 W, refer to **Figure S27** for the emission spectrum) under oxygen atmosphere. Experiment 1.

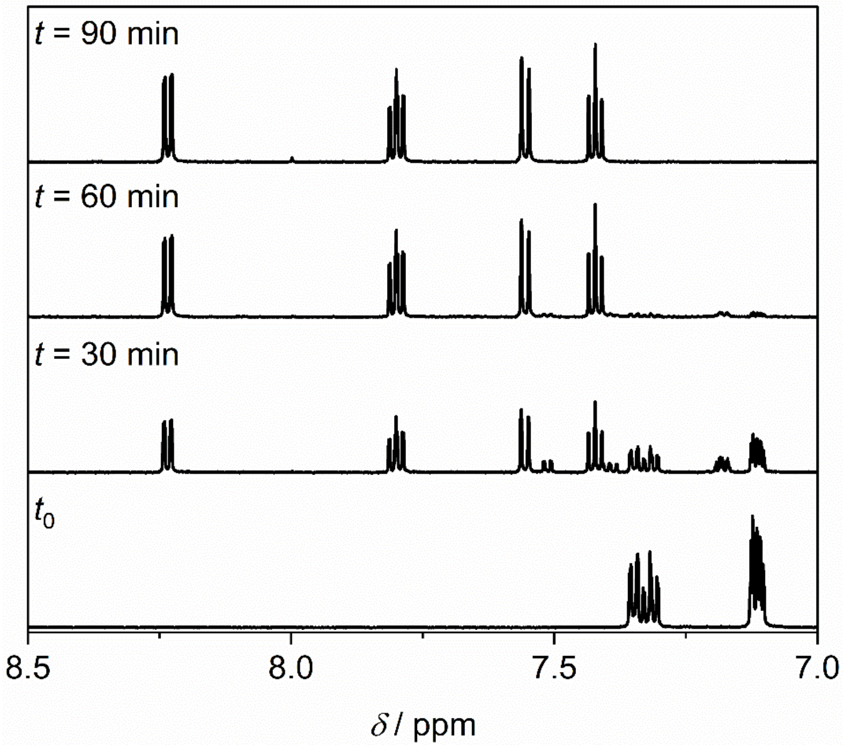

Figure S43 ^{1}H NMR spectra (600 MHz, CD_3CN, 298 K) of the reaction mixture of xanthene-9-carboxylic acid and 10 mol% $CuCl_2{\cdot}2\ H_2O$ before (t_0) and at different times after starting the irradiation with a 400 nm LED (10 W, refer to **Figure S27** for the emission spectrum) under oxygen atmosphere. Experiment 2.

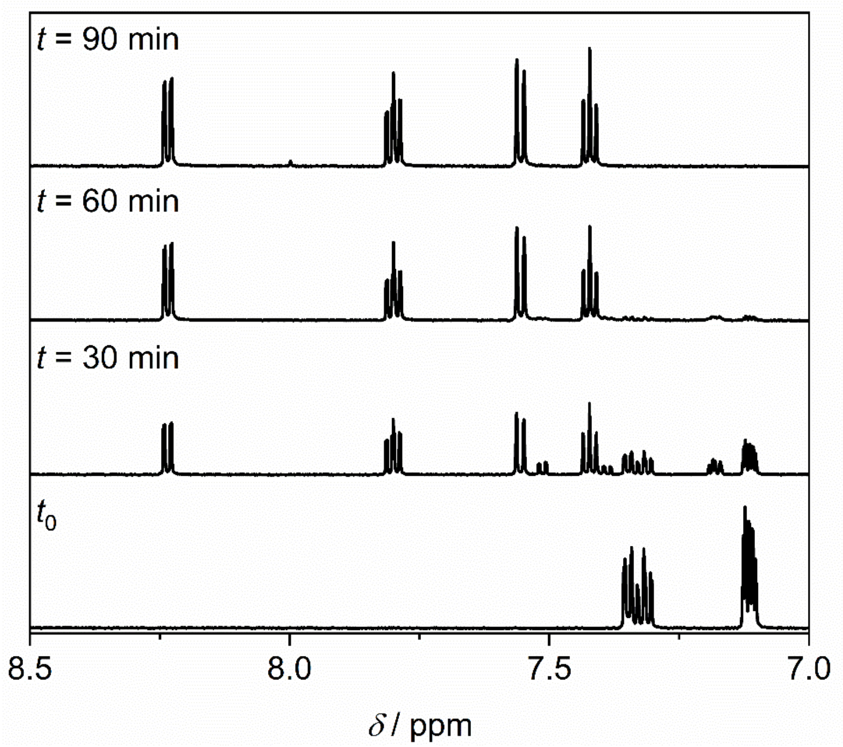

Figure S44 ^{1}H NMR spectra (600 MHz, CD_3CN, 298 K) of the reaction mixture of xanthene-9-carboxylic acid and 10 mol% $CuCl_2{\cdot}2\ H_2O$ before (t_0) and at different times after starting the irradiation with a 400 nm LED (10 W, refer to **Figure S27** for the emission spectrum) under oxygen atmosphere. Experiment 3.

The following control reactions were conducted:

(A) Irradiation only, no catalyst

11.3 mg xanthene-9-carboxylic acid (49.8 µmol, 1.00 eq.) were dissolved in 5 mL of acetonitrile in a 20 mL crimp vial. The resulting colorless solution was sparged with oxygen and an oxygen-filled balloon was fitted on top of the vial. The solution was irradiated using a 400 nm LED (10 W, refer to **Figure S27** for the emission spectrum). 0.1 mL of the reaction solution were taken out before irradiation (t_0) and 120 minutes after starting the irradiation, respectively, and 0.3 mL CD_3CN added for NMR analysis (refer to **Figure S45**).

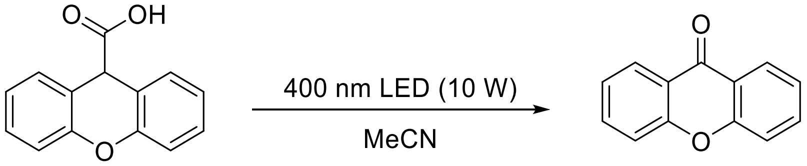

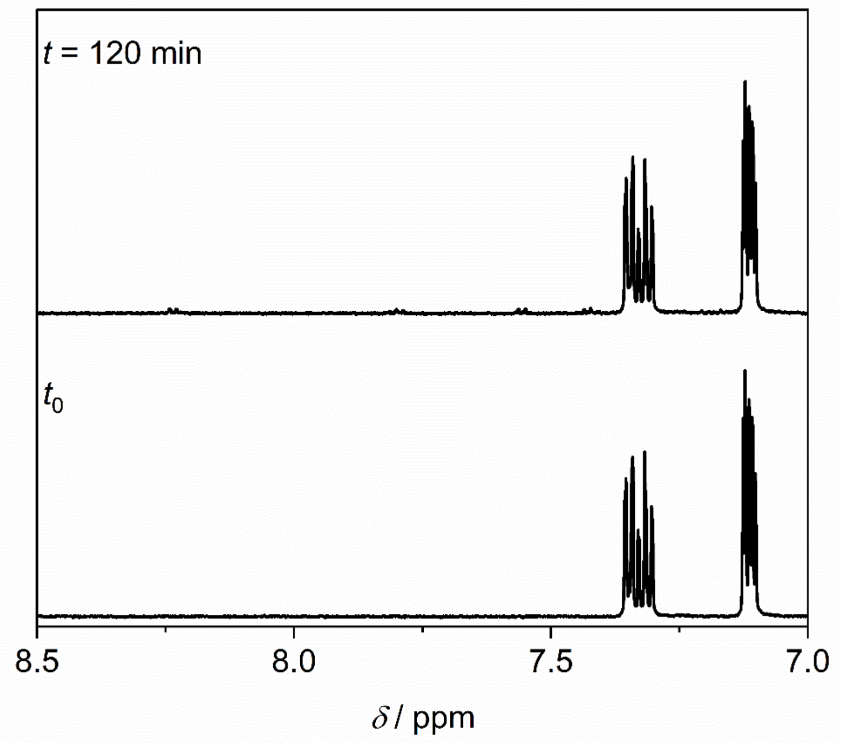

Figure S45 ^1H NMR spectra (600 MHz, CD_3CN, 298 K) of xanthene-9-carboxylic acid before (t_0) and after 120 minutes of irradiation with a 400 nm LED (10 W, refer to **Figure S27** for the emission spectrum) under oxygen atmosphere.

(B) Unfolded precursor polymer + irradiation

11.3 mg xanthene-9-carboxylic acid (49.8 µmol, 1.00 eq.) and 10.0 mg **P1'** (4.98 µmol, 0.10 eq. photoreactive unit) were dissolved in 5 mL of acetonitrile in a 20 mL crimp vial. The resulting colorless solution was sparged with oxygen and an oxygen-filled balloon was fitted on top of the vial. The solution was irradiated using a 400 nm LED (10 W, refer to **Figure S27** for the emission spectrum). 0.1 mL of the reaction solution were taken out before irradiation (t_0) and after 120 minutes, respectively, and 0.3 mL of CD_3CN added for NMR analysis (refer to **Figure S46**).

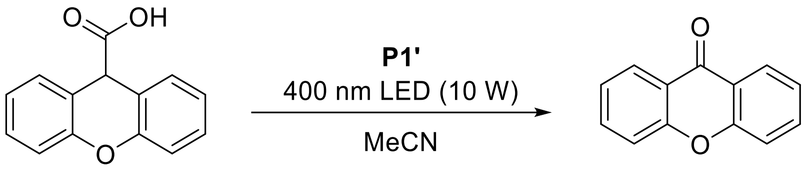

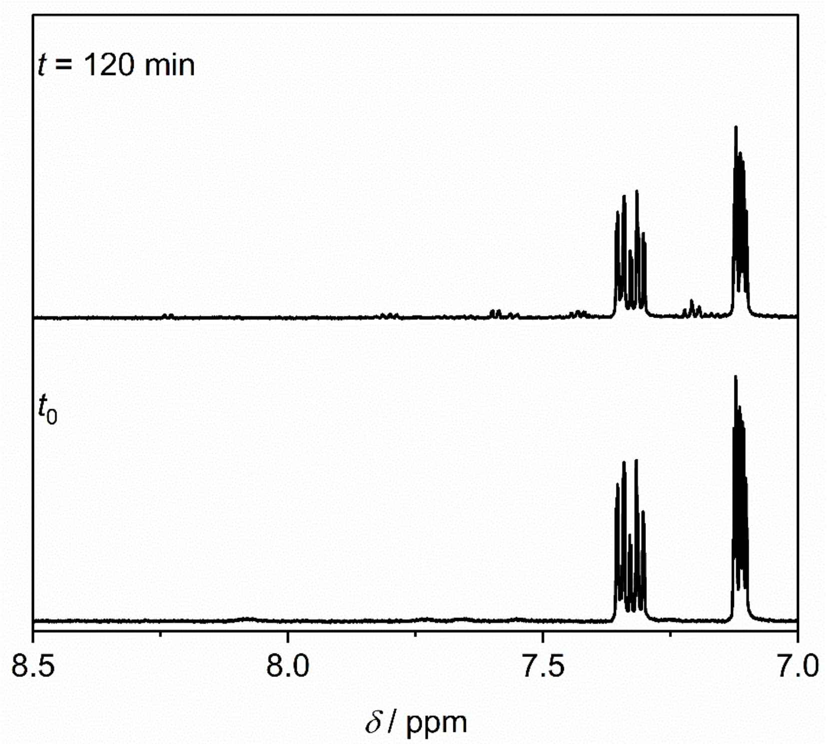

Figure S46 ^1H NMR spectra (600 MHz, CD_3CN, 298 K) of the reaction mixture of xanthene-9-carboxylic acid and **P1'** before (t_0) and after 120 minutes of irradiation with a 400 nm LED (10 W, refer to **Figure S27** for the emission spectrum) under oxygen atmosphere.

(C) Mixture of unfolded precursor polymer + $CuCl_2$·2 H_2O subjected to preparative SEC

10.0 mg of polymer **P1'** (4.98 µmol, 0.10 eq. photoreactive unit) and 0.84 mg $CuCl_2$·2 H_2O (4.98 µmol, 0.10 eq.) were dissolved in 5 mL of acetonitrile. The resulting yellow solution was directly loaded onto a preparative SEC column (Sephadex LH-20) and eluted with acetonitrile, resulting in a colorless solution of **P1'**. The solution was transferred into a 20 mL crimp vial and 11.3 mg xanthene-9-carboxylic acid (49.8 µmol, 1.00 eq.) were added. After sparging with oxygen, an oxygen-filled balloon was fitted on top of the vial and the solution irradiated using a 400 nm LED (10 W, refer to **Figure S27** for the emission spectrum). 0.1 mL of the reaction solution were taken out before irradiation (t_0) and after 120 minutes, respectively, and 0.3 mL of CD_3CN added for NMR analysis (refer to **Figure S47**).

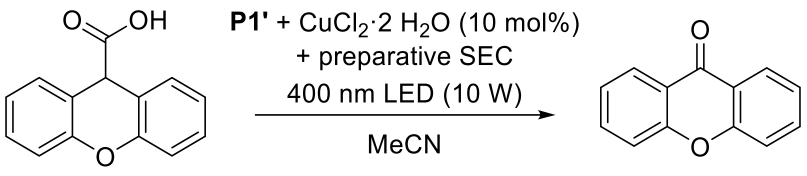

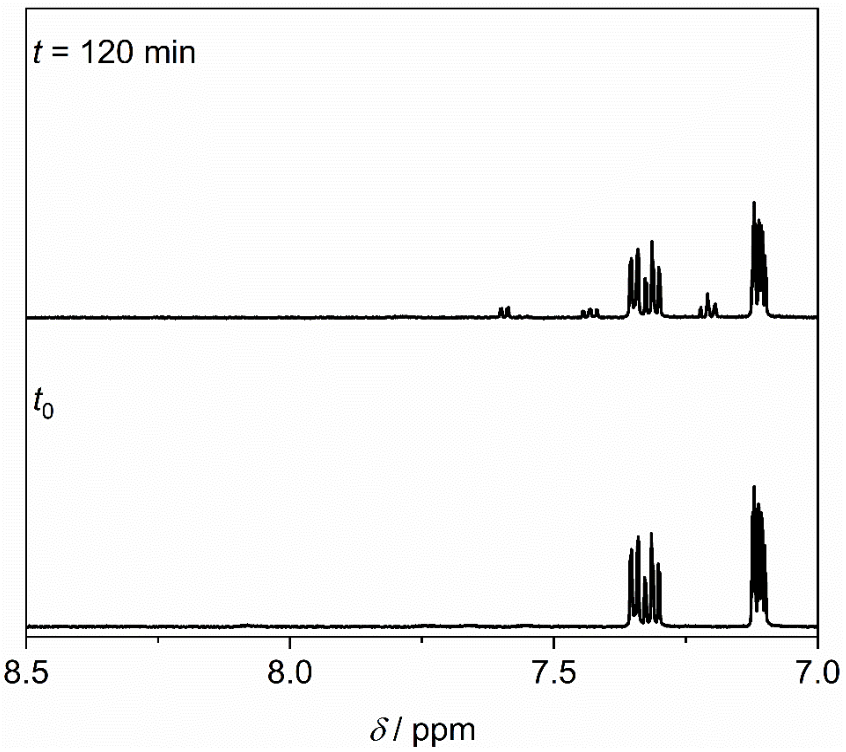

Figure S47 ^1H NMR spectra (600 MHz, CD_3CN, 298 K) of the reaction mixture of xanthene-9-carboxylic acid and **P1'** before (t_0) and after 120 minutes of irradiation with a 400 nm LED (10 W, refer to **Figure S27** for the emission spectrum) under oxygen atmosphere. For that, **P1'** and $CuCl_2$·2 H_2O were first mixed and subsequently subjected to preparative size exclusion chromatography (Sephadex LH-20) in acetonitrile, demonstrating that catalytically active $CuCl_2$·2 H_2O is efficiently removed by this purification technique (compare **Figure S42** to **S34** and **Figure S46**).

(D) $CuCl_2$·2 H_2O without irradiation

11.3 mg xanthene-9-carboxylic acid (49.8 µmol, 1.00 eq.) and 0.84 mg $CuCl_2$·2 H_2O (4.98 µmol, 0.10 eq.) were dissolved in 5 mL of acetonitrile in a 20 mL crimp vial. The resulting yellow solution was sparged with oxygen and an oxygen-filled balloon was fitted on top of the vial. The solution was stirred at room temperature without irradiation. 0.1 mL of the reaction solution were taken out immediately (t_0) and after 120 minutes, respectively, and 0.3 mL of CD_3CN added for NMR analysis (refer to **Figure S48**).

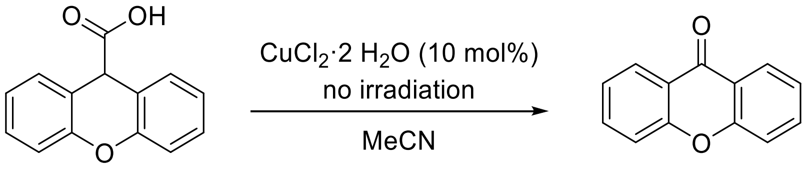

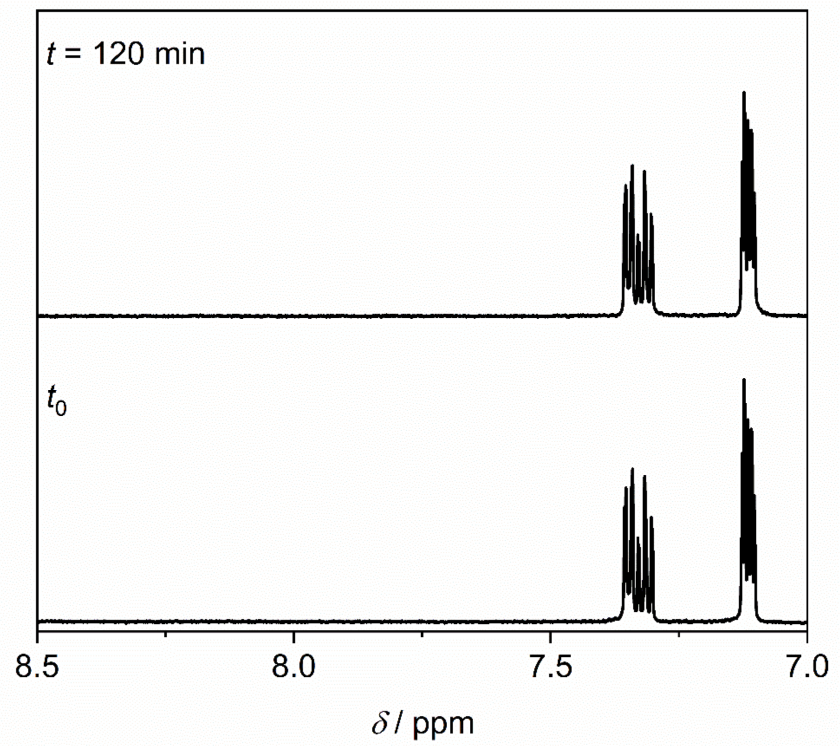

Figure S48 ^{1}H NMR spectra (600 MHz, CD_3CN, 298 K) of the reaction mixture of xanthene-9-carboxylic acid and 10 mol% $CuCl_2$·2 H_2O before (t_0) and after 120 minutes of stirring at room temperature without irradiation under oxygen atmosphere.

(E) SCNP1-Flow without irradiation

10.0 mg of polymer **P1’** (4.98 µmol, 0.10 eq. photoreactive unit) and 0.84 mg $CuCl_2 \cdot 2\ H_2O$ (4.98 µmol, 0.10 eq.) were dissolved in 5 mL of acetonitrile. The resulting yellow solution was sparged with argon and subsequently irradiated using a 410 nm LED (12 W, refer to **Figure S27** for the emission spectrum) under photoflow conditions (flowrate 0.2 mL·min^{-1}, refer to Chapter 2.7 for details regarding the photoflow reactor). The obtained yellow solution was directly loaded onto a preparative SEC column (Sephadex LH-20) and eluted with acetonitrile, resulting in a pale-yellow solution of **SCNP1-Flow**. The solution was transferred into a 20 mL crimp vial and 11.3 mg xanthene-9-carboxylic acid (49.8 µmol, 1.00 eq.) were added. After sparging with oxygen, an oxygen-filled balloon was fitted on top of the vial and the solution stirred at room temperature without irradiation. 0.1 mL of the reaction solution were taken out immediately (t_0) and after 120 minutes, respectively, and 0.3 mL of CD_3CN added for NMR analysis (refer to **Figure S49**).

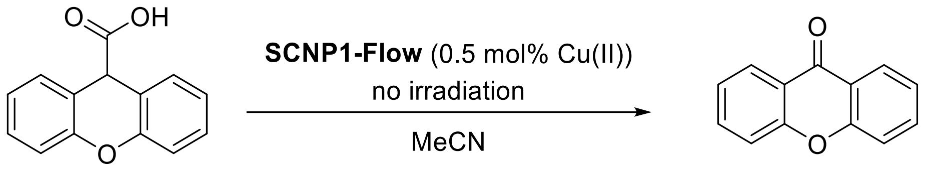

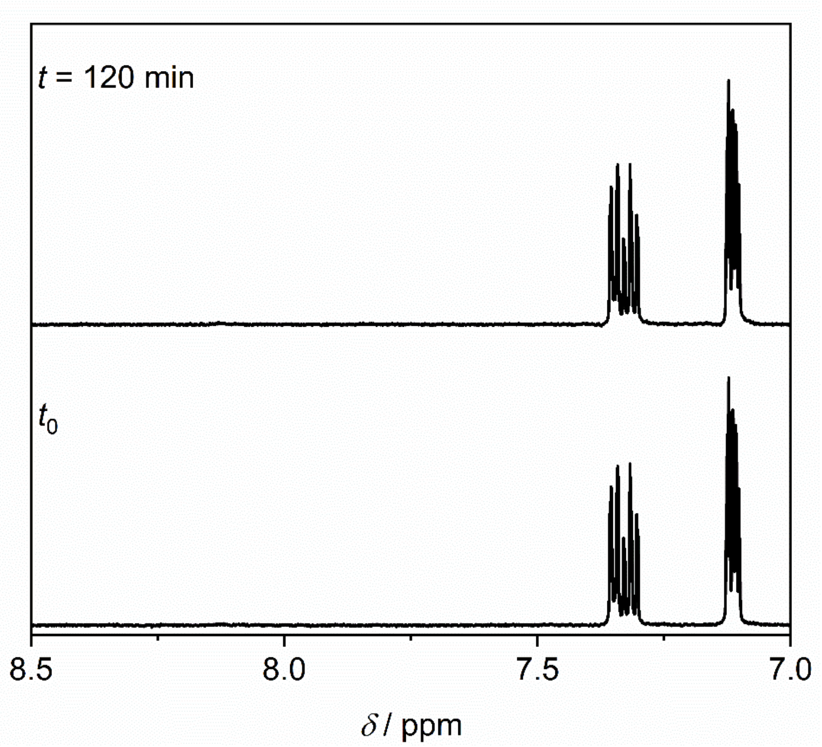

Figure S49 ^1H NMR spectra (600 MHz, CD_3CN, 298 K) of the reaction mixture of xanthene-9-carboxylic acid and **SCNP1-Flow** before (t_0) and after 120 minutes of stirring at room temperature without irradiation under oxygen atmosphere.

(F) $CuCl_2·2\ H_2O$ + unfunctionalized Poly(PEGMEMA)

11.3 mg xanthene-9-carboxylic acid (49.8 µmol, 1.00 eq.) and 10.0 mg Poly(PEGMEMA) were dissolved in 5 mL of a 0.01 $mg·mL^{-1}$ $CuCl_2·2\ H_2O$ stock solution (0.293 µmol, 0.00588 eq.) in acetonitrile in a 10 mL crimp vial. The resulting slightly yellow solution was sparged with oxygen and an oxygen-filled balloon was fitted on top of the vial. The solution was irradiated using a 400 nm LED (10 W, refer to **Figure S27** for the emission spectrum). 0.1 mL of the reaction solution were taken out before irradiation (t_0) and after 30, 60 and 90 minutes, respectively, and 0.3 mL of CD_3CN added for NMR analysis (refer to **Figure S50**).

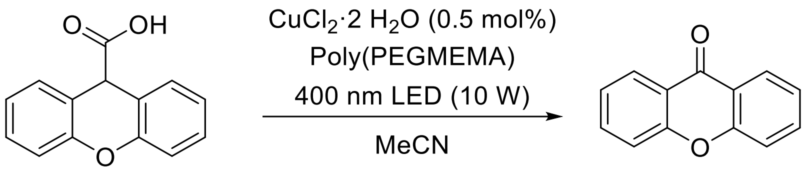

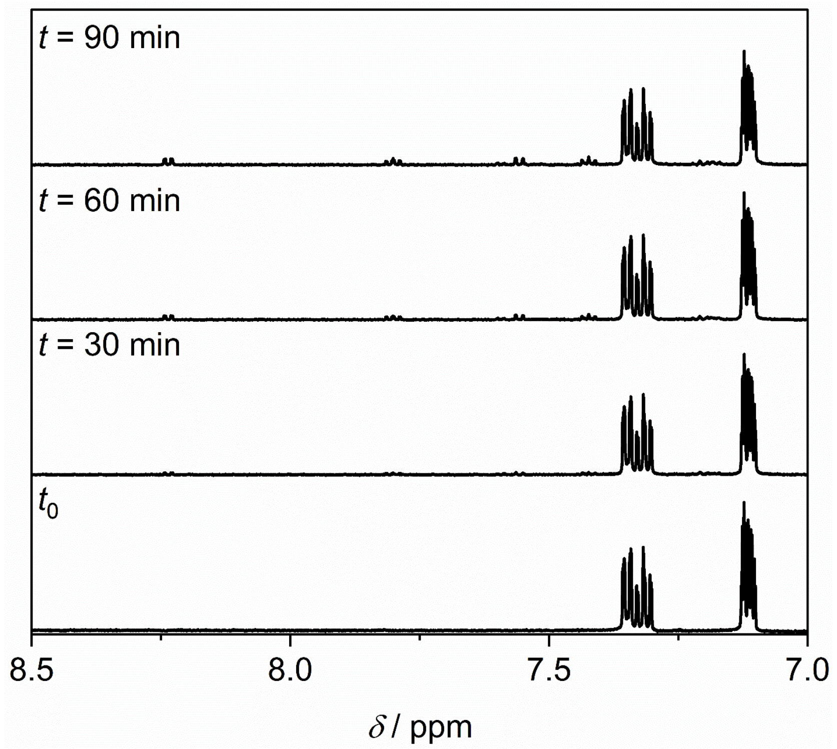

Figure S50 ^1H NMR spectra (600 MHz, CD_3CN, 298 K) of the reaction mixture of xanthene-9-carboxylic acid, 0.5 mol% $CuCl_2·2\ H_2O$ and Poly(PEGMEMA) before (t_0) and at different times after starting the irradiation with a 400 nm LED (10 W, refer to **Figure S27** for the emission spectrum) under oxygen atmosphere.

(G) $CuCl_2$·2 H_2O + *n*-Butylamine

A stock solution containing 0.01 mg·mL^{-1} of $CuCl_2$·2 H_2O and 17.2 µg·mL^{-1} *n*-butylamine in acetonitrile was prepared. 11.3 mg xanthene-9-carboxylic acid (49.8 µmol, 1.00 eq.) were dissolved in 5 mL of this acetonitrile solution (0.293 µmol, 0.00588 eq. $CuCl_2$·2 H_2O; 1.18 µmol, 0.0235 eq. *n*-butylamine) in a 10 mL crimp vial. The resulting slightly blue solution was sparged with oxygen and an oxygen-filled balloon was fitted on top of the vial. The solution was irradiated using a 400 nm LED (10 W, refer to **Figure S27** for the emission spectrum). 0.1 mL of the reaction solution were taken out before irradiation (t_0) and after 30, 60, 90 and 120 minutes, respectively, and 0.3 mL of CD_3CN added for NMR analysis (refer to **Figure S51**).

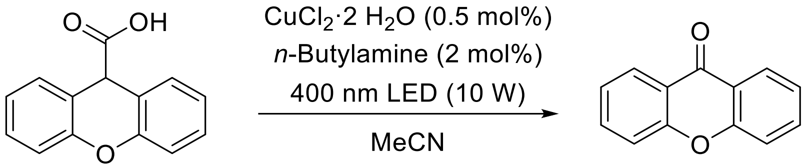

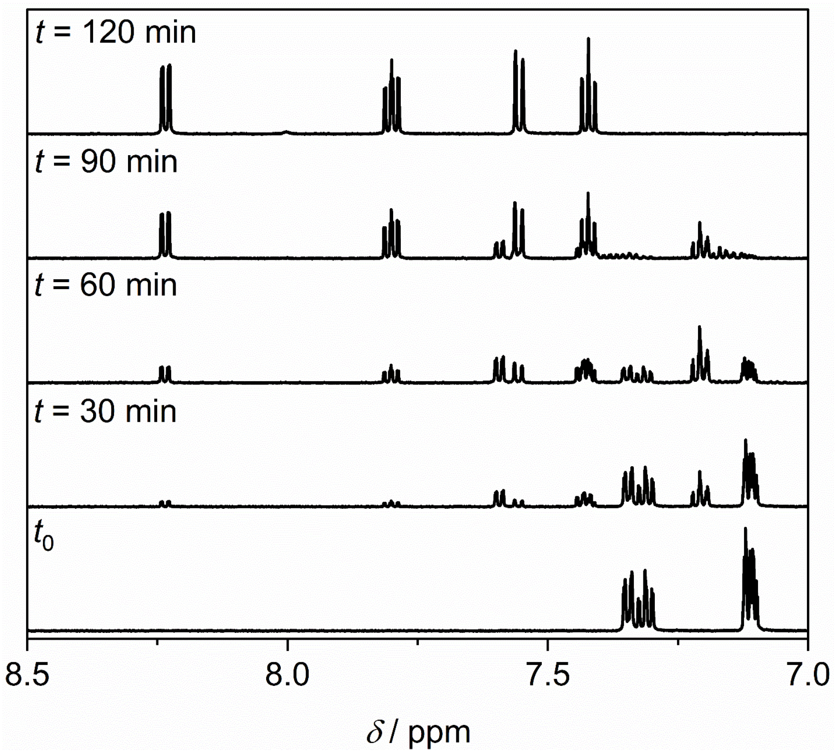

Figure S51 ^1H NMR spectra (600 MHz, CD_3CN, 298 K) of the reaction mixture of xanthene-9-carboxylic acid, 0.5 mol% $CuCl_2$·2 H_2O and 2 mol% *n*-butylamine before (t_0) and at different times after starting the irradiation with a 400 nm LED (10 W, refer to **Figure S27** for the emission spectrum) under oxygen atmosphere.

(H) SCNP1-Batch

10.0 mg of **P1'** (4.98 µmol, 0.10 eq. photoreactive unit) and 0.84 mg of $CuCl_2 \cdot 2\ H_2O$ (4.98 µmol, 0.10 eq.) were dissolved in 5 mL of acetonitrile. The resulting yellow solution was sparged with argon and subsequently irradiated using a 400 nm LED (10 W, refer to **Figure S27** for the emission spectrum) for 50 minutes. The obtained yellow solution was directly loaded onto a preparative SEC column (Sephadex LH-20) and eluted with acetonitrile, resulting in a pale-yellow solution of **SCNP1-Batch**.

The solution was transferred into a 20 mL crimp vial and 11.3 mg xanthene-9-carboxylic acid (49.8 µmol, 1.00 eq.) were added. After sparging with oxygen, an oxygen-filled balloon was fitted on top of the vial and the solution was irradiated using a 400 nm LED (10 W, refer to **Figure S27** for the emission spectrum). 0.1 mL of the reaction solution were taken out before irradiation (t_0) and after 30, 60, 90 and 120 minutes, respectively, and 0.3 mL of CD_3CN added for NMR analysis (refer to **Figure S52**).

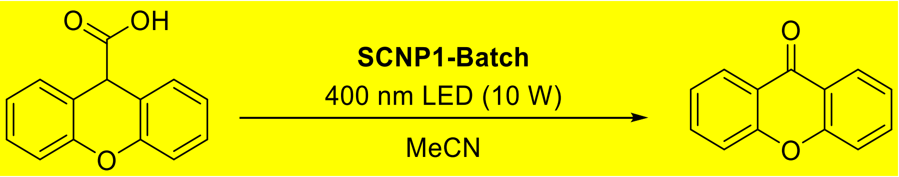

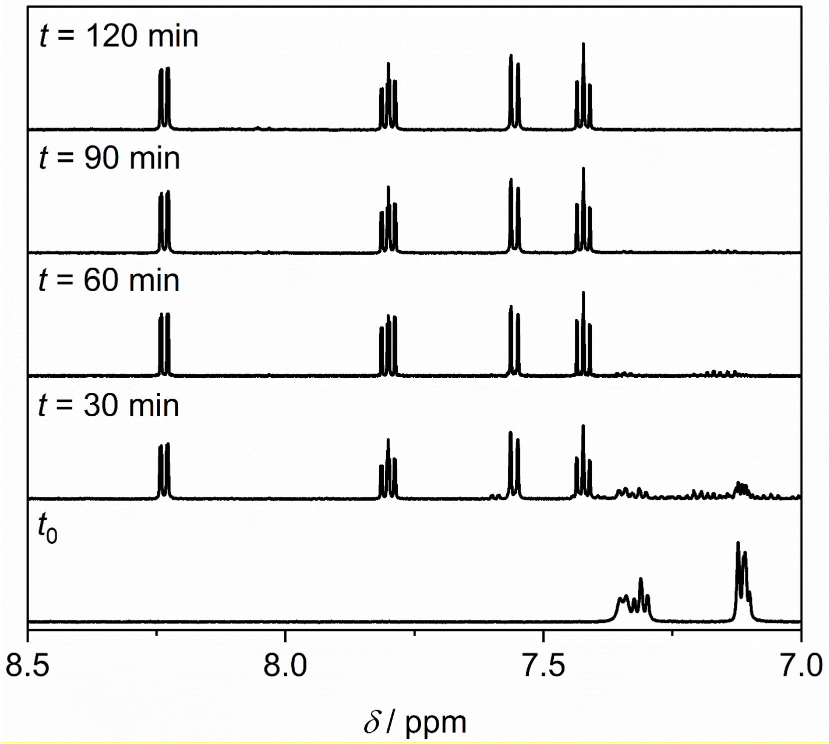

Figure S52 ^1H NMR spectra (600 MHz, CD_3CN, 298 K) of the reaction mixture of xanthene-9-carboxylic acid and **SCNP1-Batch** before (t_0) and at different times after starting the irradiation with a 400 nm LED (10 W, refer to **Figure S27** for the emission spectrum) under oxygen atmosphere.

6.2 Oxidative Double Bond Cleavage of Oleic acid

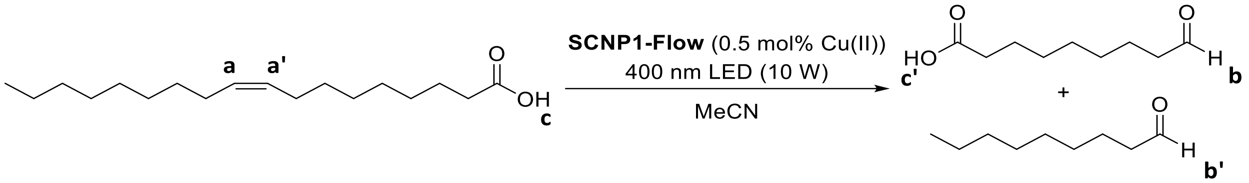

Scheme S2 Reaction scheme for the Cu(II) photocatalyzed oxidative cleavage of the double bond of oleic acid. Labels refer to **Figure S53**.

For reactions employing **SCNP1-Flow** as the catalyst, 10.0 mg of polymer **P1'** (4.98 µmol, 0.10 eq. photoreactive unit) and 0.84 mg $CuCl_2 \cdot 2\ H_2O$ (4.98 µmol, 0.10 eq.) were dissolved in 5 mL of acetonitrile. The resulting yellow solution was sparged with argon and subsequently irradiated using a 410 nm LED (12 W, refer to **Figure S27** for the emission spectrum) under photoflow conditions (flowrate 0.2 $mL \cdot min^{-1}$, refer to Chapter 2.7 for details regarding the photoflow reactor). The obtained yellow solution was directly loaded onto a preparative SEC column (Sephadex LH-20) and eluted with acetonitrile, resulting in 5 mL of a pale-yellow solution of **SCNP1-Flow** (Cu(II) concentration about 0.05 $mmol \cdot L^{-1}$, refer to Chapter 5.9). The solution was transferred into a 20 mL crimp vial and 15.6 µL oleic acid (14.1 mg, 49.8 µmol, 1.00 eq.) were added. After sparging with oxygen, an oxygen-filled balloon was fitted on top of the vial and the solution irradiated using a 400 nm LED (10 W, refer to **Figure S27** for the emission spectrum). 0.1 mL of the reaction solution were taken out before irradiation (t_0) and after 1, 3, 5 and 24 hours, respectively, and 0.3 mL of CD_3CN added for NMR analysis. The reaction was performed in triplicate (refer to **Figure S53** to **S55**).

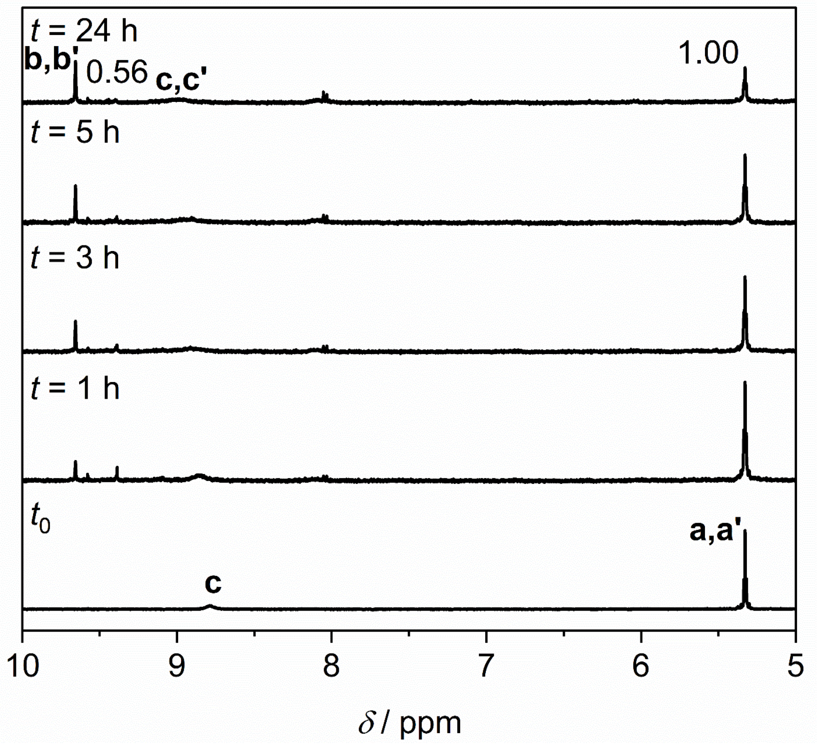

Figure S53 ^1H NMR spectra (600 MHz, CD_3CN, 298 K) of the reaction mixture of oleic acid and **SCNP1-Flow** before (t_0) and at different times after starting the irradiation with a 400 nm LED (10 W, refer to **Figure S27** for the emission spectrum) under oxygen atmosphere. Numbers on peaks denote integral values. Labels refer to **Scheme S2**. Experiment 1.

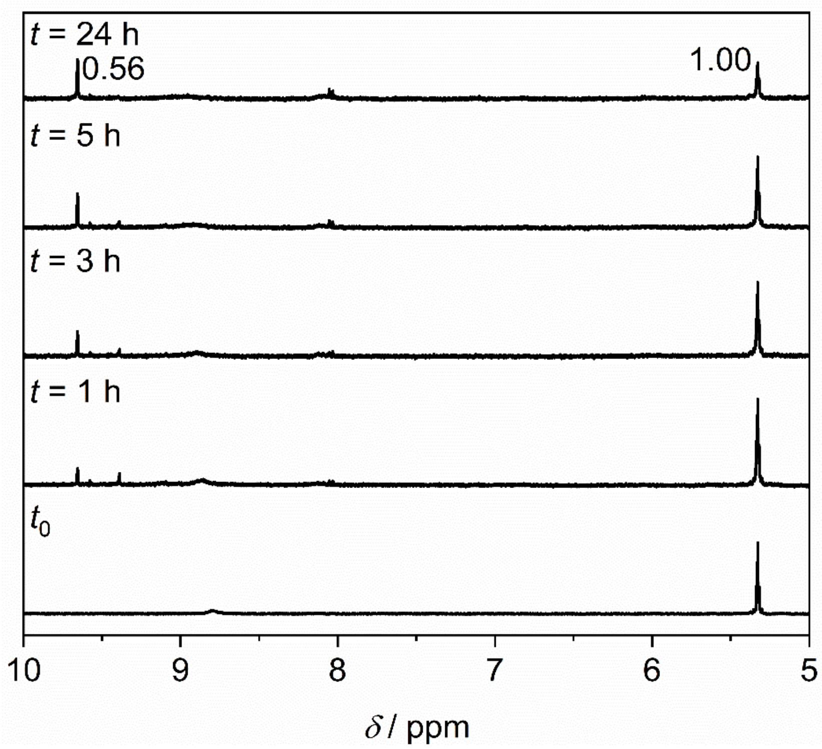

Figure S54 ^{1}H NMR spectra (600 MHz, CD_3CN, 298 K) of the reaction mixture of oleic acid and **SCNP1-Flow** before (t_0) and at different times after starting the irradiation with a 400 nm LED (10 W, refer to **Figure S27** for the emission spectrum) under oxygen atmosphere. Numbers on peaks denote integral values. Experiment 2.

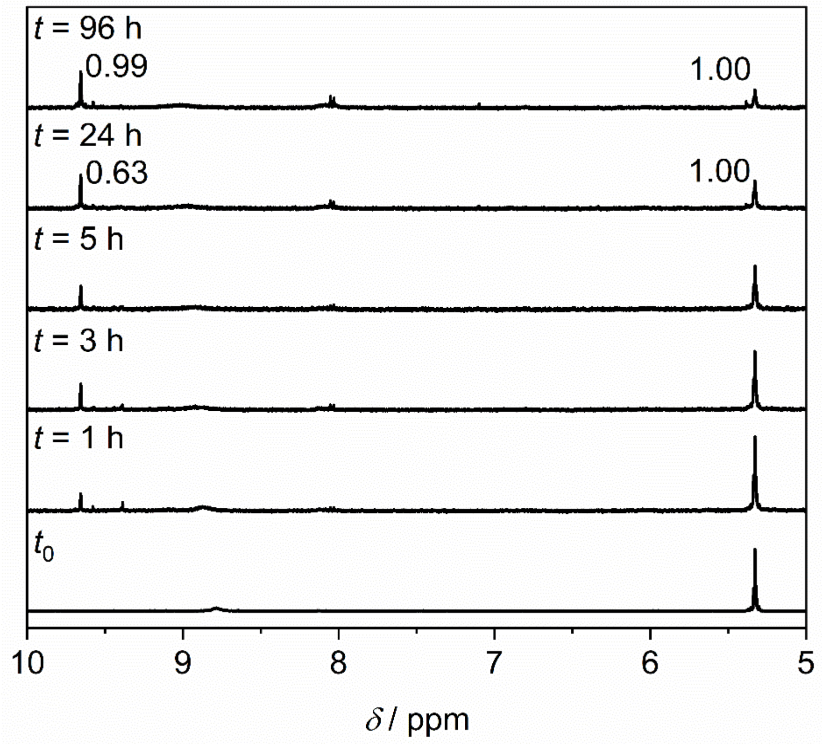

Figure S55 ^{1}H NMR spectra (600 MHz, CD_3CN, 298 K) of the reaction mixture of oleic acid and **SCNP1-Flow** before (t_0) and at different times after starting the irradiation with a 400 nm LED (10 W, refer to **Figure S27** for the emission spectrum) under oxygen atmosphere. Numbers on peaks denote integral values. Experiment 3.

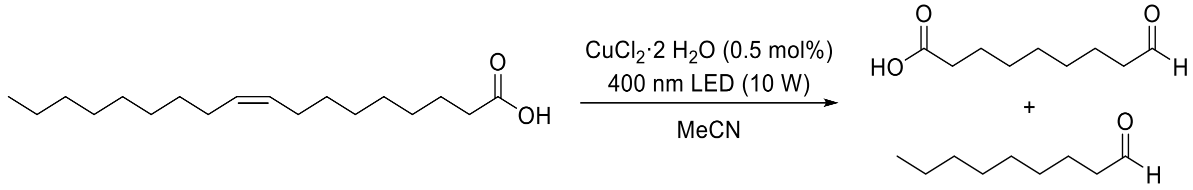

For reactions employing 0.5 mol% $CuCl_2$·2 H_2O as the catalyst, 15.6 µL oleic acid (14.1 mg, 49.8 µmol, 1.00 eq.) were dissolved in 5 mL of a 0.01 mg·mL^{-1} $CuCl_2$·2 H_2O stock solution (0.293 µmol, 0.00588 eq.) in acetonitrile in a 10 mL crimp vial. The resulting yellow solution was sparged with oxygen and an oxygen-filled balloon was fitted on top of the vial. The solution was irradiated using a 400 nm LED (10 W, refer to **Figure S27** for the emission spectrum). 0.1 mL of the reaction solution were taken out before irradiation (t_0) and after 1, 3, 5 and 24 hours, respectively, and 0.3 mL of CD_3CN added for NMR analysis. The reaction was performed in triplicate (refer to **Figure S56** to **S58**).

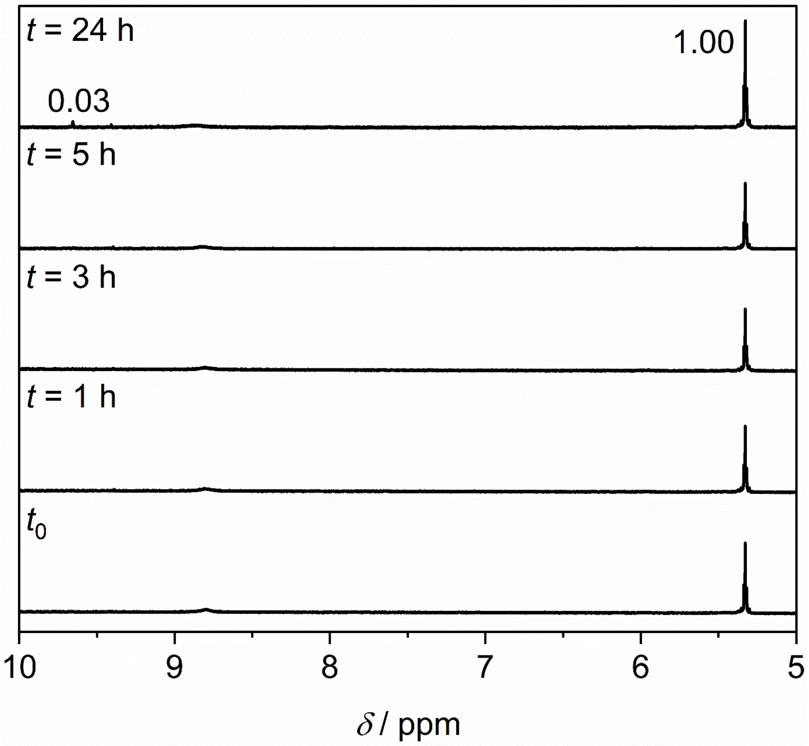

Figure S56 ^1H NMR spectra (600 MHz, CD_3CN, 298 K) of the reaction mixture of oleic acid and 0.5 mol% $CuCl_2$·2 H_2O before (t_0) and at different times after starting the irradiation with a 400 nm LED (10 W, refer to **Figure S27** for the emission spectrum) under oxygen atmosphere. Numbers on peaks denote integral values. Experiment 1.

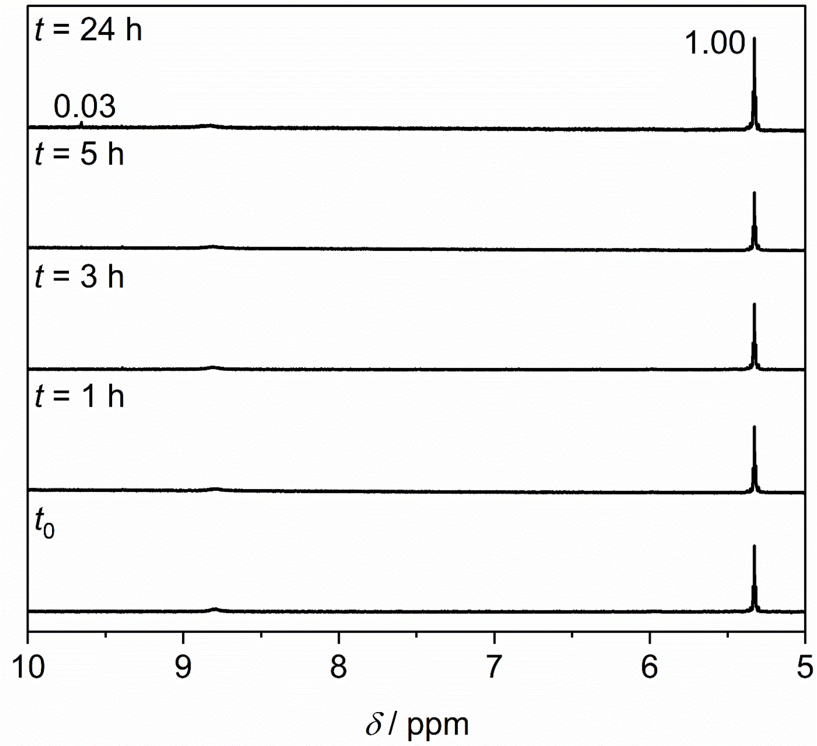

Figure S57 ^{1}H NMR spectra (600 MHz, CD_3CN, 298 K) of the reaction mixture of oleic acid and 0.5 mol% $CuCl_2{\cdot}2\ H_2O$ before (t_0) and at different times after starting the irradiation with a 400 nm LED (10 W, refer to **Figure S27** for the emission spectrum) under oxygen atmosphere. Numbers on peaks denote integral values. Experiment 2.

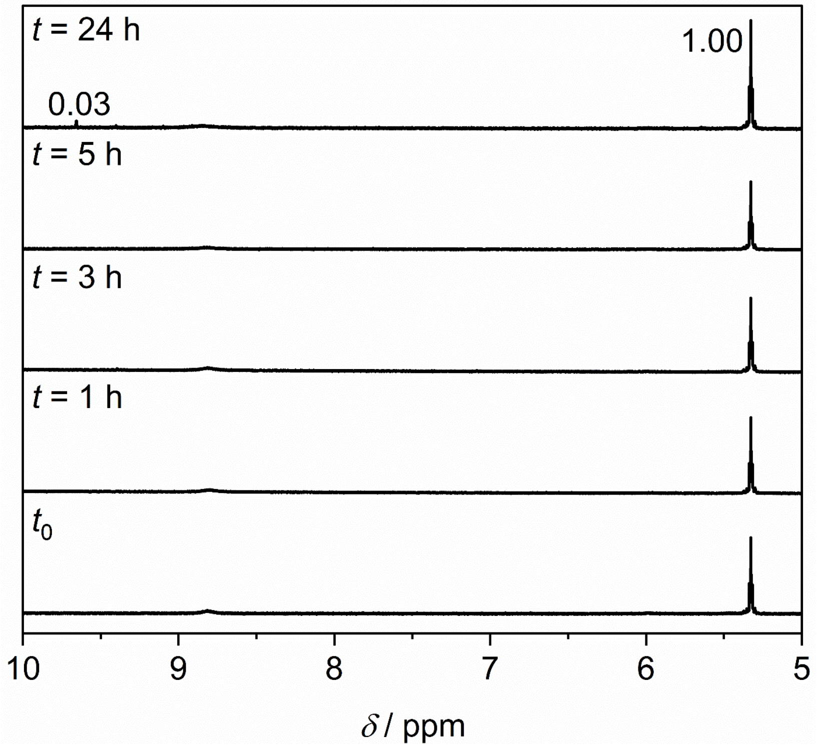

Figure S58 ^{1}H NMR spectra (600 MHz, CD_3CN, 298 K) of the reaction mixture of oleic acid and 0.5 mol% $CuCl_2{\cdot}2\ H_2O$ before (t_0) and at different times after starting the irradiation with a 400 nm LED (10 W, refer to **Figure S27** for the emission spectrum) under oxygen atmosphere. Numbers on peaks denote integral values. Experiment 3.

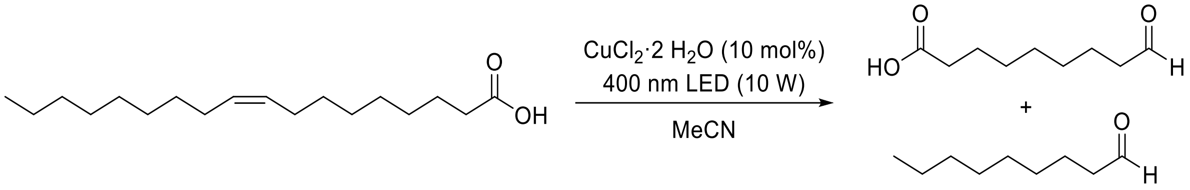

For reactions employing $CuCl_2 \cdot 2\ H_2O$ as the catalyst, 15.6 µL oleic acid (14.1 mg, 49.8 µmol, 1.00 eq.) and 0.84 mg $CuCl_2 \cdot 2\ H_2O$ (4.98 µmol, 0.10 eq.) were dissolved in 5 mL of acetonitrile in a 20 mL crimp vial. The resulting yellow solution was sparged with oxygen and an oxygen-filled balloon was fitted on top of the vial. The solution was irradiated using a 400 nm LED (10 W, refer to **Figure S27** for the emission spectrum). 0.1 mL of the reaction solution were taken out before irradiation (t_0) and after 1, 3, 5 and 24 hours, respectively, and 0.3 mL of CD_3CN added for NMR analysis. The reaction was performed in triplicate (refer to **Figure S59** to **S61**).

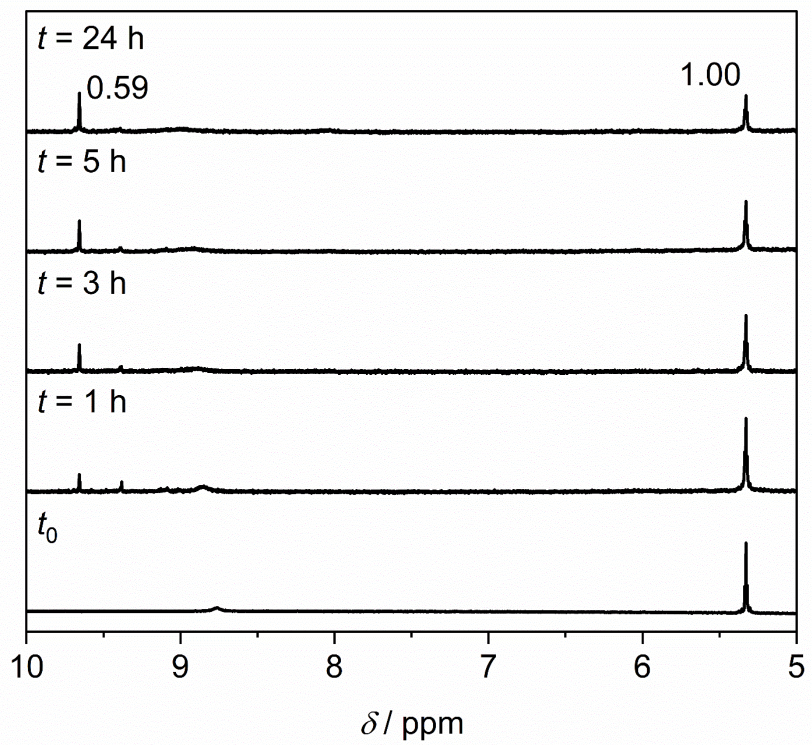

Figure S59 ^1H NMR spectra (600 MHz, CD_3CN, 298 K) of the reaction mixture of oleic acid and 10 mol% $CuCl_2 \cdot 2\ H_2O$ before (t_0) and at different times after starting the irradiation with a 400 nm LED (10 W, refer to **Figure S27** for the emission spectrum) under oxygen atmosphere. Numbers on peaks denote integral values. Experiment 1.

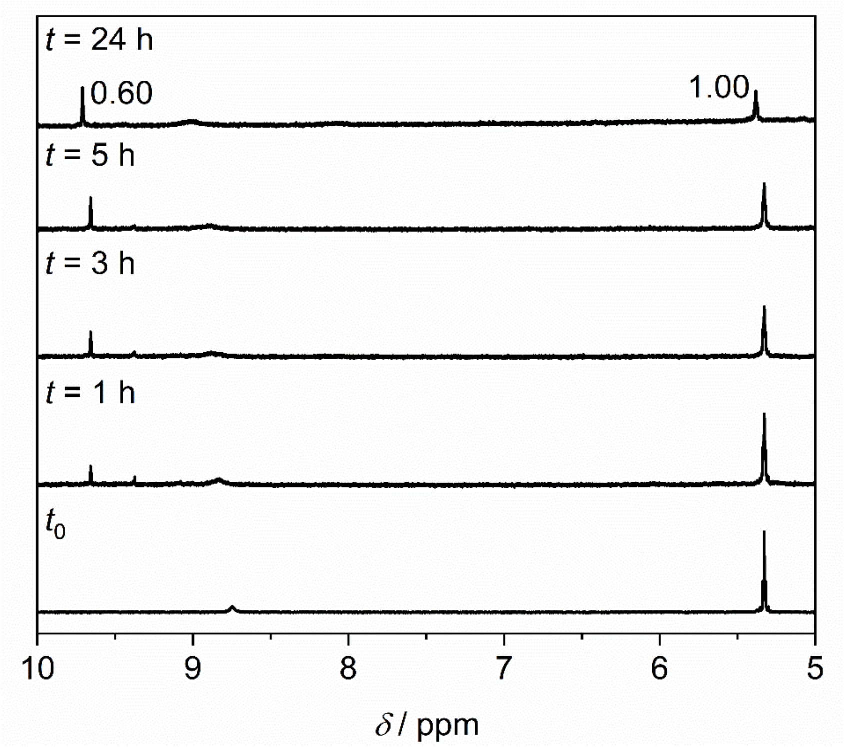

Figure S60 ^{1}H NMR spectra (600 MHz, CD_3CN, 298 K) of the reaction mixture of oleic acid and 10 mol% $CuCl_2·2\ H_2O$ before (t_0) and at different times after starting the irradiation with a 400 nm LED (10 W, refer to **Figure S27** for the emission spectrum) under oxygen atmosphere. Numbers on peaks denote integral values. Experiment 2.

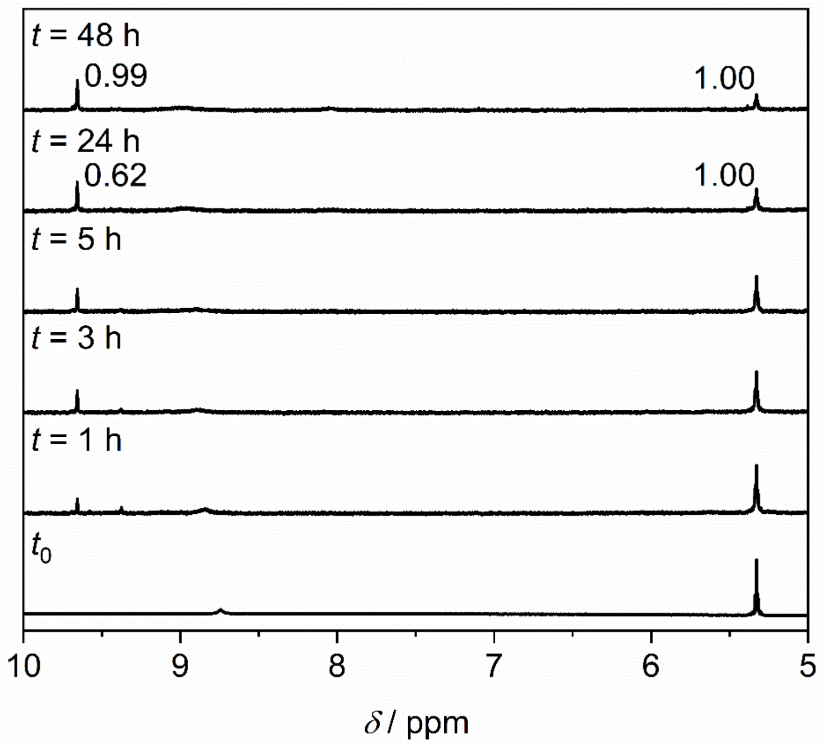

Figure S61 ^{1}H NMR spectra (600 MHz, CD_3CN, 298 K) of the reaction mixture of oleic acid and 10 mol% $CuCl_2·2\ H_2O$ before (t_0) and at different times after starting the irradiation with a 400 nm LED (10 W, refer to **Figure S27** for the emission spectrum) under oxygen atmosphere. Numbers on peaks denote integral values. Experiment 3.

To verify that the evolving resonance observed at δ = 9.66 ppm in the ^{1}H NMR spectra of the reaction mixture (refer to **Figure S59** to **S61**) actually corresponds to the cleavage reaction depicted in **Scheme S2**, the reaction progress was also monitored via LC-MS, evidencing the formation of 9-oxanonaic acid (refer to **Figure S62**).

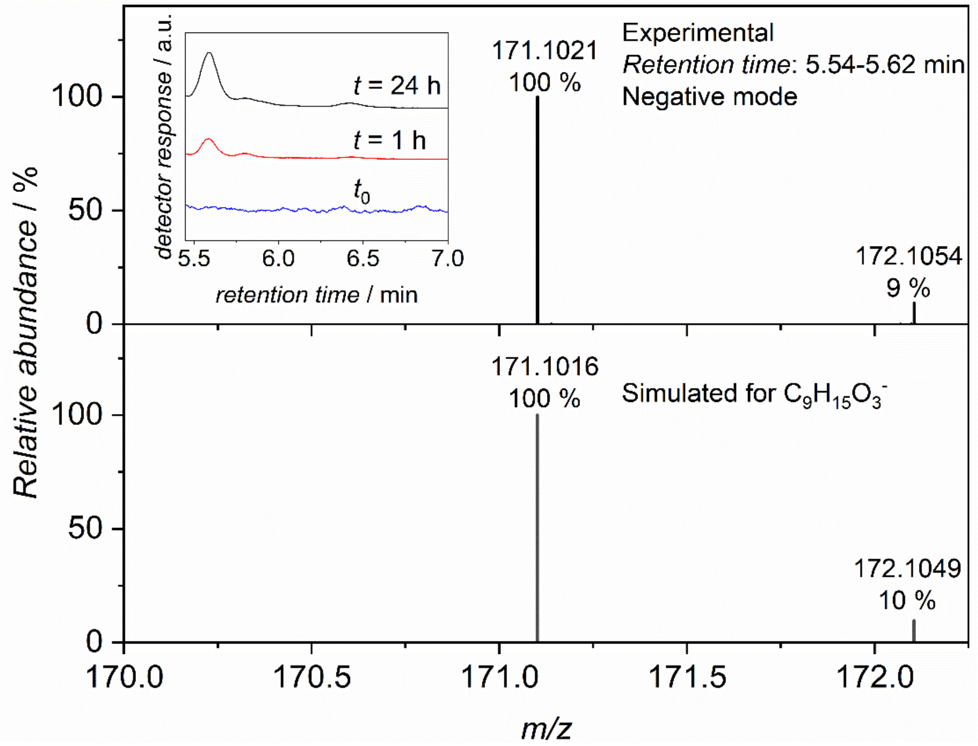

Figure S62 Expansion of the XIC for the *m/z* range 171.101-171.103 (insert) for the reaction mixture of oleic acid and 10 mol% $CuCl_2 \cdot 2\ H_2O$ before irradiation (t_0, blue) and after 1 hour (red) and 24 hours (black) of irradiation, respectively, with a 400 nm LED (10 W, refer to **Figure S27** for the emission spectrum). Top: Expansion of the accumulated mass spectrum (negative mode) obtained from the XIC peak at 5.54-5.62 minutes retention time in the chromatogram of the reaction mixture of oleic acid and $CuCl_2 \cdot 2\ H_2O$. Bottom: Simulated mass spectrum for $C_9H_{15}O_3^-$, corresponding to deprotonated 9-oxononaic acid, which is formed during the oxidative cleavage of the oleic acid double bond. A signal for nonanal could not be detected due to its low ionization tendency.

The following control reactions were conducted:

(A) Irradiation only, no catalyst

15.6 µL oleic acid (14.1 mg, 49.8 µmol, 1.00 eq.) were dissolved in 5 mL of acetonitrile in a 20 mL crimp vial. The resulting colorless solution was sparged with oxygen and an oxygen-filled balloon was fitted on top of the vial. The solution was irradiated using a 400 nm LED (10 W, refer to **Figure S27** for the emission spectrum). 0.1 mL of the reaction solution were taken out before irradiation (t_0) and 1 hour and 24 hours after starting the irradiation, respectively, and 0.3 mL of CD_3CN added for NMR analysis (refer to **Figure S63**).

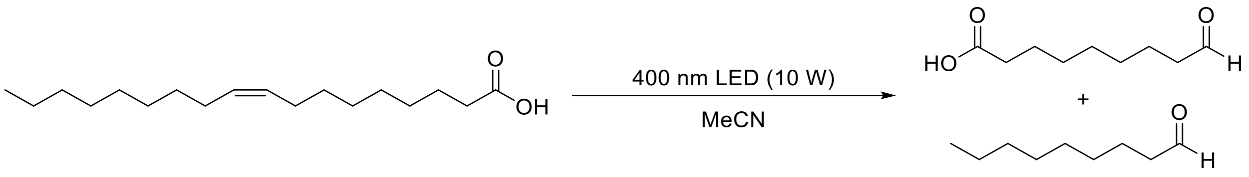

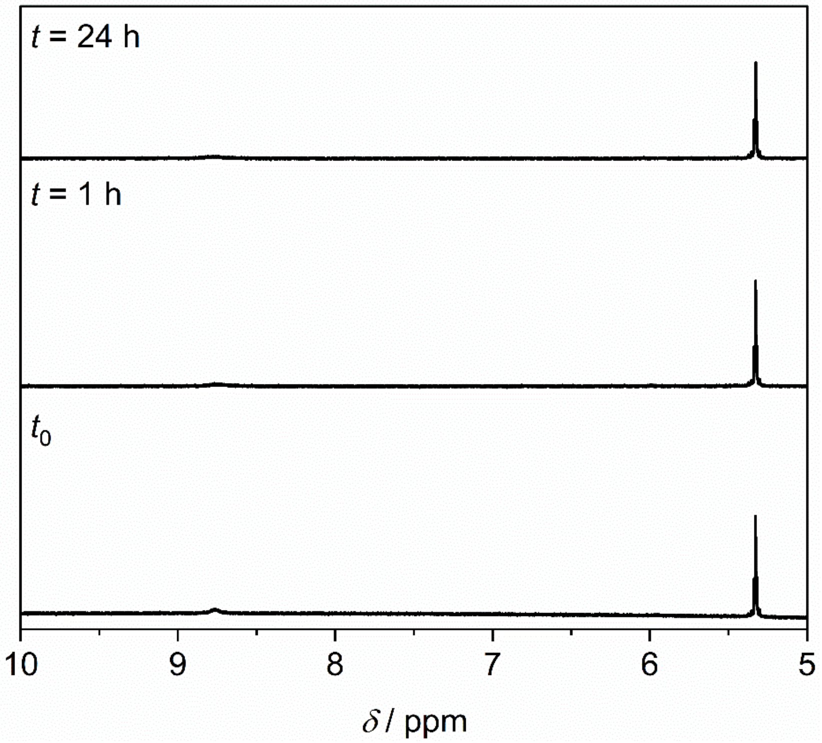

Figure S63 ^{1}H NMR spectra (600 MHz, CD_3CN, 298 K) of oleic acid before (t_0) and at 1 hour and 24 hours after starting the irradiation with a 400 nm LED (10 W, refer to **Figure S27** for the emission spectrum) under oxygen atmosphere.

(B) Unfolded precursor polymer + irradiation

15.6 µL oleic acid (14.1 mg, 49.8 µmol, 1.00 eq.) and 10.0 mg **P1’** (4.98 µmol, 0.10 eq. photoreactive unit) were dissolved in 5 mL of acetonitrile in a 20 mL crimp vial. The resulting colorless solution was sparged with oxygen and an oxygen-filled balloon was fitted on top of the vial. The solution was irradiated using a 400 nm LED (10 W, refer to **Figure S27** for the emission spectrum). 0.1 mL of the reaction solution were taken out before irradiation (t_0) and 1 hour and 24 hours after starting the irradiation, respectively, and 0.3 mL of CD_3CN added for NMR analysis (refer to **Figure S64**).

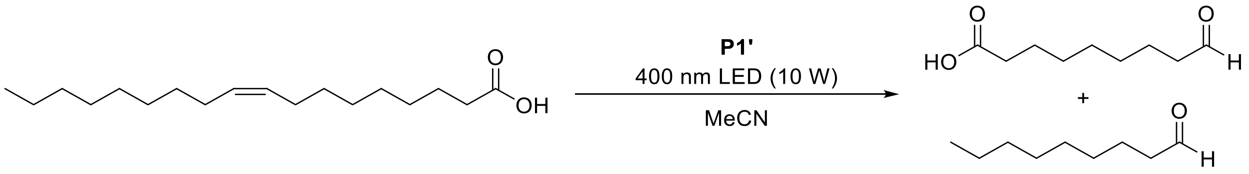

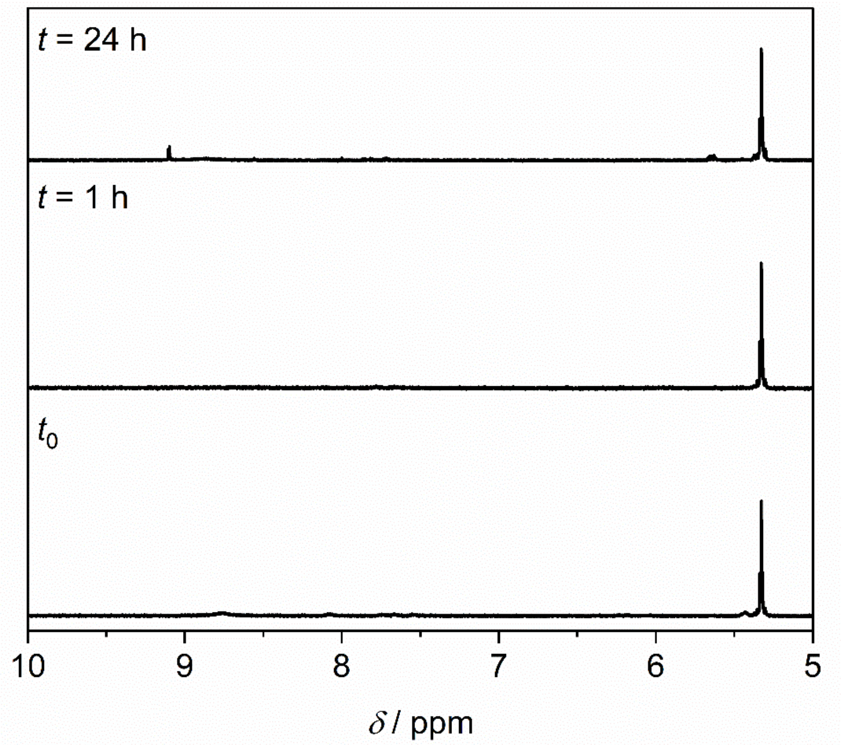

Figure S64 ^{1}H NMR spectra (600 MHz, CD_3CN, 298 K) of the reaction mixture of oleic acid and **P1’** before (t_0) and at 1 hour and 24 hours after starting the irradiation with a 400 nm LED (10 W, refer to **Figure S27** for the emission spectrum) under oxygen atmosphere. Note that the additional resonance apparent after 24 hours is not isochronous to the one observed in the spectra of the catalyzed reactions.

(C) Mixture of unfolded precursor polymer + $CuCl_2 \cdot 2\ H_2O$ subjected to preparative SEC

10.0 mg of polymer **P1'** (4.98 µmol, 0.10 eq. photoreactive unit) and 0.84 mg $CuCl_2 \cdot 2\ H_2O$ (4.98 µmol, 0.10 eq.) were dissolved in 5 mL of acetonitrile. The resulting yellow solution was directly loaded onto a preparative SEC column (Sephadex LH-20) and eluted with acetonitrile, resulting in a colorless solution of **P1'**. The solution was transferred into a 20 mL crimp vial and 15.6 µL oleic acid (14.1 mg, 49.8 µmol, 1.00 eq.) were added. After sparging with oxygen, an oxygen-filled balloon was fitted on top of the vial and the solution irradiated using a 400 nm LED (10 W, refer to **Figure S27** for the emission spectrum). 0.1 mL of the reaction solution were taken out before irradiation (t_0) and after 1 hour and 24 hours, respectively, and 0.3 mL of CD_3CN added for NMR analysis (refer to **Figure S65**).

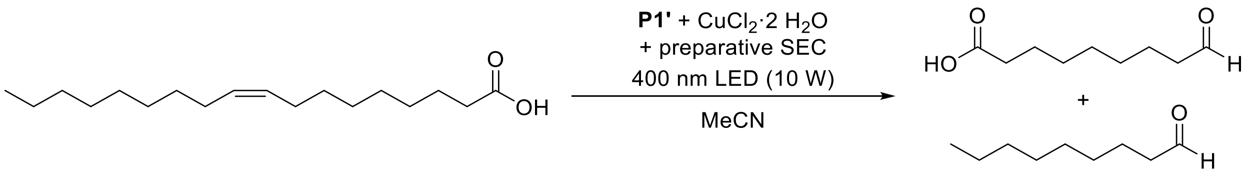

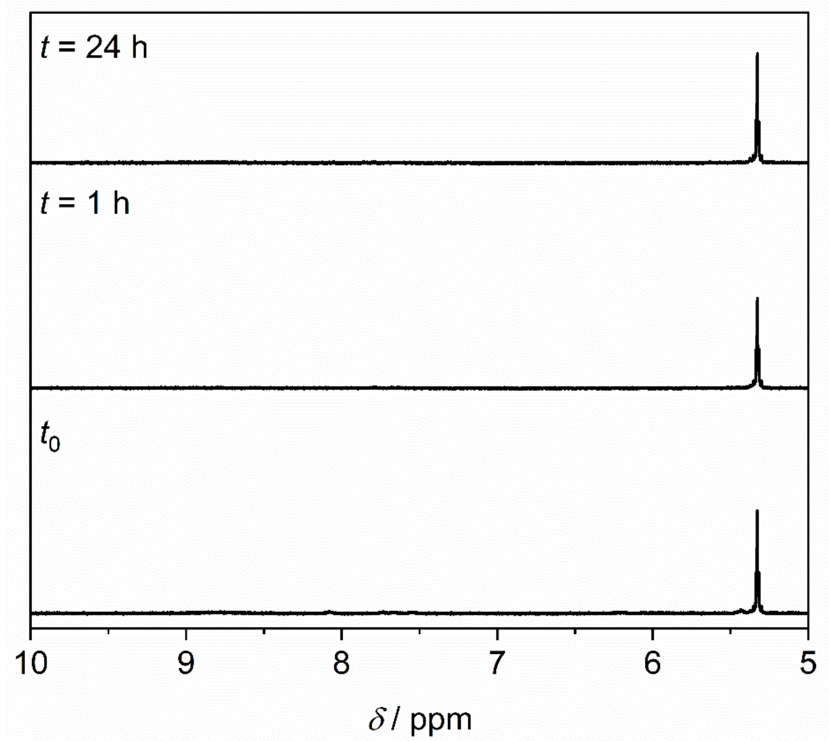

Figure S65 ^{1}H NMR spectra (600 MHz, CD_3CN, 298 K) of the reaction mixture of oleic acid and **P1'** before (t_0) and after 1 hour and 24 hours of irradiation with a 400 nm LED (10 W, refer to **Figure S27** for the emission spectrum) under oxygen atmosphere. For that, **P1'** and $CuCl_2 \cdot 2\ H_2O$ were first mixed and subsequently subjected to preparative size exclusion chromatography (Sephadex LH-20) in acetonitrile, demonstrating that catalytically active $CuCl_2 \cdot 2\ H_2O$ is efficiently removed by this purification technique (compare **Figure S59** to **S61** and **Figure S64**).

(D) $CuCl_2 \cdot 2\ H_2O$ without irradiation

15.6 µL oleic acid (14.1 mg, 49.8 µmol, 1.00 eq.) and 0.84 mg $CuCl_2 \cdot 2\ H_2O$ (4.98 µmol, 0.10 eq.) were dissolved in 5 mL of acetonitrile in a 20 mL crimp vial. The resulting yellow solution was sparged with oxygen and an oxygen-filled balloon was fitted on top of the vial. The solution was stirred at room temperature without irradiation. 0.1 mL of the reaction solution were taken out immediately (t_0) and after 1 hour and 24 hours, respectively, and 0.3 mL of CD_3CN added for NMR analysis (refer to **Figure S66**).

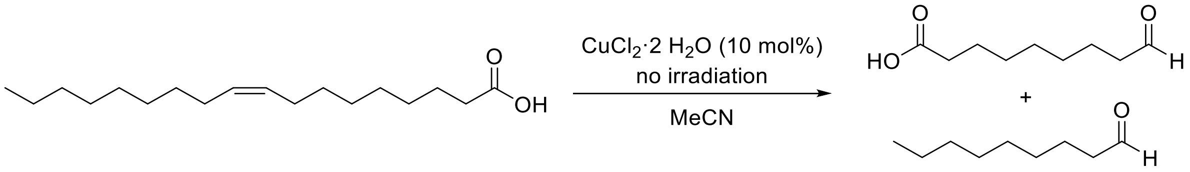

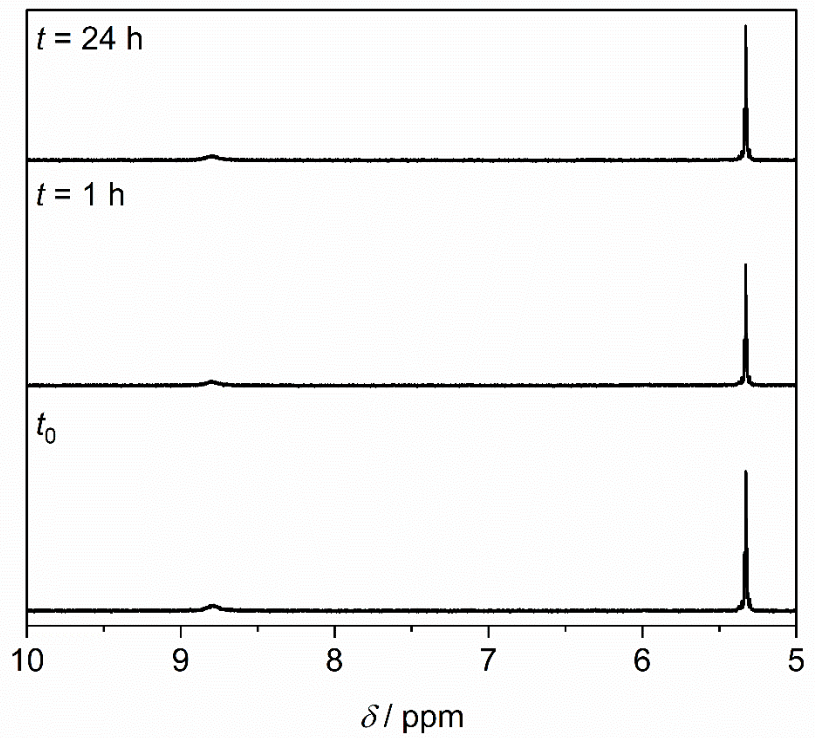

Figure S66 ^1H NMR spectra (600 MHz, CD_3CN, 298 K) of the reaction mixture of oleic acid and 10 mol% $CuCl_2 \cdot 2\ H_2O$ before (t_0) and after 1 hour and 24 hours of stirring at room temperature without irradiation under oxygen atmosphere.

(E) SCNP1-Flow without irradiation

10.0 mg of polymer **P1'** (4.98 µmol, 0.10 eq. photoreactive unit) and 0.84 mg $CuCl_2 \cdot 2\ H_2O$ (4.98 µmol, 0.10 eq.) were dissolved in 5 mL of acetonitrile. The resulting yellow solution was sparged with argon and subsequently irradiated using a 410 nm LED (12 W, refer to **Figure S27** for the emission spectrum) under photoflow conditions (flowrate 0.2 $mL \cdot min^{-1}$, refer to Chapter 2.7 for details regarding the photoflow reactor). The obtained yellow solution was directly loaded onto a preparative SEC column (Sephadex LH-20) and eluted with acetonitrile, resulting in a pale-yellow solution of **SCNP1-Flow**. The solution was transferred into a 20 mL crimp vial and 15.6 µL oleic acid (14.1 mg, 49.8 µmol, 1.00 eq.) were added. After sparging with oxygen, an oxygen-filled balloon was fitted on top of the vial and the solution was stirred at room temperature without irradiation. 0.1 mL of the reaction solution were taken out immediately (t_0) and after 1 hour and 24 hours, respectively, and 0.3 mL of CD_3CN added for NMR analysis (refer to **Figure S67**).

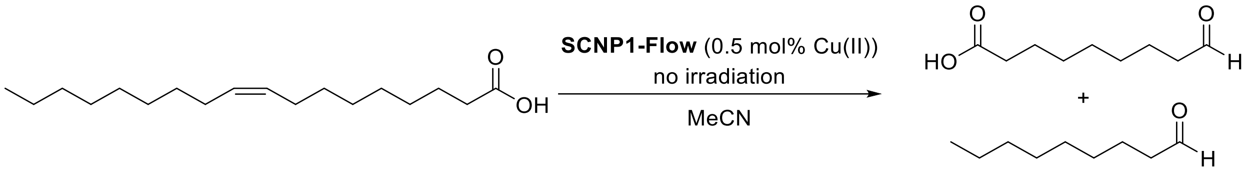

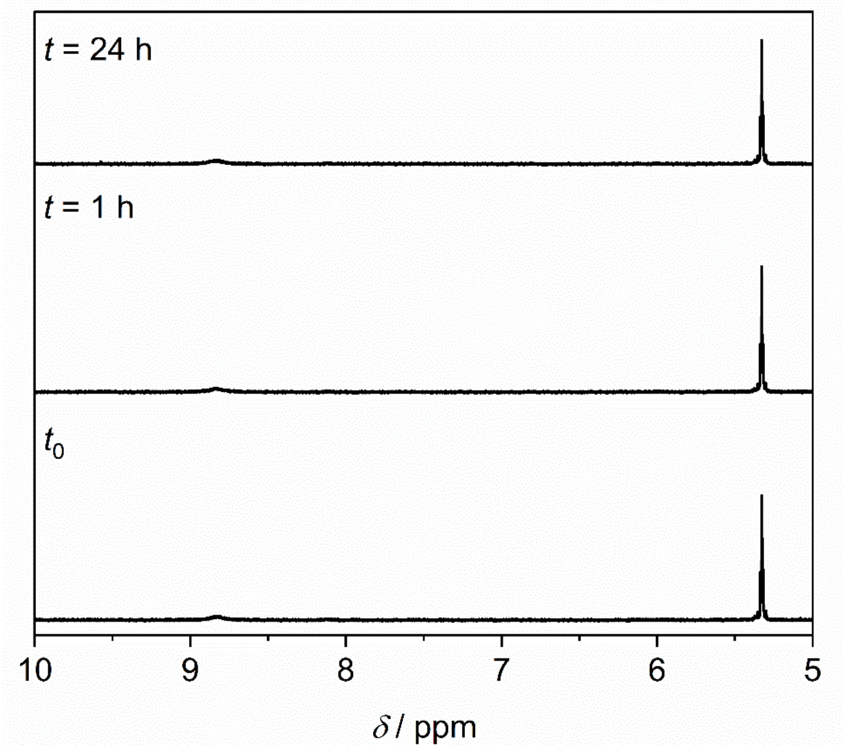

Figure S67 ^1H NMR spectra (600 MHz, CD_3CN, 298 K) of the reaction mixture of oleic acid and **SCNP1-Flow** before (t_0) and after 1 hour and 24 hours of stirring at room temperature without irradiation under oxygen atmosphere.

(F) $CuCl_2 \cdot 2\ H_2O$ + unfunctionalized Poly(PEGMEMA)

15.6 µL oleic acid (14.1 mg, 49.8 µmol, 1.00 eq.) and 10.0 mg Poly(PEGMEMA) were dissolved in 5 mL of a 0.01 mg·mL^{-1} $CuCl_2 \cdot 2\ H_2O$ stock solution (0.293 µmol, 0.00588 eq.) in acetonitrile in a 10 mL crimp vial. The resulting slightly yellow solution was sparged with oxygen and an oxygen-filled balloon was fitted on top of the vial. The solution was irradiated using a 400 nm LED (10 W, refer to **Figure S27** for the emission spectrum). 0.1 mL of the reaction solution were taken out before irradiation (t_0) and after 1, 3, 5 and 24 hours, respectively, and 0.3 mL of CD_3CN added for NMR analysis (refer to **Figure S68**).

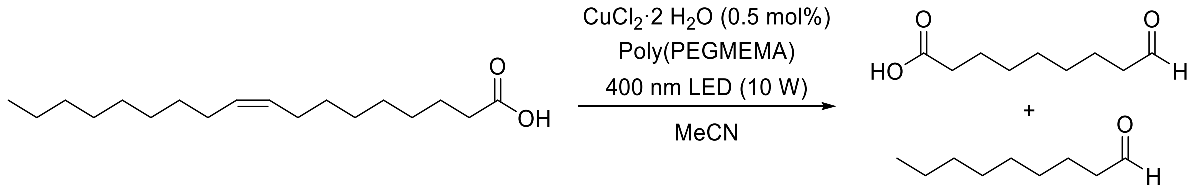

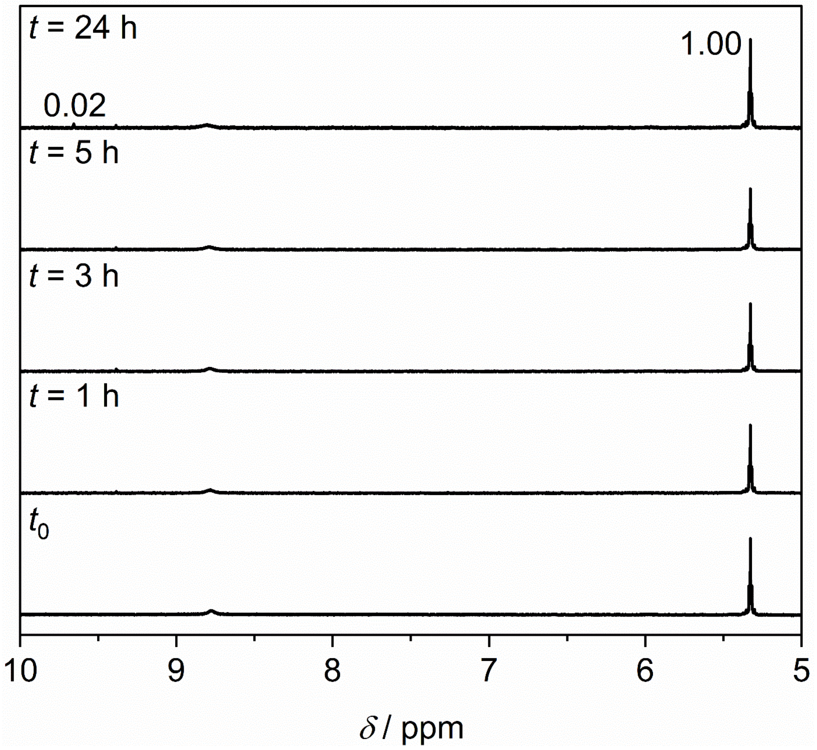

Figure S68 ^{1}H NMR spectra (600 MHz, CD_3CN, 298 K) of the reaction mixture of oleic acid, 0.5 mol% $CuCl_2 \cdot 2\ H_2O$ and Poly(PEGMEMA) before (t_0) at different times after starting the irradiation with a 400 nm LED (10 W, refer to **Figure S27** for the emission spectrum) under oxygen atmosphere. Numbers on peaks denote integral values.

(G) $CuCl_2$·2 H_2O + *n*-Butylamine

A stock solution containing 0.01 mg·mL^{-1} of $CuCl_2$·2 H_2O and 17.2 µg·mL^{-1} *n*-butylamine in acetonitrile was prepared. 15.6 µL oleic acid (14.1 mg, 49.8 µmol, 1.00 eq.) were dissolved in 5 mL of this acetonitrile solution (0.293 µmol, 0.00588 eq. $CuCl_2$·2 H_2O; 1.18 µmol, 0.0235 eq. *n*-butylamine) in a 10 mL crimp vial. The resulting yellow solution was sparged with oxygen and an oxygen-filled balloon was fitted on top of the vial. The solution was irradiated using a 400 nm LED (10 W, refer to **Figure S27** for the emission spectrum). 0.1 mL of the reaction solution were taken out before irradiation (t_0) and after 1, 3, 5 and 24 hours, respectively, and 0.3 mL of CD_3CN added for NMR analysis (refer to **Figure S69**).

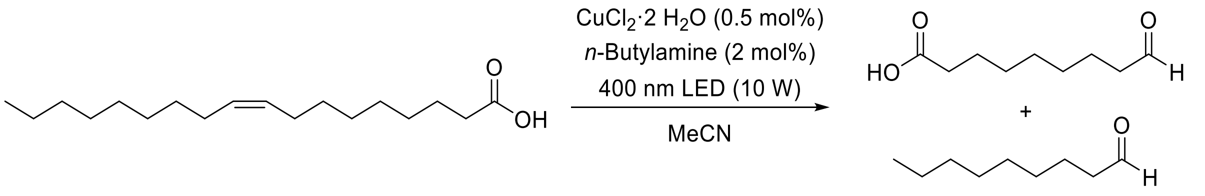

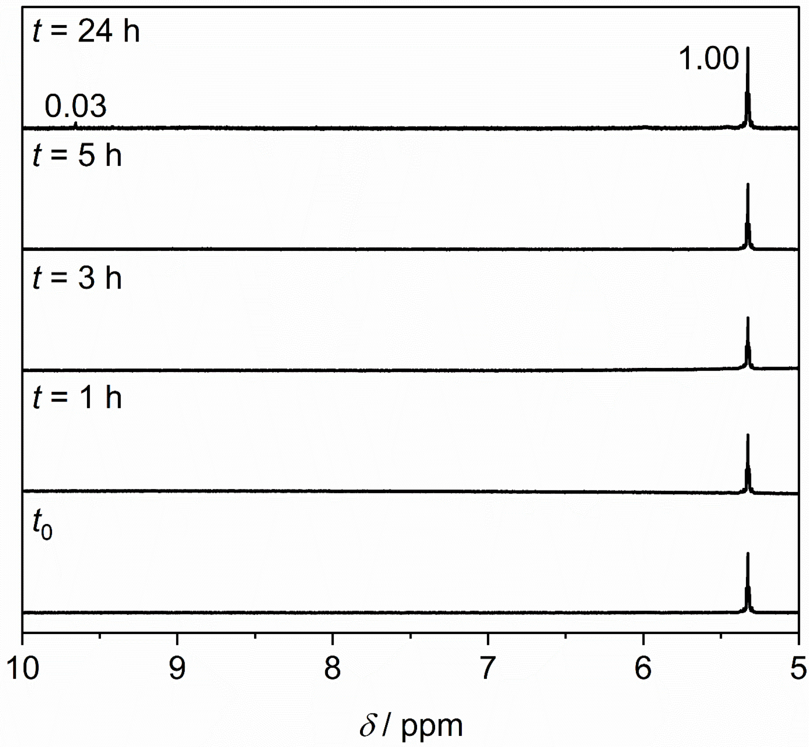

Figure S69 ^{1}H NMR spectra (600 MHz, CD_3CN, 298 K) of the reaction mixture of oleic acid, 0.5 mol% $CuCl_2$·2 H_2O and 2 mol% *n*-butylamine before (t_0) at different times after starting the irradiation with a 400 nm LED (10 W, refer to **Figure S27** for the emission spectrum) under oxygen atmosphere. Numbers on peaks denote integral values.

(H) SCNP1-Batch

10.0 mg of **P1'** (4.98 µmol, 0.10 eq. photoreactive unit) and 0.84 mg of $CuCl_2 \cdot 2\ H_2O$ (4.98 µmol, 0.10 eq.) were dissolved in 5 mL of acetonitrile. The resulting yellow solution was sparged with argon and subsequently irradiated using a 400 nm LED (10 W, refer to **Figure S27** for the emission spectrum) for 50 minutes. The obtained yellow solution was directly loaded onto a preparative SEC column (Sephadex LH-20) and eluted with acetonitrile, resulting in a pale-yellow solution of **SCNP1-Batch**.

The solution was transferred into a 20 mL crimp vial and 15.6 µL oleic acid (14.1 mg, 49.8 µmol, 1.00 eq.) were added. After sparging with oxygen, an oxygen-filled balloon was fitted on top of the vial and the solution irradiated using a 400 nm LED (10 W, refer to **Figure S27** for the emission spectrum). 0.1 mL of the reaction solution were taken out before irradiation (t_0) and after 1, 3, 5 and 24 hours, respectively, and 0.3 mL of CD_3CN added for NMR analysis (refer to **Figure S70**).

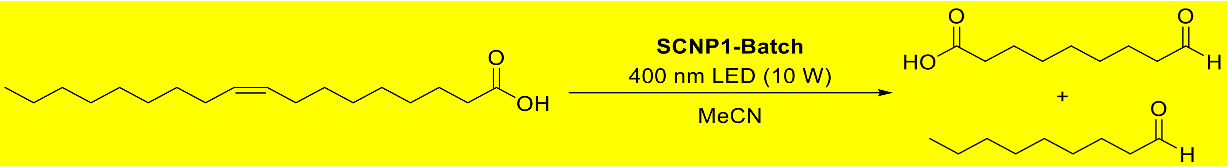

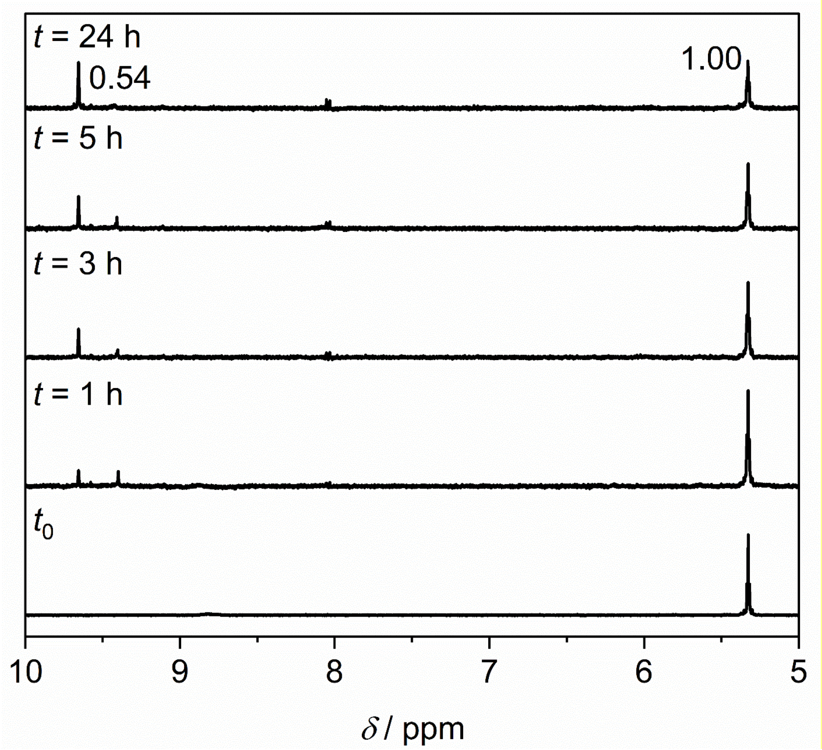

Figure S70 ^{1}H NMR spectra (600 MHz, CD_3CN, 298 K) of the reaction mixture of oleic acid and **SCNP1-Batch** before (t_0) and at different times after starting the irradiation with a 400 nm LED (10 W, refer to **Figure S27** for the emission spectrum) under oxygen atmosphere. Numbers on peaks denote integral values.

6.3 Summary of Catalytic Performance

Table S12 Summary of the results of the catalytic studies described in Chapters 6.1 and 6.2. Given are conversions of xanthene-9-carboxylic acid to 9-xanthenone (column X9CA) and oleic acid to 9-oxononaic acid and nonanal (column OA), respectively, derived from the NMR data provided in Chapters 6.1 and 6.2. Average values are given for experiments performed in triplicate.

	**X9CA**	**OA**
**SCNP1-Flow** (0.5 mol% Cu(II))	100%	37%
**SCNP1-Batch**	100%	35%
0.5 mol% $CuCl_2 \cdot 2\ H_2O$	12%	3%
10 mol% $CuCl_2 \cdot 2\ H_2O$	100%	38%
0.5 mol% $CuCl_2 \cdot 2\ H_2O$ + *n*-butylamine	100%	3%
0.5 mol% $CuCl_2 \cdot 2\ H_2O$ + Poly(PEGMEMA)	Trace amounts	2%
**P1'**	Trace amounts	0%
$CuCl_2 \cdot 2\ H_2O$, no irradiation	0%	0%
**SCNP1-Flow**, no irradiation	0%	0%
No catalyst	Trace amounts	0%

7 References

[1] N. D. A. Watuthanthrige, P. N. Kurek and D. Konkolewicz, *Polym. Chem.*, 2018, **9**, 1557-1561.

[2] I. M. Irshadeen, S. L. Walden, M. Wegener, V. X. Truong, H. Frisch, J. P. Blinco and, C. Barner-Kowollik, *J. Am. Chem. Soc.*, 2021, **143**, 21113-21126.

[3] G. Sheldrick, *Acta Cryst. A*, 2008, **64,** 112-122.

[4] G. Sheldrick, *Acta Cryst. C*, 2015, **71**, 3-8.

[5] O. V. Dolomanov, L. J. Bourhis, R. J. Gildea, J. A. K. Howard and H. Puschmann, *J. Appl. Crystallogr.*, 2009, **42**, 339-341.

[6] E. Blasco, B. T. Tuten, H. Frisch, A. Lederer and C. Barner-Kowollik, *Polym. Chem.,* 2017, **8**, 5845-5851.
